# Global patterns of nuclear and mitochondrial genetic diversity in marine fishes

**DOI:** 10.1002/ece3.11365

**Published:** 2024-05-06

**Authors:** René D. Clark, Malin L. Pinsky

**Affiliations:** ^1^ Department of Biology Drexel University Philadelphia Pennsylvania USA; ^2^ Department of Ecology and Evolutionary Biology University of California Santa Cruz Santa Cruz California USA; ^3^ Department of Ecology, Evolution, and Natural Resources Rutgers University New Brunswick New Jersey USA

**Keywords:** latitudinal gradient, macrogenetics, marine fish, mitochondrial genetic diversity, nuclear genetic diversity, population genetics

## Abstract

Genetic diversity is a fundamental component of biodiversity. Examination of global patterns of genetic diversity can help highlight mechanisms underlying species diversity, though a recurring challenge has been that patterns may vary by molecular marker. Here, we compiled 6862 observations of genetic diversity from 492 species of marine fish and tested among hypotheses for diversity gradients: the founder effect hypothesis, the kinetic energy hypothesis, and the productivity‐diversity hypothesis. We fit generalized linear mixed effect models (GLMMs) and explored the extent to which various macroecological drivers (latitude, longitude, temperature (SST), and chlorophyll‐a concentration) explained variation in genetic diversity. We found that mitochondrial genetic diversity followed geographic gradients similar to those of species diversity, being highest near the Equator, particularly in the Coral Triangle, while nuclear genetic diversity did not follow clear geographic patterns. Despite these differences, all genetic diversity metrics were correlated with chlorophyll‐a concentration, while mitochondrial diversity was also positively associated with SST. Our results provide support for the kinetic energy hypothesis, which predicts that elevated mutation rates at higher temperatures increase mitochondrial but not necessarily nuclear diversity, and the productivity‐diversity hypothesis, which posits that resource‐rich regions support larger populations with greater genetic diversity. Overall, these findings reveal how environmental variables can influence mutation rates and genetic drift in the ocean, caution against using mitochondrial macrogenetic patterns as proxies for whole‐genome diversity, and aid in defining global gradients of genetic diversity.

## Graphical abstract

Genetic diversity is a fundamental component of biodiversity. We outline and test three distinct macroecological drivers of intraspecific genetic diversity, identify global patterns, and assess the congruence of these relationships across the genome to help provide a more complete understanding of how evolutionary forces contribute to biodiversity at multiple scales. Overall, we reveal how environmental variables can influence mutation rates and genetic drift in the ocean, caution against using mitochondrial macrogenetic patterns as proxies for whole‐genome diversity, and aid in defining global gradients of genetic diversity.

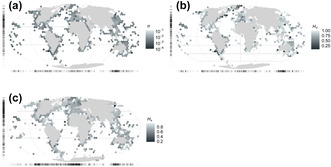

## 1 INTRODUCTION

At its core, genetic diversity is the foundation upon which biodiversity flourishes. Intraspecific genetic diversity can help drive speciation events by enabling adaptation to novel environments and reducing extinction risk by providing a genomic reservoir during periods of environmental change (Vellend & Geber, 2005). Exploring global trends in genetic diversity can shed light on the mechanisms, or combination of mechanisms, that drive spatial and temporal patterns in species diversity. Similarly, elucidating the processes that generate genetic diversity helps create a common ground for evolutionary biology and community ecology around topics of diversity and patterns of speciation. Despite this importance, the general patterns of genetic diversity across species remain poorly understood at global scales (De Kort et al., 2021; Manel et al., 2020; Miraldo et al., 2016).

Much of our knowledge on intraspecific genetic diversity, including local and regional estimates in various taxa, has only been collected in recent decades. Macrogenetic studies have compiled these data to better understand global distributions of genetic diversity (Figuerola‐Ferrando et al., 2023; Miraldo et al., 2016). Large knowledge gaps still exist, however, as the strength and direction of latitudinal gradients in genetic diversity appear to vary across taxa and ecological systems (De Kort et al., 2021). In particular, it remains unclear how universal such patterns are and how influential the underlying ecological drivers may be. This is especially true of marine communities, as most macrogenetic studies to date have focused on terrestrial or freshwater systems (but see Manel et al., 2020).

While the same evolutionary processes occur in all taxa, the strength of these forces differs substantially across the terrestrial and marine realms. Marine species tend to exhibit larger populations, higher gene flow, and wider species ranges (Steele et al., 2019). Alleles may be more easily transported throughout species ranges in marine systems, muting the effects of the local environment and weakening the consequences of genetic drift. Such patterns have previously been documented within individual species, including evidence that strong dispersal helped maintain high diversity in range‐edge populations of Senegal seabream, *Diplodus bellottii* (Robalo et al., 2020). Moreover, global patterns of species richness tend to differ between land and sea. Pelagic marine taxa commonly display bimodal latitudinal gradients of species richness (Tittensor et al., 2010), peaking at mid‐latitudes instead of along the Equator (Worm & Tittensor, 2018). Marine species also have strong longitudinal patterns in species diversity, with the greatest species biodiversity in the Indo‐Pacific Coral Triangle due in part to higher habitat availability and sea surface temperatures (Sanciangco et al., 2013; Tittensor et al., 2010). Given these differences, it remains unclear how environmental conditions and life history strategies in the ocean combine to shape macroecological patterns of genetic diversity. Recent studies have begun to investigate these questions, including Manel et al.'s (2020) finding that mitochondrial genetic diversity in marine fishes is positively correlated with sea surface temperature. However, the mitochondrial genome is a small (<0.01%) fraction of the genetic material in fish that experiences unique evolutionary forces, and more work is needed to understand the ubiquity of these observed patterns across the genome.

Most macrogenetic studies have investigated patterns of mitochondrial genetic diversity, despite suggestions that such markers do not accurately reflect neutral nuclear genetic diversity (Bazin et al., 2006; Leigh et al., 2021). As many mitochondrial markers are linked without recombination to loci under strong selective constraints (Galtier et al., 2009), mitochondrial diversity can be subject to selective sweeps and background selection, as well as bottlenecks due to its small effective population size (*N*_e_), which is a quarter that of nuclear DNA (Birky et al., 1989). Mitochondrial diversity also does not display a consistent relationship with population size, with strong variation across taxa unrelated to life history characteristics (Bazin et al., 2006; Nabholz et al., 2009). With these caveats in mind, macro‐scale patterns of mitochondrial genetic variation may not be generalizable to nuclear diversity. To gain a more complete understanding of global distributions of genetic diversity, neutral genetic variation in the much larger nuclear genome should also be analyzed.

Here, we propose three hypotheses for global genetic diversity gradients, all of which are grounded in foundational community ecology and population genetics theory (reviewed in Worm & Tittensor, 2018). The first is the Kinetic Energy Hypothesis, which posits that, like species richness, intraspecific genetic diversity should be greater at hotter temperatures due to faster evolutionary turnover (e.g., higher metabolic and mutation rates), particularly in mitochondrial DNA that is affected by oxidative damage from metabolic processes (Allen et al., 2002; but see Schmidt & Garroway, 2021). While oxidative damage should not influence nuclear DNA mutation rates (Hoffmann et al., 2004), genome‐wide mutation rates are negatively correlated with generation times (Thomas et al., 2010), which are shorter in organisms with smaller body sizes (Martin & Palumbi, 1993) and, by Bergmann's rule, inversely related to temperature (Bergmann, 1847). Thus, nuclear genetic diversity may also be weakly correlated with temperature (Gillooly et al., 2004). The second hypothesis, the Productivity‐Diversity Hypothesis (Evans et al., 2005), suggests that population size is often constrained by resource availability, such that regions of high primary productivity should support larger populations with greater intraspecific genetic variation since large populations lose genetic diversity to drift at a slower rate (Charlesworth, 2009). However, this relationship may reverse in regions with particularly elevated levels of productivity. Resource availability per species may shrink as more individuals and species compete, causing population sizes and, subsequently, genetic diversity to decline (Lawrence & Fraser, 2020; Storch et al., 2018). Finally, the Founder Effect Hypothesis proposes a negative relationship between latitude and genetic diversity, a lasting legacy from the last glacial maximum (LGM) (Hewitt, 2000). As species expanded out to higher latitudes, a sequential series of founder and bottleneck events along the expansion front may have depleted standing genetic variation and left a latitudinal genetic footprint that is still apparent in many modern populations (Jenkins et al., 2018; Mattingsdal et al., 2020). For marine species, this effect could be particularly pronounced in the Northern hemisphere, as many contemporary high‐latitude taxa in the Southern Ocean endured the LGM in local polar refugia (Fraser et al., 2012).

To help better understand global patterns in marine genetic diversity, we conducted a literature search to aggregate georeferenced data from population genetic studies in marine fish species and then used these data to evaluate our three hypotheses. We compiled environmental data on sea surface temperature (SST) and chlorophyll‐a concentration (a proxy for primary productivity) and assessed the generality of these hypotheses using both mitochondrial and nuclear (microsatellite) DNA. Specifically, we tested (1) the Kinetic Energy Hypothesis that temperature and genetic diversity will be positively related, (2) the Productivity‐Diversity Hypothesis that genetic diversity will be highest in regions with mid‐to‐high levels of primary productivity (e.g., chlorophyll‐a), and (3) the Founder Effect Hypothesis that genetic diversity will be negatively correlated with latitude, particularly in the Northern hemisphere. To test among these hypotheses, we fit generalized linear mixed effect models (GLMMs) and explored the extent to which each macroecological driver explained variation in mitochondrial or nuclear genetic diversity.

## 2 MATERIALS AND METHODS

### 2.1 Data collection

We conducted a literature search on the Web of Science to build a comprehensive database of published genetic diversity observations in marine fishes. The following keyword search terms were used: fish* microsatellite* (marine OR ocean OR sea) and fish* mtDNA* (marine OR ocean OR sea). Only studies published prior to 5 January 2020 were included, and a list of all data sources can be found in the Appendix S3. This was a Class II study in the sense of Leigh et al. (2021) and had the benefits of more easily compiling nuclear diversity data, accounting for methodological covariates that may explain substantial diversity variation, applying more precise data quality filters, and using expert‐defined populations that do not inappropriately split or lump different geographic locations. During data collection, we excluded anadromous, catadromous, and estuarine species, as well as data from populations that were captive, farmed, or stocked. We also excluded data from studies that either did not report the corresponding geographic coordinates, or only vaguely identified the sampling location (precision <3°). For a more detailed explanation of exclusion criteria, see Appendix S1.

We recorded expected heterozygosity (*H*_e_) for microsatellite (nuclear DNA) studies and nucleotide diversity (*π*) or haplotype diversity (*H*_d_) for mitochondrial (mtDNA) studies. When possible, the standard errors of *H*_e_, *H*_d_, or *π* were also documented (or calculated from the standard deviations). All genetic diversity estimates were calculated at the population level. For mtDNA, marker length (in base pairs) was recorded. For microsatellite studies, we recorded whether the primers were originally developed in a different species, as cross‐species amplification can negatively influence diversity estimates (Barbará et al., 2007). When possible, we recorded *H*_e_ on a per‐marker basis, though some studies reported only average *H*_e_ across markers. For these studies, we extrapolated per‐marker diversity by adding a normally distributed error to the average diversity estimate (Pinsky & Palumbi, 2014). This error distribution had a standard deviation (SD) equal to that reported within the study. If within‐study SD was not available, we used the average SD (0.24) across all studies.

In addition to following global patterns, genetic diversity often declines toward a species' range margin, as populations at the edge tend to be smaller in size relative to those at the range center (Clark et al., 2021; Eckert et al., 2008). To help account for these cross‐range effects, which may be distinct from latitudinal effects, we used the “rfishbase” R package v.3.1.6 (Boettiger et al., 2021) to download species range data from Aquamaps (Kaschner et al., 2019). We then calculated the latitudinal range position of each sampled population in our database. This value ranged from 0 to 1, with 0 indicating the population was located at the range center and 1 indicating the population was located at either the northern or southern range edge.

### 2.2 Model structure

We fit GLMMs to test our hypotheses. For models with log‐transformed *π* as the response variable, we ran linear GLMMs with a Gaussian error term using the “lme4” R package v.1.1.26 (Bates et al., 2015). For models with *H*_e_ or *H*_d_ as the response variable, we ran beta GLMMs using the “glmmTMB” R package v.1.1.7 (Brooks et al., 2017). All beta models were run specifying the ordbeta family, which uses a logit link function and enables the incorporation of 0 and 1 values (Kubinec, 2022). For the mtDNA models of *H*_d_, the length of the marker in base pairs was included as an explanatory variable. For the microsatellite models, we included whether the primer was cross‐species amplified. Marker length and cross‐species amplification, as well as range position, were all scaled and centered to have a mean of 0 and a SD of 1. We incorporated the study the data came from as a random intercept for all models to help account for other study‐specific methodological choices, while marker name (the specific mtDNA marker used) was added as a random intercept for the mtDNA models to help account for marker‐specific mutation rates and selective constraints. The marker name was included as a random intercept because we recorded mtDNA genetic diversity from across the mitogenome and did not limit our dataset to COI or cyt‐b markers. Finally, a nested genus/family random intercept was added to all models to account for phylogenetic relationships.

For each diversity metric (*π*, *H*_d_, or *H*_e_), we fit a series of five models to identify global geographic patterns: (1) a baseline model with just the terms and random effects specified above, (2) a latitude model, (3) an absolute latitude model, (4) a longitude model, (4) a latitude and longitude model, and (5) an absolute latitude and longitude model. The latitude and longitude models contained the predictor variable of interest (e.g., latitude, longitude, etc.) as fixed effects in addition to the baseline model structure. Latitude, absolute latitude, and longitude were all scaled and centered (mean 0, SD 1). Latitude was included as a quadratic term to allow a peak in the tropics, while longitude was incorporated as a smoothing spline using the “splines” R package v.4.2.2 (R Core Team, 2023) to account for its circular nature. In addition, we fit random slopes for the geographic predictors to allow the strength and direction of these relationships to vary by family.

We used the same model structure to compare macroecological drivers of genetic diversity. Similar to the geographic models, we fit a series of models that incorporated either annual mean sea surface temperature (SST) (°C), annual mean chlorophyll‐a concentration (mg/m^3^), or both. SST was scaled and centered (mean 0, SD 1), and chlorophyll‐a was log‐transformed and included as a quadratic term. As with the geographic models, we again included random slopes by family. All environmental data were averaged monthly climatologies (9.2 km^2^ resolution, time frame: 2000–2014) extracted from Bio‐ORACLE (Tyberghein et al., 2012) using the “sdmpredictors” R package v.0.2.10 (Bosch & Fernandez, 2021).

### 2.3 Model comparisons

We compared models with both AIC and BIC, as they vary in their criteria for model selection, with BIC penalizing model complexity more heavily and performing slightly better for datasets with large, highly heterogeneous samples (Brewer et al., 2016; Burnham & Anderson, 2004). Marginal and conditional pseudo‐*R* ^2^ values were calculated with the “performance” R package v.0.10.4 (Lüdecke et al., 2021; Nakagawa & Schielzeth, 2013). Within each model, to identify which variables most influenced patterns of genetic diversity, we plotted marginal effects with the “sjPlot” R package v.2.8.12 (Lüdecke, 2021) and examined the *p*‐values of variable coefficients. Model fits and spatial autocorrelation in the residuals were checked with the “DHARMa” R package v.0.4.3 (Hartig, 2021). Moran's *I* was near zero for all models, and no significant spatial autocorrelation (defined as *p* < .05) was found (Appendix S2: Table S2.1). To assess sensitivity to missing and rare data, all models were bootstrapped 1000× with the “boot” R package v.1.3.28 (Canty & Ripley, 2022). All analyses were performed in R v.4.2.3 (R Core Team, 2023).

Finally, to identify whether global patterns varied across taxa, we ran all models on a subset of 10 families (Scombridae, Lutjanidae, Serranidae, Pomacentridae, Sebastidae, Engraulidae, Gadidae, Syngnathidae, Rajidae, and Carcharhinidae), 1 family at a time. These 10 families were chosen because they (1) had a large amount of data (>~30 observations/dataset) and (2) represented a broad range of life history traits.

## 3 RESULTS

### 3.1 Data collection

For our mitochondrial *π* dataset, we compiled 1781 population‐level measurements of genetic diversity, while for *H*_d_, we compiled 1871 diversity measurements. Collectively, these observations came from 239 studies and represented 262 species in 82 families. For microsatellites, we recorded genetic diversity (*H*_e_) from 3210 populations, 578 studies, and 341 species in 86 families. When recorded for the same population, nuclear *H*_e_ was not strongly correlated with either mitochondrial *π* or *H*_d_ (*H*_e_−*π r*_s_ = 0.242; *H*_e_−*H*_d_ *r*_s_ = 0.349), although *π* and *H*_d_ were positively related to each other (*π*−*H*_d_ *r*_s_ = 0.818) (Appendix S2: Figure S2.1). Mean chlorophyll‐a concentration and mean SST were also not strongly correlated with each other (*r*_s_ = −0.316) (Appendix S2: Figures S2.2 and S2.3).

**FIGURE 1:**
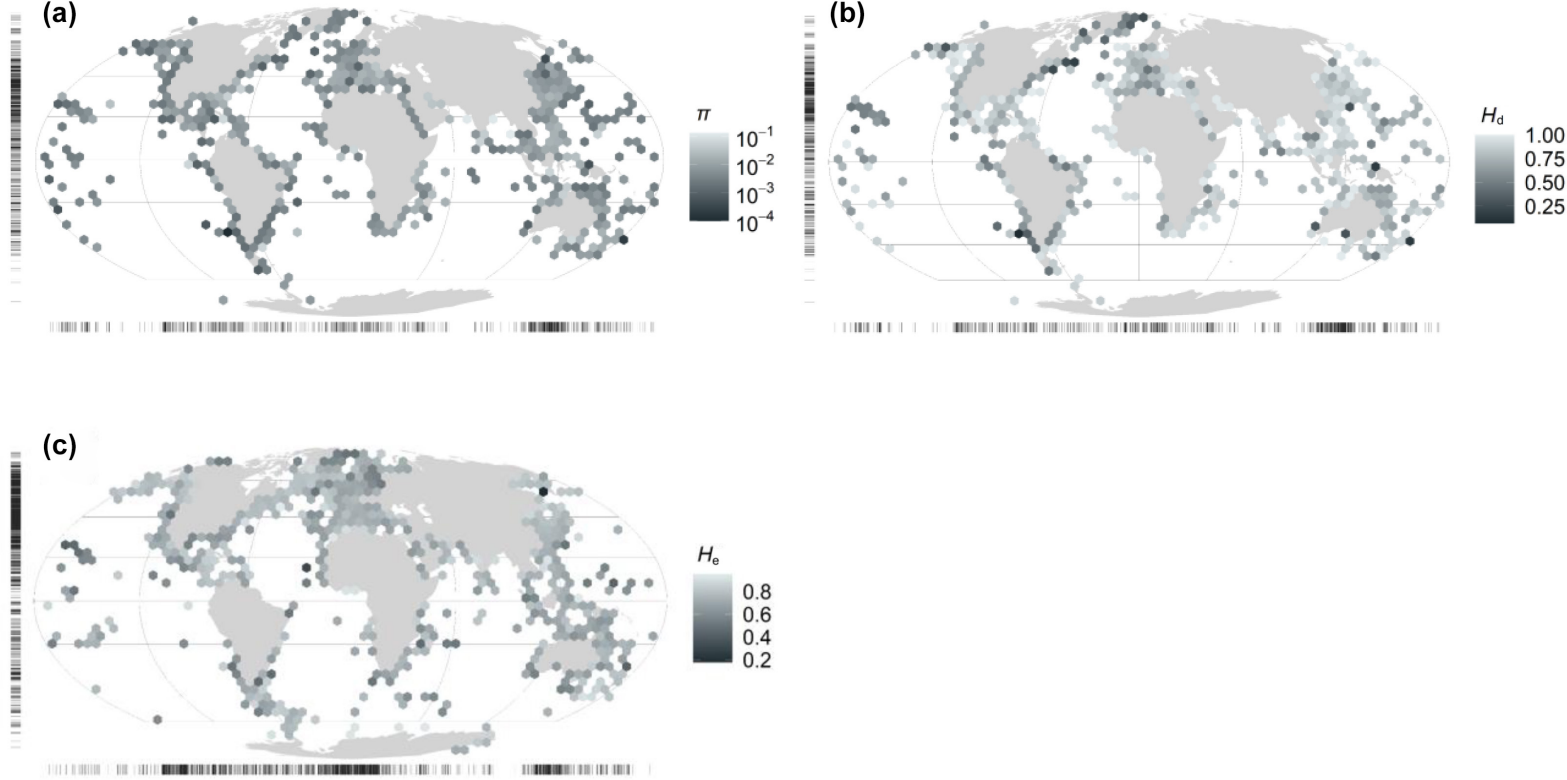
Map of observation locations for mitochondrial (a) *π*, (b) *H*_d_ and nuclear (c) *H*_e_ genetic diversity. Populations were binned into 500 km × 500 km equal‐area grid cells, and the mean species‐wide genetic diversity within each cell was plotted on a Mollweide projection. Rug plots on the *x*‐ and *y*‐axes illustrate the latitudinal and longitudinal sampling locations.

These genetic datasets represented populations from across the globe, spanning all latitudes, every ocean basin, and a wide array of environmental conditions (Figure 1, Appendix S2: Figures S2.4–S2.6). Coastlines in the Northern hemisphere were the most densely sampled regions in our database. However, there were also a large number of diversity estimates near the Equator, particularly in the Coral Triangle. While the number of datapoints decreased toward the poles, there were still a substantial number of observations at latitudes >60°N or S for both mitochondrial (39) and nuclear (311) diversity.

### 3.2 Mitochondrial diversity

**FIGURE 2:**
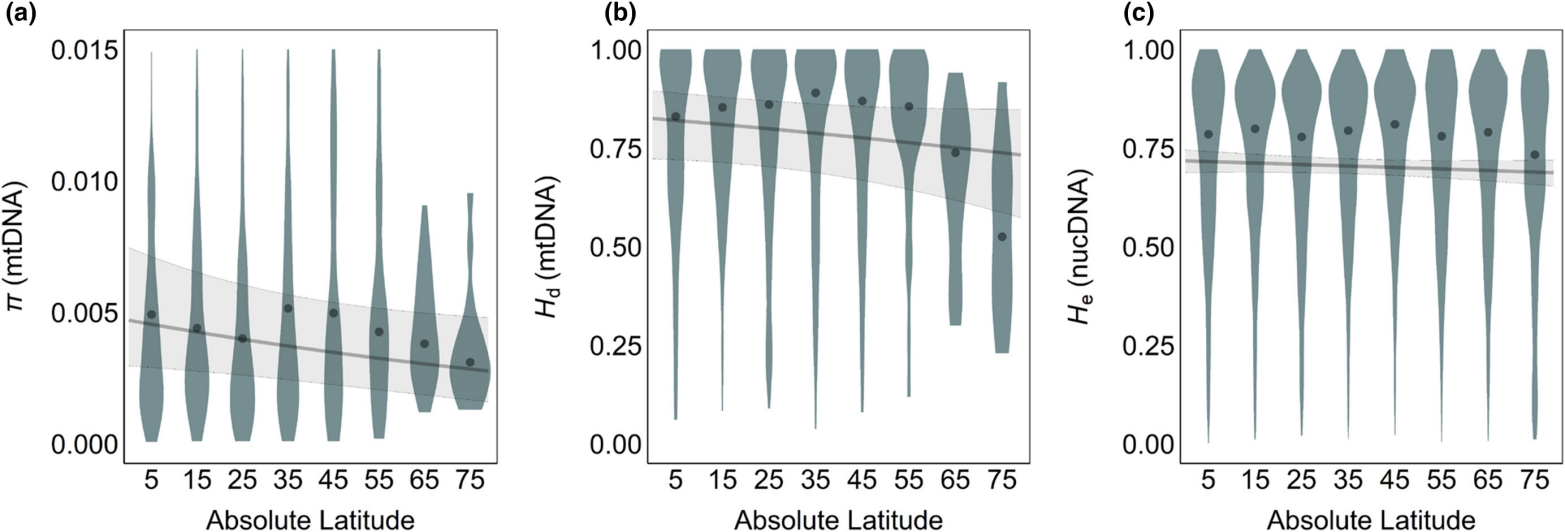
Relationship between absolute latitude and genetic diversity (a) mitochondrial *π*; (b) mitochondrial *H*_d_; (c) nuclear microsatellite *H*_e_. The gray line represents the predicted relationship based on the mixed effects model with shaded 95% confidence intervals. Blue‐gray violin plots show the distribution of genetic diversity binned every 10°, with the dark points representing the medians in every 10° band.

**FIGURE 3:**
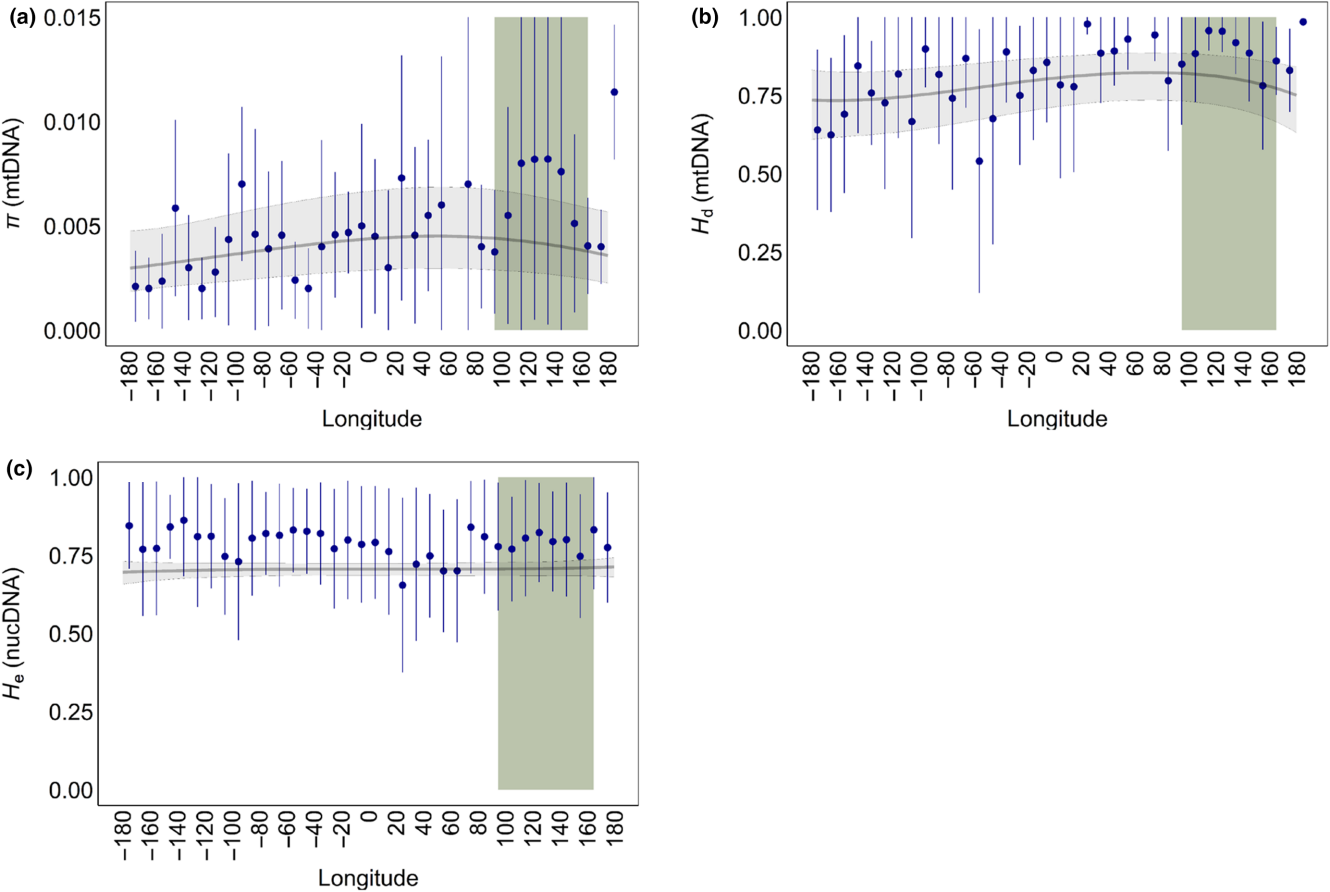
Relationship between longitude and genetic diversity (a) mitochondrial *π*; (b) mitochondrial *H*_d_; (c) nuclear microsatellite *H*_e_. The gray line represents the predicted relationship based on the mixed effects model with shaded 95% confidence intervals. Blue circles represent median diversity binned every 10° with median average deviation (MAD) error bars. The green highlighted region represents the Coral Triangle (longitudes 95–165).

Globally, average mitochondrial genetic diversity was higher in the western Pacific Ocean and lower along North American and European coastlines (Figure 2a,b, Appendix S2: Figure S2.4a,b). For both *H*_d_ and *π*, diversity peaked at low‐to‐mid latitudes and declined toward the poles, particularly in the Northern hemisphere (Figure 2a,b, Appendix S2: Figure S2.7a,b). Diversity was also consistently higher in the Coral Triangle and elsewhere in the western Indo‐Pacific (Figure 3a,b). For mitochondrial genetic diversity (either *H*_d_ or *π*), we found that most latitude and longitude models performed better than the baseline (null) model (Table 1). Latitude and absolute latitude models confirmed patterns of higher mitochondrial diversity toward the Equator (Table 1, Appendix S2: Figure S2.8). As expected, *H*_d_ was positively correlated with marker length (Appendix S2: Figure S2.9) and decreased toward species range edges (although *π* did not do so consistently) (Appendix S2: Figure S2.10).

**TABLE 1:** Mitochondrial DNA (*π* and *H*_d_) model results for latitude and longitude.

Model	bp	Range position	Abslat	Lat (Lat^2^)	Lon	ΔAIC	ΔBIC	*pR* ^2^_C_ [*pR* ^2^_M_]
***π***								
Null		−0.001				0 (645.0)	0 (682.6)	84.42% [0.00%]
Absolute Latitude		0.016	−0.045			−16.8	−6.1	85.48% [0.51%]
Latitude		0.002		−0.038 [−0.014]		−14.6	1.4	85.13% [0.33%]
Longitude		0.001			0.140 0.282*** 0.081	−22.5	−1	84.78% [0.64%]
Absolute Latitude & Longitude		0.012	−0.037*		0.156 0.283** 0.086	−35.9	−3.7	85.74% [0.87%]
Latitude & Longitude		0.005		−0.031 [−0.014]	0.115 0.259*** 0.057	−32.1	5.4	85.42% [0.70%]
***H***_**d**_								
Null	0.379***	−0.062*				0 (−1748.6)	0 (−1694.4)	21.71% [1.22%]
Absolute Latitude	0.371***	−0.017	−0.103			−16.6	−5.8	22.25% [1.28%]
Latitude	0.377***	−0.047		−0.144 [−0.024]		−28.1	−11.9	21.43% [1.34%]
Longitude	0.399***	−0.055*			−0.122 1.160*** 0.081	−24.9	−3.2	21.21% [1.47%]
Absolute Latitude & Longitude	0.394***	−0.026	−0.070		−0.088 1.119*** 0.108	−41.0	−8.5	21.70% [1.47%]
Latitude & Longitude	0.395***	−0.043		−0.118 [−0.016]	−0.219 1.114*** −0.019	−40.9	−2.9	21.06% [1.49%]

*Note*: Standardized model coefficients are reported, along with ΔAIC compared to the null model (model AIC – null AIC), ΔBIC compared to the null model (model BIC – null BIC), and pseudo‐*R* ^2^ values (p*R* ^2^_C_ = conditional pseudo‐*R* ^2^, considers all fixed and random effects; pR^2^_M_ = pseudo‐marginal *R* ^2^, considers only fixed effects). For the null models, AIC and BIC are also reported in parentheses. For latitude, latitude and latitude^2^ were included as predictors in the same model(s). For longitude, the b‐spline basis function coefficients are reported (1–3), each on a different line. *p*‐value: *<.05; **<.01; ***<.001.

**FIGURE 4:**
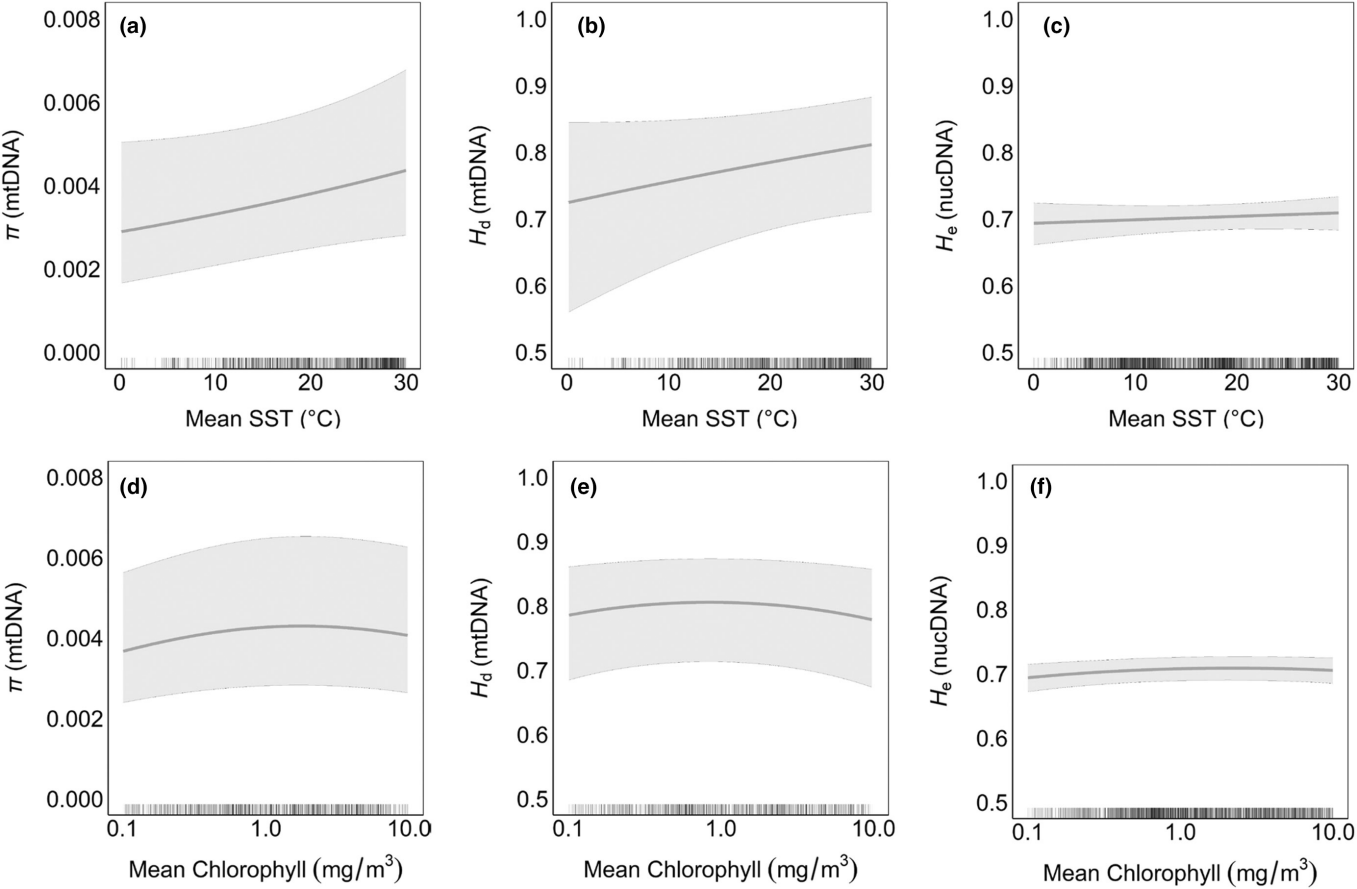
Relationship between mean sea surface temperature (SST) (a–c) or mean chlorophyll‐a concentration (d–f) and genetic diversity (a, d) mitochondrial *π*; (b, e) mitochondrial *H*_d_; (c, f) nuclear microsatellite *H*_e_. The black line represents the predicted relationship based on the mixed effects model with shaded 95% confidence intervals. Rug plots on the *x*‐axis illustrate the SST or chlorophyll‐a sampling extent. The mean chlorophyll‐a concentration is plotted on a common logarithmic scale.

Both environmental drivers were correlated with mitochondrial genetic diversity (*H*_d_ and *π*) (Table 2). Mean SST was positively related with mitochondrial diversity (Figure 4a,b), while chlorophyll‐a concentration followed a quadratic relationship with diversity highest at mid‐to‐upper chlorophyll‐a concentrations (5–10 mg/m^3^) (Figure 4d,e).

**TABLE 2:** Mitochondrial DNA (*π* and *H*_d_) and nuclear (microsatellite *H*_e_) model results for macroecological drivers (mean sea surface temperature (SST) and mean chlorophyll‐a concentration (Chlo)).

Model	Cross spp	bp	Range position	SST mean	Chlo mean [Chlo mean^2^]	ΔAIC	ΔBIC	*pR* ^2^_C_ [*pR* ^2^_M_]
***π* (*mtDNA*)**								
Null			−0.001			0 (645.0)	0 (682.6)	84.42% [0.00%]
SST			0.003	0.042		−21.3	−10.6	85.57% [0.46%]
Chlo			−0.002		0.022 [−0.044]*	−10.2	5.9	84.99% [0.23%]
SST & Chlo			0.006	0.056*	0.041* [−0.050]**	−33.3	−6.5	85.99% [0.76%]
***H***_**d**_ **(*mtDNA*)**								
Null		0.379***	−0.062*			0 (−1748.6)	0 (−1694.3)	21.71% [1.22%]
SST		0.374***	−0.041	0.112		−36.0	−25.2	22.43% [1.32%]
Chlo		0.383***	−0.061*		−0.022 [−0.145]**	−7.2	8.7	21.50% [1.23%]
SST & Chlo		0.370***	−0.035	−0.139	−0.038 [−0.151]**	−39.9	−12.8	22.33% [1.33%]
***H***_**e**_ **(nuclear)**								
Null	−0.072***		−0.007			0 (−11524.8)	0 (−11451.1)	4.85% [0.09%]
SST	−0.072***		−0.005	0.020		−1.7	14.7	5.00% [0.10%]
Chloro	**−**0.071***		−0.007		0.027 −0.039*	−1.8	22.8	4.89% [0.10%]
SST & Chloro	−0.071***		−0.004	0.027	0.028 [−0.038]*	−2.7	38.3	5.02% [0.10%]

*Note*: Model coefficients are reported, along with ΔAIC compared to the null model (model AIC – null AIC), ΔBIC compared to the null model (model BIC – null BIC), and pseudo‐*R* ^2^ values (*pR* ^2^_C_ = conditional pseudo‐*R* ^2^, considers all fixed and random effects; *pR* ^2^_M_ = marginal pseudo‐*R* ^2^, considers only fixed effects). For the null models, AIC and BIC are also reported in parentheses. All model coefficients are standardized except for mean chlorophyll‐a, which was log‐transformed. Furthermore, for chlorophyll‐a models, chlorophyll‐a and chlorophyll‐a^2^ were included as predictors in the same model(s). *p*‐value: *<.05; **<.01; ***<.001.

These global patterns varied substantially across the families represented in our dataset. While the majority of the 10 families we examined separately followed the same overarching patterns (e.g., reduced mitochondrial genetic diversity at higher latitudes, increased diversity at elevated SST, and a quadratic relationship with chlorophyll‐a concentration), several did not (Appendix S2: Figures S2.11–S2.13). Gadidae (cods) and Sebastidae (rockfishes), for example, showed elevated mitochondrial diversity at higher latitudes and lower SST for both *H*_d_ and *π*, while the relationships between latitude and SST for Carcharhinidae (requiem sharks), Engraulidae (anchovies), and Rajidae (skates) differed by metric (*H*_d_ vs. *π*).

### 3.3 Nuclear diversity

In contrast to the mitochondrial results, there was no evidence for strong latitudinal or longitudinal diversity gradients in the nuclear dataset. Nuclear genetic diversity declined weakly toward the poles and did not follow strong longitudinal patterns (Figures 2c, 3c, Appendix S2: Figure S2.7). According to BIC, the null model performed the best, and neither latitude nor longitude was a significant term in any of the models, although AIC selected the latitude model as top‐performing (Table 3). However, diversity was consistently lower for loci amplified with primers originally developed in another species (Appendix S2: Figure S2.14) and showed a negative, albeit non‐significant, trend toward the range edge (Table 3, Appendix S2: Figure S2.10).

**TABLE 3:** Nuclear DNA (*H*_e_) model results for latitude and longitude.

Model	Cross Spp	Range position	Abslat	Lat [Lat^2^]	Lon	ΔAIC	ΔBIC	*p* *R* ^2^_C_ [*p* *R* ^2^_M_]
Null	−0.072***	−0.007				0 (−11524.8)	0 (−11451.1)	4.85% [0.09%]
Absolute Latitude	−0.072***	−0.001	−0.030			−5.0	11.4	5.03% [0.10%]
Latitude	−0.072***	−0.002		−0.053 [−0.011]		−22.2	2.4	5.03% [0.11%]
Longitude	−0.071***	−0.006			0.093 0.003 0.082	−18.4	32.9	4.90% [0.09%]
Absolute Latitude & Longitude	−0.072***	−0.002	−0.025		0.055 0.005 0.062	−19.1	30.1	4.98% [0.10%]
Latitude & Longitude	−0.072***	−0.001		−0.048 [−0.016]	0.023 −0.012 0.061	−24.5	32.9	4.92% [0.11%]

*Note*: Standardized model coefficients are reported, along with ΔAIC compared to the null model (model AIC ‐ null AIC), ΔBIC compared to the null model (model BIC ‐ null BIC), and pseudo‐*R^2^* values (*pR^2^_C_*, conditional psuedo‐*R^2^*, considers all fixed and random effects; *p* *R^2^_M_*, marginal pseudo‐*R^2^*, considers only fixed effects). For the null model, AIC and BIC are also reported in parentheses. For latitude, latitude and latitude^2^ were included as predictors in the same model(s). For longitude, the b‐spline basis function coefficients are reported (1–3), each on a different line. *p*‐value: *<.05; **<.01; ***<.001.

Nuclear diversity was also significantly, albeit weakly, associated with chlorophyll‐a concentration (Table 2). Similarly to the mitochondrial patterns, nuclear genetic diversity peaked at mid‐to‐upper chlorophyll‐a concentrations (5–10 mg/m^3^) (Figure 4f). Mean SST was not significantly related to nuclear genetic diversity, though AIC (but not BIC) weakly selected a model with both SST and chlorophyll‐a (Table 2, Figure 4c).

As with mitochondrial genetic diversity, global patterns in nuclear genetic diversity also varied somewhat across families (Appendix S2: Figures S2.11–S2.13).

## 4 DISCUSSION

Identifying global patterns in biodiversity is a fundamental goal in ecology and evolution but has so far largely focused on variation at the species level (Worm & Tittensor, 2018). Since genetic diversity is a proxy for adaptive potential and the raw material for speciation events, determining its spatial distribution can help explain global patterns in species diversity. Here, we outlined and tested three distinct macroecological drivers of intraspecific genetic diversity, identified global patterns, and assessed the congruence of these relationships across the genome using two distinct molecular markers. Overall, we found that nuclear genetic diversity was significantly correlated with chlorophyll‐a concentration, a proxy for primary productivity and resource availability, while mitochondrial diversity was tightly associated with chlorophyll‐a concentration, SST, latitude, and longitude. Taken together, these results provide support for our original hypotheses to varying degrees. The quadratic relationship between chlorophyll‐a concentration and genetic diversity across the genome provides some evidence for the Productivity‐Diversity Hypothesis and suggests that regions of higher productivity facilitate larger population sizes and, in turn, greater levels of genetic variation. Importantly, our results suggest an optimal level of productivity may exist in this relationship, after which larger carrying capacities may result in reduced population sizes and declining genetic diversity (Storch et al., 2018). Furthermore, environmental temperature was positively correlated with mitochondrial genetic diversity, lending support to the Kinetic Energy Hypothesis. Nuclear diversity displayed no significant relationship with temperature, consistent with the fact that oxidative damage should not impact nuclear DNA (and nuclear DNA mutation rates) to the same degree as mitochondrial DNA (Hoffmann et al., 2004). However, it is important to note that recent research suggests that the connection between temperature, mutation rates, and oxidative stress is complex and nuanced (Schmidt & Garroway, 2021), and that more work is needed to identify the molecular underpinnings of these relationships.

Interestingly, the Founder Effect Hypothesis was the only hypothesis that we did not find clear support for, although the observed decline in mitochondrial genetic diversity toward the poles is in line with its predictions. This decline was particularly pronounced near the Arctic, congruent with the outsized impact of glacial expansion on Northern hemisphere species relative to their Southern Ocean counterparts (Fraser et al., 2012). Furthermore, the smaller *N*_e_ of mitochondrial DNA makes it more sensitive to LGM‐induced bottlenecks (Birky et al., 1989), strengthening any LGM signal in mitochondrial genetic diversity. The high levels of dispersal and admixture often observed in marine systems, along with high *N*_e_, may explain why a similar decline was not observed in nuclear diversity, as elevated dispersal across the species range may help transport genetic diversity and replenish depleted gene pools. In fact, many temperate marine species harbor consistent levels of genetic diversity across their species ranges (Almada et al., 2012; Francisco et al., 2014). Furthermore, in the Northern hemisphere, microrefugia during the LGM that are uncoupled from historical climatic gradients may have “re‐seeded” formerly glaciated regions and buffered northern populations from extirpation, similar to previously documented patterns in the Antarctic (Suggitt et al., 2018). Given that some of these past refugia are close to modern northern range limits, expansion waves out of these locations would have been less susceptible to diversity loss (both nuclear and mitochondrial) from serial founder events (Maggs et al., 2008).

While previous studies have also found latitudinal gradients in mitochondrial genetic diversity, the methods frequently employed by these studies have come under recent criticism (Gratton et al., 2017; Paz‐Vinas et al., 2021). Most earlier macrogenetic studies, especially those investigating patterns in mitochondrial diversity, collected genetic data from shared public resources (e.g., GenBank), pooled sequences into predefined grid cells or latitudinal bands, calculated diversity at the species level, and then averaged species estimates together (Manel et al., 2020; Miraldo et al., 2016; Theodoridis et al., 2020). While informative, studies of this design often struggle to account for genetic variation within species, for the relative frequency of individual haplotypes within populations, for study‐specific methodological choices, or for the unbalanced sampling of species across grid cells (Schmidt & Garroway, 2021). As population size is the mediating factor in many hypotheses aimed at explaining global patterns of genetic diversity, including those assessed here, such distinctions are important. Genetic diversity may follow different spatial patterns at different scales, given that environmental gradients, ecosystem processes, and biogeography collectively influence how population‐level genetic diversity is shaped into community‐wide patterns (De Kort et al., 2021). Here, we conducted a Class II macrogenetic study and reused previously published summary statistics, enabling us to incorporate metadata from the original populations, including sample sizes and the demarcation of local populations (Leigh et al., 2021). This approach allowed us to better account for issues of within‐species geographic variation and relative haplotype abundance.

Despite these differing techniques, our findings also show that mitochondrial diversity follows clear global gradients—peaking at lower latitudes and in the Indo‐Pacific—and reaffirm mitochondrial patterns previously established in Manel et al. (2020). Interestingly, the Coral Triangle has been designated as the center of species biodiversity, especially for coastal species (Worm & Tittensor, 2018), and our models suggest it could play a similar role for genetic diversity, especially within the mitochondria. These results are unsurprising, as several of the predictors we found to be strongly associated with mitochondrial diversity (e.g., SST) have also been linked with higher species richness (Tittensor et al., 2010). Furthermore, coastline length (i.e., habitat availability) has been suggested as a specific driver of species richness in the Coral Triangle and could also increase genetic diversity through its positive influence on population size (Sanciangco et al., 2013). However, our models indicate that other regions in the Indo‐Pacific show elevated mitochondrial genetic diversity as well, including the Indian coastline and Sri Lanka, suggesting other macroecological factors may also play a key role in creating and maintaining genetic diversity.

Importantly, compared to mitochondrial diversity, nuclear genetic diversity did not follow clear geographic patterns. These results are similar to previous studies that saw no strong latitudinal gradients in the nuclear diversity of mammals (Schmidt et al., 2022), freshwater fish (Lawrence et al., 2023), or habitat‐forming species (Figuerola‐Ferrando et al., 2023). As nuclear diversity is tightly coupled with population size, recent demographic processes could have disrupted pre‐existing geographic patterns, muddling any contemporary latitudinal gradients in diversity. When compared to the spatial gradients in mitochondrial genetic diversity, the inconsistency in global patterns across the genome reinforces the message that mitochondrial and nuclear DNA are distinct entities that are separately impacted by evolutionary forces, like drift (via population size) and mutation rates (via kinetic energy). While useful in many circumstances, mitochondrial DNA should be employed with care, and not as a broad and convenient proxy for nuclear markers. This distinction is important because >99.99% of the fish genome is nuclear (Fan et al., 2020; Satoh et al., 2016). Thus, the nuclear genome contains the majority of standing genomic variation important for both adaptation and the speciation process.

Additionally, species‐level variation often reduces statistical power to detect general macro‐scale relationships and almost certainly contributed to the lower psuedo‐*R* ^2^ values reported here (although methodological differences among studies likely played a role as well). Unsurprisingly, we found substantial variation in family‐specific patterns. While most of the suite of 10 families followed the general patterns (at least for mitochondrial diversity) established in the main models, several instead showed increasing genetic diversity at higher latitudes and lower SST. Notably, most of these families (including Gadidae and Sebastidae) are primarily found in colder, more temperate environments that also often have elevated levels of primary productivity. If species at these latitudes are able to support consistently large populations due to higher resource availability and adaptations to colder climates, then genetic diversity and temperature may be negatively correlated within these taxa—a pattern that is apparent in many cold‐adapted species, including pinnipeds, bears, and penguins (Worm & Tittensor, 2018). Moreover, all 10 families displayed either a positive or quadratic relationship with chlorophyll‐a concentration, supporting the key role resource supply and population size play in determining levels of genetic diversity.

Generally speaking, macroecological drivers are likely to act in concert, not in isolation, to shape global patterns. Variation in population size, and subsequently the strength of genetic drift, may establish a baseline distribution of genetic diversity upon which other evolutionary forces interact to create more complex patterns. Both mitochondrial and nuclear genetic diversity peaked in ecosystems with higher resource availability, as represented by primary productivity. In addition, most models suggested genetic diversity was elevated closer to the range core, consistent with the central‐marginal hypothesis that suggests population abundance—and subsequently, genetic diversity—is highest toward the range core where environmental conditions tend to be optimal (Eckert et al., 2008). Layered upon these findings, we found evidence that the higher mitochondrial substitution rates at lower latitudes may serve to replenish and accumulate diversity at lower latitudes, manifesting in a traditional latitudinal gradient for mitochondrial diversity that is highest near the tropics. As nuclear substitution rates are not as clearly elevated at higher temperatures (Hoffmann et al., 2004), similar latitudinal patterns in nuclear genetic diversity were not apparent. Life history traits, anthropogenic change, phylogenetic relationships, and demographic history are also well‐known determinants of genetic diversity, and it is likely these processes influenced our results. For instance, historically, tropical environments tend to be more stable, which can enable diversity at both the species and genetic level to accumulate over time and contribute to the latitudinal diversity gradients observed here (Rosenzweig, 1995). Investigating other nuclear DNA markers (e.g., SNPs, haplotypes) may also help disentangle the relative importance of environmental drivers.

Overall, our results reveal clear global gradients in mitochondrial but not nuclear genetic diversity. While mitochondrial diversity peaks along the Equator and is positively associated with temperature, mirroring complementary patterns in marine species diversity, nuclear genetic diversity shows no strong geographic patterns. Importantly, although these contrasting genomic patterns have been revealed before in different taxa, mitochondrial and nuclear diversity have typically been analyzed with different data types (e.g., nuclear intraspecific population‐level data vs. mitochondrial multi‐species averages). Here, we use the same model structure to compare mitochondrial and nuclear population‐level data, bolstering the argument that these disparate trends are not simply due to methodological artifacts. In particular, such a lack of clear gradients in nuclear diversity may be caused by either evolutionary forces (e.g., contemporary demographic processes disrupting historical patterns, gene flow more evenly distributing alleles across species ranges, or latitudinally consistent mutation rates), analytical ones (e.g., the “noisiness” of microsatellites due to their high polymorphism and ascertainment bias), or a combination of the two. However, despite these differences, diversity across the genome was correlated with chlorophyll‐a concentrations and elevated in regions of higher resource availability that are able to support larger populations. Taken together, these findings enable a better understanding of the degree to which mutation rates (via elevated temperatures) and drift (via population size) work collectively to establish large‐scale gradients of genetic diversity, providing a more comprehensive view of how forces interacting across the genome scale up to provide the raw material for species and ultimately community diversity.

## AUTHOR CONTRIBUTIONS

**René D. Clark:** Conceptualization (equal); data curation (lead); formal analysis (lead); investigation (lead); methodology (equal); software (equal); writing – original draft (lead); writing – review and editing (equal). **Malin L. Pinsky:** Conceptualization (equal); funding acquisition (lead); methodology (equal); software (equal); supervision (lead); writing – review and editing (equal).

### OPEN RESEARCH BADGES

This article has earned Open Data and Open Materials badges. Data and materials are available at https://doi.org/10.5061/dryad.8gtht76wn.

## Supporting information


Appendix S1.

Appendix S2.

Appendix S3.

## Data Availability

The data and scripts for the results presented here are archived on Dryad (https://doi.org/10.5061/dryad.8gtht76wn) and Zenodo (https://zenodo.org/records/11093424).
