# Supplementary material for: Global patterns of nuclear and mitochondrial genetic diversity in marine fishes
Source: Ecol Evol. 2024 May 6;14(5):e11365. doi: 10.1002/ece3.11365 (PMC11070773; doi:10.1002/ece3.11365)
Supplement: Supplementary file 1 — Appendix S1. [file ECE3-14-e11365-s002.pdf]

APPENDIX 1: SUPPLEMENTAL METHODS

DATA COLLECTION AND FILTERING CRITERIA

Web of Science keyword searches were performed using the following keyword search terms: *fish* microsatellite* (marine OR ocean OR sea)* and *fish* mtDNA* (marine OR ocean OR sea)* for microsatellite and mitochondrial DNA studies, respectively. Two rounds of literature searches were conducted. For mitochondrial DNA, the first search took place on January 29, 2013 and the second search on January 5, 2020. For microsatellites, the first search occurred on December 11, 2011 and the second search also occurred on January 5, 2020.

After each search was completed, studies captured in the search were assessed and only the studies meeting a specific set of criteria were kept in the final database. For both nuclear (microsatellite) and mitochondrial DNA studies, only those which measured genetic diversity in marine fishes were kept (anadromous, catadromous, and estuarine species were excluded). Captive, farmed, or stocked populations were also excluded, as were any populations that were identified as hybrids between species. We did not record data from populations with a sample size smaller than 4 individuals or from monomorphic loci. If a study reported multiple temporal samples from the same site, we either recorded data from the replicate (year) with the highest sample size or the most recent sample if there was a tie. If latitudinal and longitudinal coordinates were included in the paper, they were recorded. Otherwise, we used Google Maps to identify approximate geographic coordinates based on sample site names and sampling maps included in the publications. Data was excluded if we could not confidently identify latitudinal and longitudinal coordinates or if the geographic precision was less than 3° latitude or longitude.

For microsatellite studies only, we did not record data from restriction fragment length polymorphisms (RFLPs) or expressed sequence tag (EST)-linked loci. We also excluded loci out of Hardy Weinberg Proportions (HWP). For mitochondrial DNA studies, we excluded data from loci that were shorter than 200 base pairs.

Once filtering was complete, we recorded the following information for each study: scientific name, common name, source (study), the country the sampling site was in, latitudinal and longitudinal coordinates, collection year, and sample size. For microsatellite studies, we recorded the expected heterozygosity (H_e), the standard error (as reported or calculated from standard deviation if possible), whether or note the microsatellite primers were cross-species, the repeat length, and whether or not the study was a primer note. For mitochondrial DNA studies, π and/or haplotype diversity (H_d), the standard error (as reported or calculated from standard deviation), the type of marker (e.g. *Cytb*, *CO1*, etc.), and the length (in base pairs) of the marker was reported. Data was recorded on a per-marker basis when possible (e.x. heterozygosity was recorded for each microsatellite locus individually, not the average across markers). If only average genetic diversity was reported, we also recorded the number of markers included in the average.

Finally, for mitochondrial and nuclear DNA separately, data from each search round were compiled together into one aggregate database (see Figures S2.1 & S2.2 for more information). With microsatellites, the curated database from Pinsky & Palumbi (2014) was also added to the final list of studies. Once all datasets were combined, one final filtering round was completed (using the same criteria as before) to remove any duplicate studies and other data that was missed in the first filtering step.

MICROSATELLITE FILTERING PIPELINE

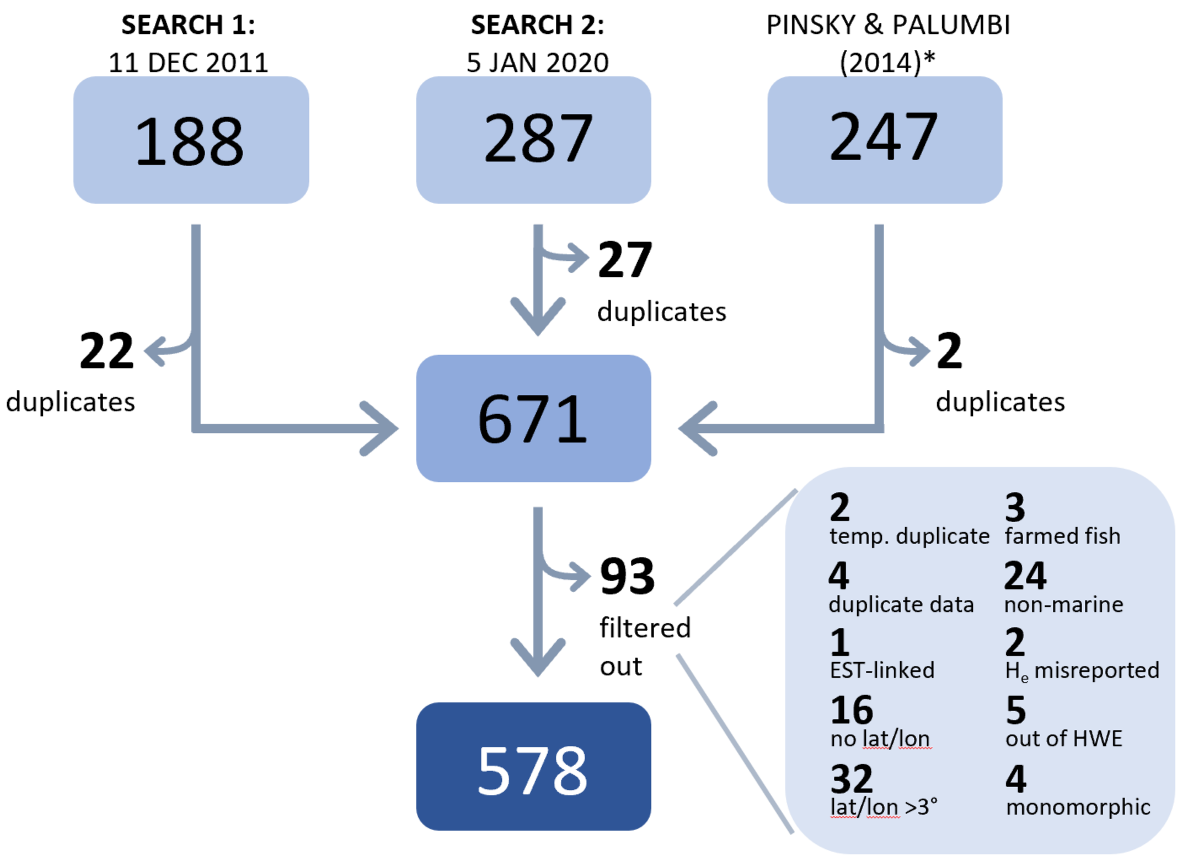

*Pinsky ML & Palumbi SR (2014) Meta-analysis reveals lower genetic diversity in overfished populations. *Molecular Ecology*, **23**, 29-39. doi:10.111/mec.12509

FIGURE S1.1. Study filtering pipeline for microsatellite data. Pipeline details the number of studies originating from the two Web of Science searches, as well as the number of studies from the Pinsky & Palumbi (2014) dataset that was included in the database. Studies were filtered during the initial searches; the downstream filtering pipeline represents the number of additional studies that were removed after all datasets were merged together.

MITOCHONDRIAL DNA FILTERING PIPELINE

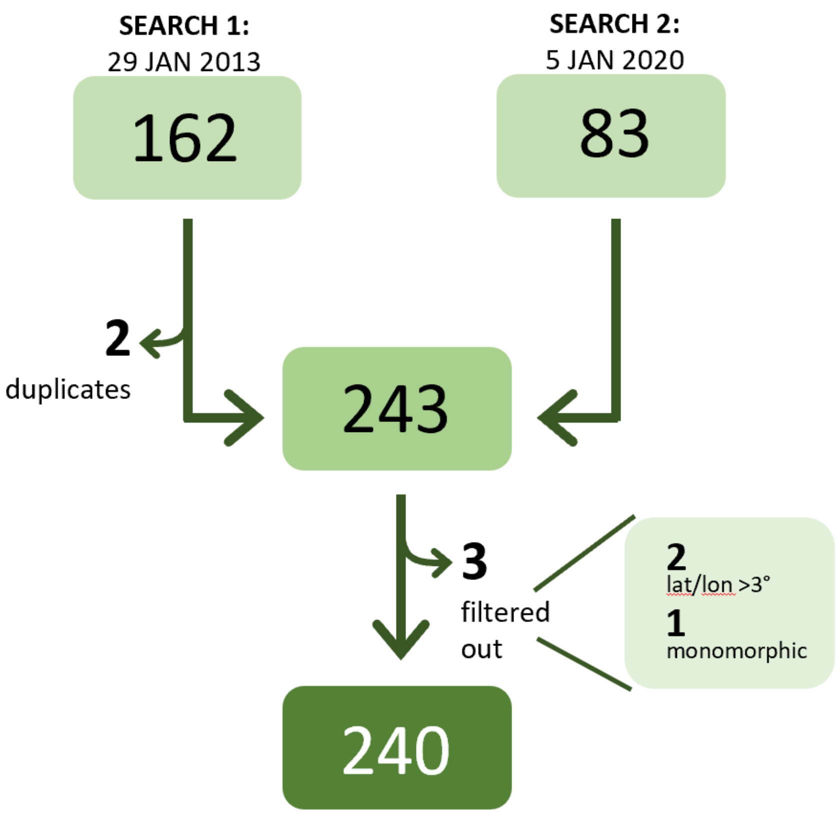

FIGURE S1.2. Study filtering pipeline for mitochondrial data. Pipeline details the number of studies originating from the two Web of Science searches. Studies were filtered during the initial searches; the downstream filtering pipeline represents the number of additional studies that were removed after all datasets were merged together.
