# Supplementary material for: Global patterns of nuclear and mitochondrial genetic diversity in marine fishes
Source: Ecol Evol. 2024 May 6;14(5):e11365. doi: 10.1002/ece3.11365 (PMC11070773; doi:10.1002/ece3.11365)
Supplement: Supplementary file 2 — Appendix S2. [file ECE3-14-e11365-s001.pdf]

## APPENDIX 2: SUPPLEMENTAL INFORMATION

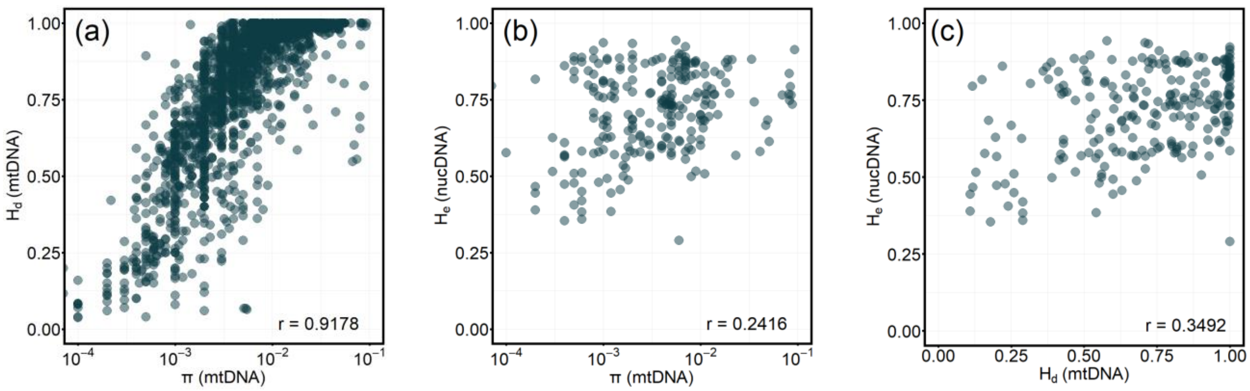


**Figure S2.1.** Pairwise scatterplots of each measure of genetic diversity (mtDNA $H_d$ and π, and microsatellite $H_e$). a: π v. $H_d$ , b: π v. $H_e$, c: $H_d$ v. $H_e$. Points represent diversity within one population (e.g. $H_e$ & $H_d$ measured in the same location/species and reported in the same study). π is plotted on a common logarithm scale. R represents Spearman's correlation coefficient.

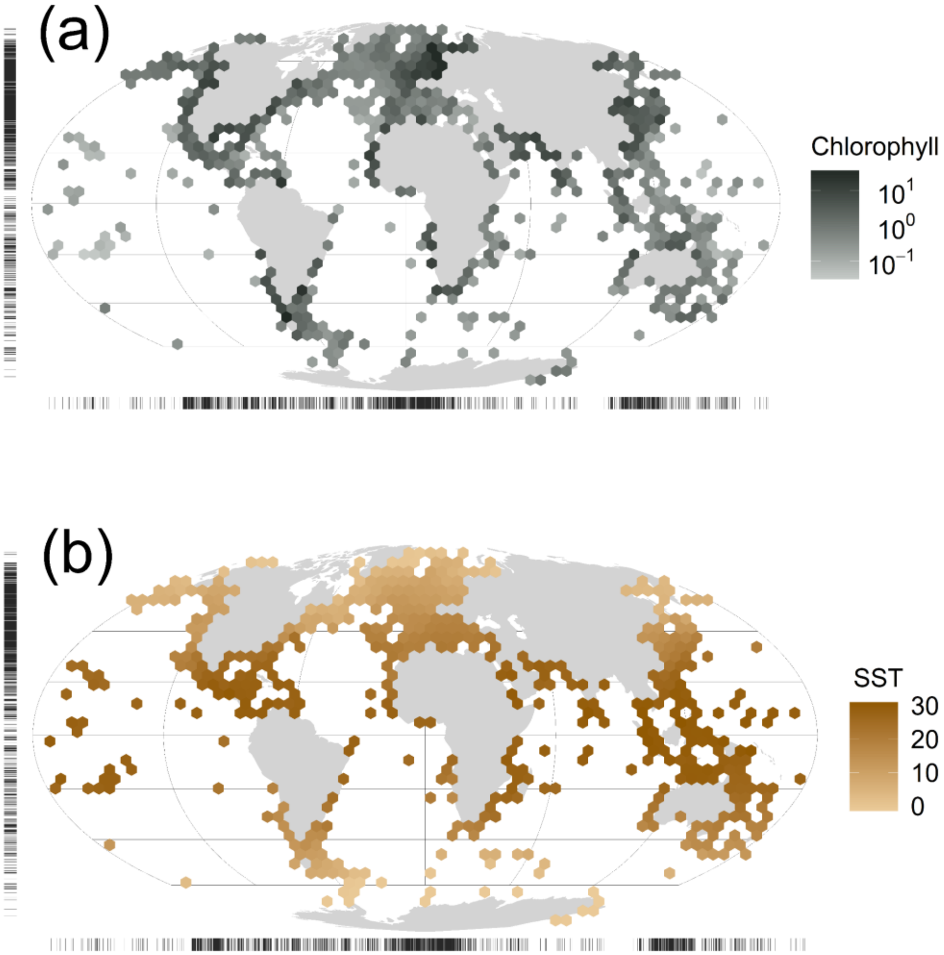


**Figure S2.2.** Map of (a) mean chlorophyll-a concentration ($mg/m^3$) or (b) mean sea surface temperature (SST, °C) for nuclear $H_e$ observations. Values were binned into 500 km x 500 km equal-area grid cells and mean chlorophyll-a concentration or SST within each cell is plotted on a Mollweide projection.

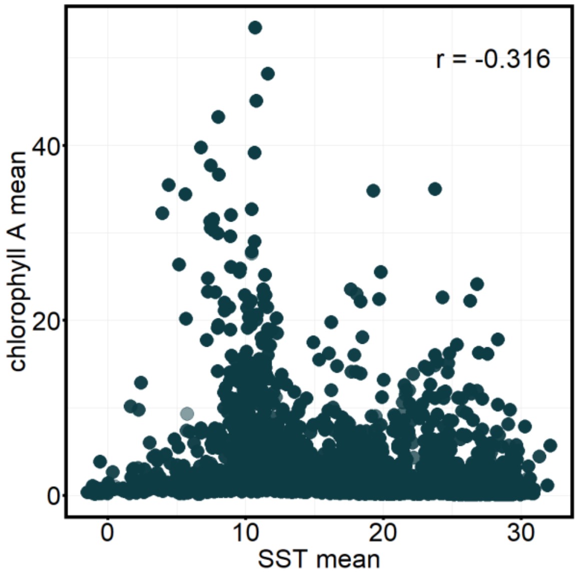


**Figure S2.3.** Pairwise scatterplots of mean chlorophyll A (mg/m$^3$) and mean sea surface temperature (SST, °C) for nuclear $H_e$ observations. R represents Spearman's correlation coefficient.

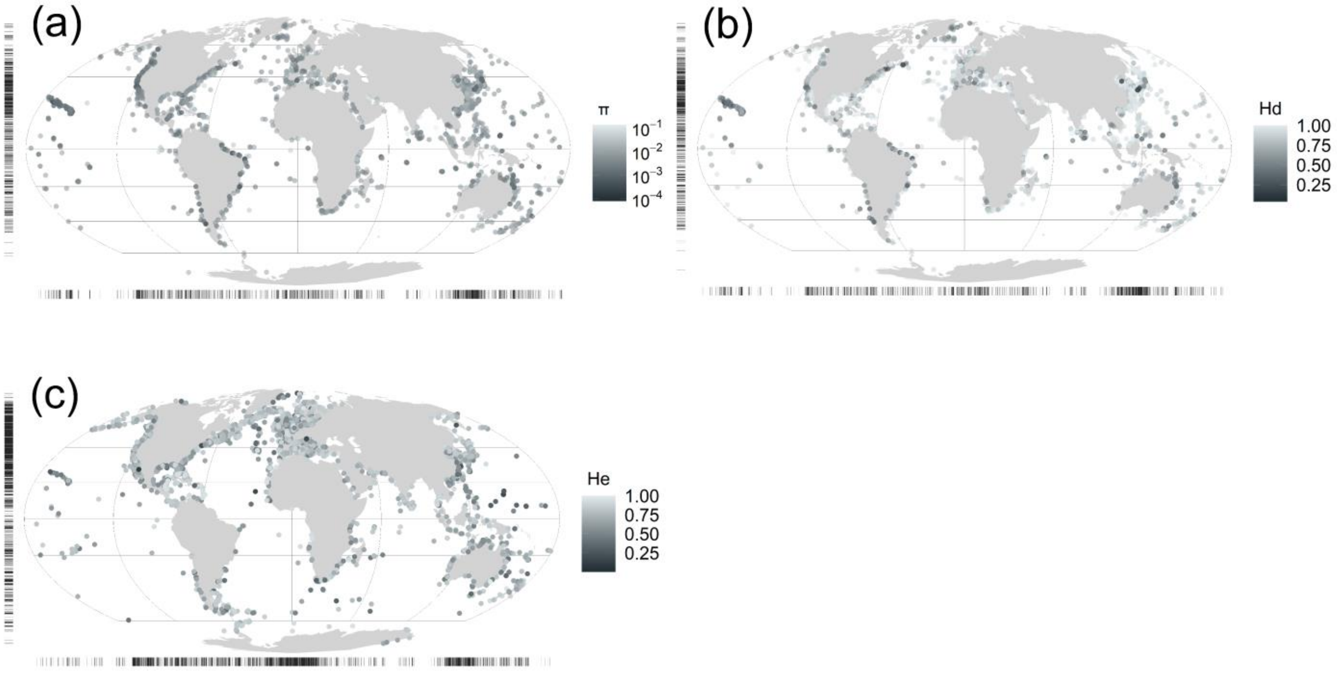


**Figure S2.4.** Map of observation locations for mitochondrial (a: π, b: $H_d$) and nuclear (c: $H_e$) genetic diversity, plotted on a Mollweide projection. Each point represents a unique observation (population). Rug plots on the x and y-axis illustrate the latitudinal and longitudinal sampling extent.

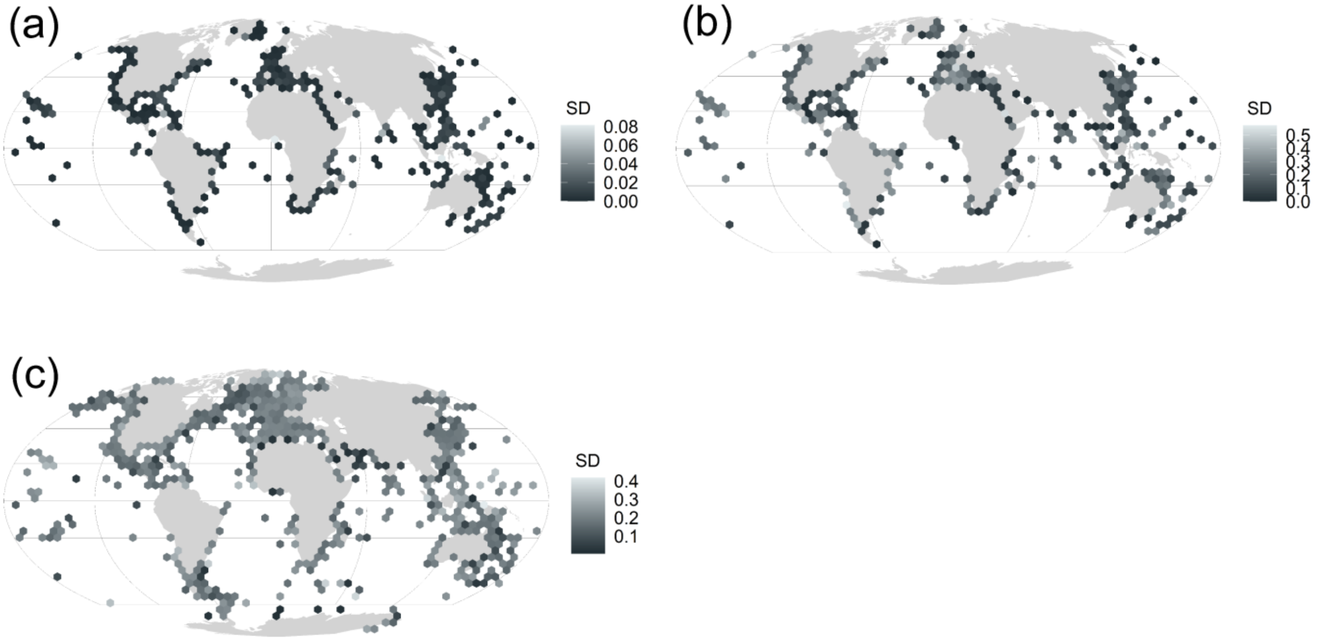


**Figure S2.5.** Map of observation locations for mitochondrial (a: π, b: $H_d$) and nuclear (c: $H_e$) genetic diversity. Populations were binned into 500 km x 500 km equal-area grid cells and the standard deviation (SD) of species-wide genetic diversity within each cell was plotted on a Mollweide projection.

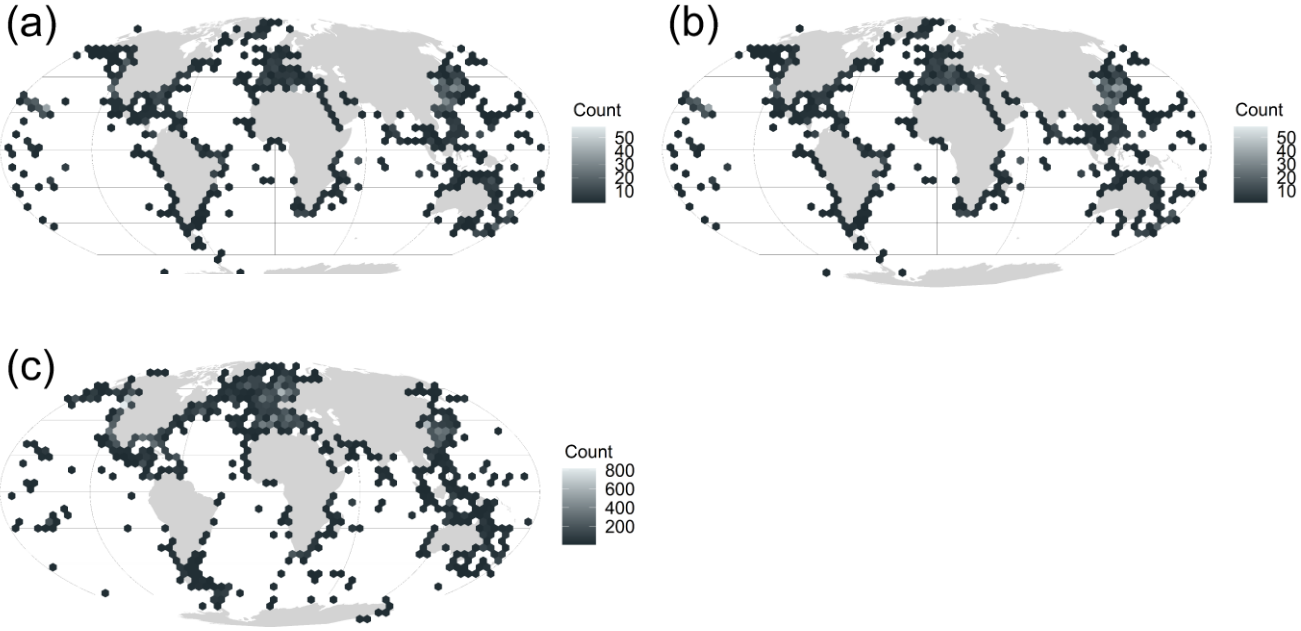


**Figure S2.6.** Map of observation locations for mitochondrial (a: $\pi$, b: $H_d$) and nuclear (c: $H_e$) genetic diversity. Populations were binned into 500 km x 500 km equal-area grid cells and the count of observations within each cell was plotted on a Mollweide projection.

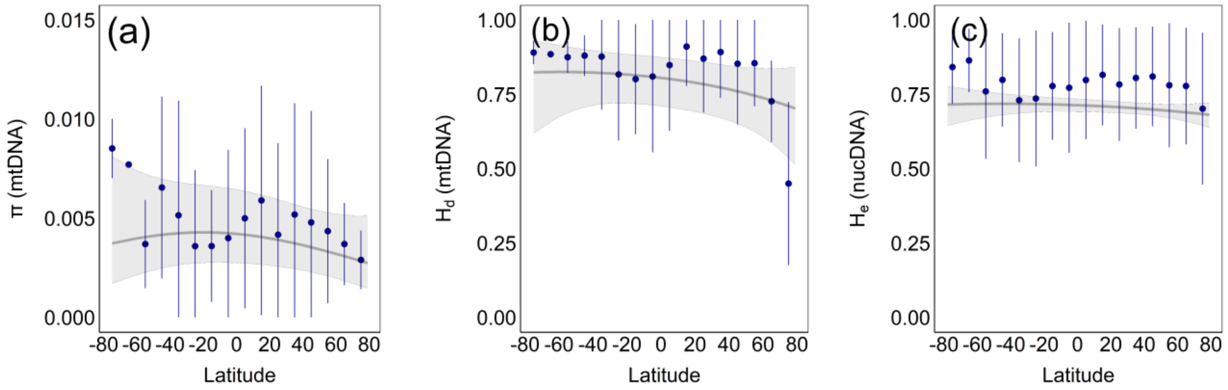


**Figure S2.7.** Relationship between latitude and genetic diversity (a: mitochondrial π; b: mitochondrial $H_d$; c: nuclear microsatellite $H_e$). Gray line represents the predicted relationship based on the latitude mixed effects model with shaded 95% confidence intervals. Blue circles represent median diversity binned every 10° with median average deviation (MAD) error bars.

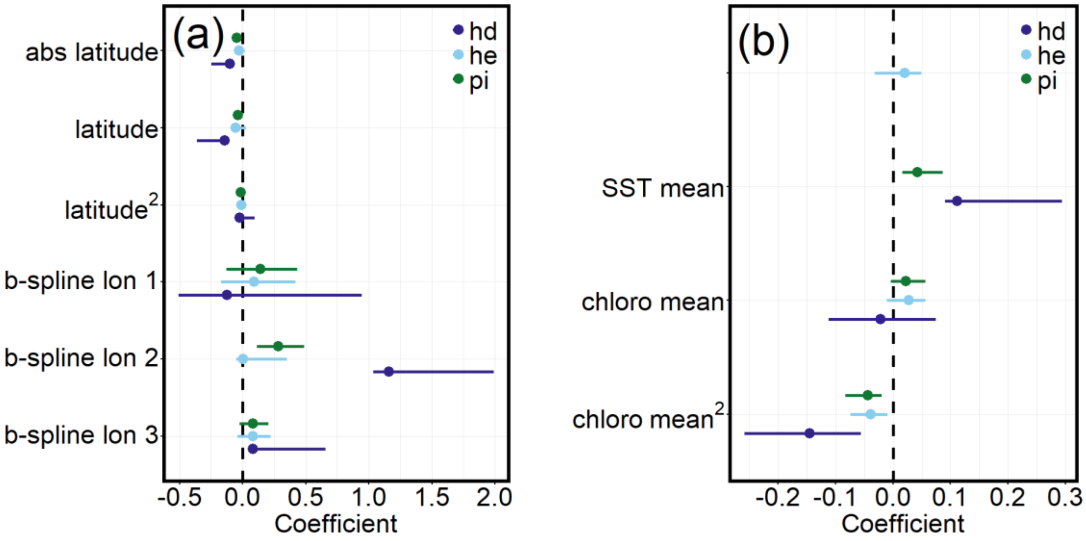


**Figure S2.8.** Model coefficients for all models of mitochondrial $H_d$, mitochondrial $\pi$, and microsatellite $H_e$. Error bars represent bootstrapped 95% confidence intervals. All coefficients are standardized, except for mean chlorophyll-a concentration, which represents the log-odds. For the latitude and longitude models (a), the coefficient estimates and confidence intervals come from the respective model with only one geographic predictor (i.e. not the model with both absolute latitude and longitude). For the SST and mean chlorophyll-a concentration models (b), the coefficient estimates and confidence intervals come from the respective model with only either SST or chlorophyll-a concentration.

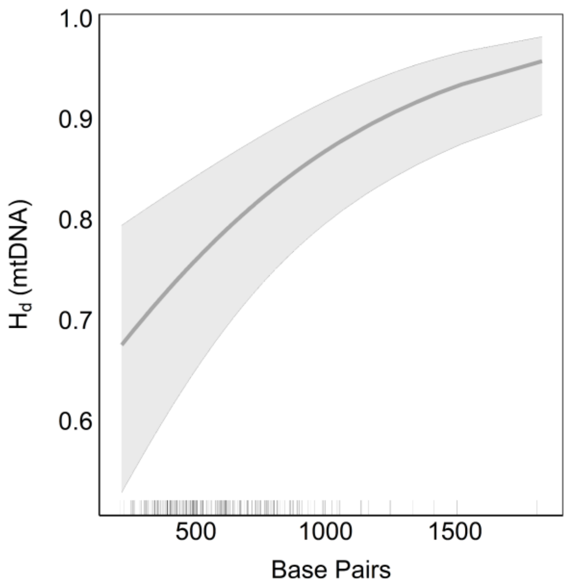


**Figure S2.9.** Relationship between base pair length and mitochondrial $H_d$. Gray line represents the predicted relationship based on the mixed effects model (null) with shaded 95% confidence intervals. Rug plot on the x-axis illustrates the base pair sampling extent.

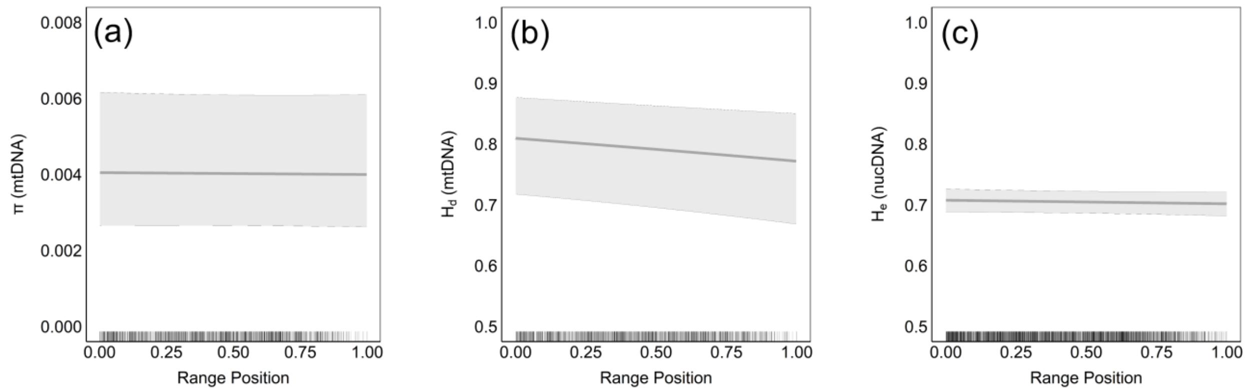


**Figure S2.10.** Relationship between range position and genetic diversity (a: mitochondrial π; b: mitochondrial $H_d$; c: nuclear microsatellite $H_e$). Gray line represents the predicted relationship based on the mixed effects model (null) with shaded 95% confidence intervals. Rug plots on the x-axis illustrate the sampling extent.

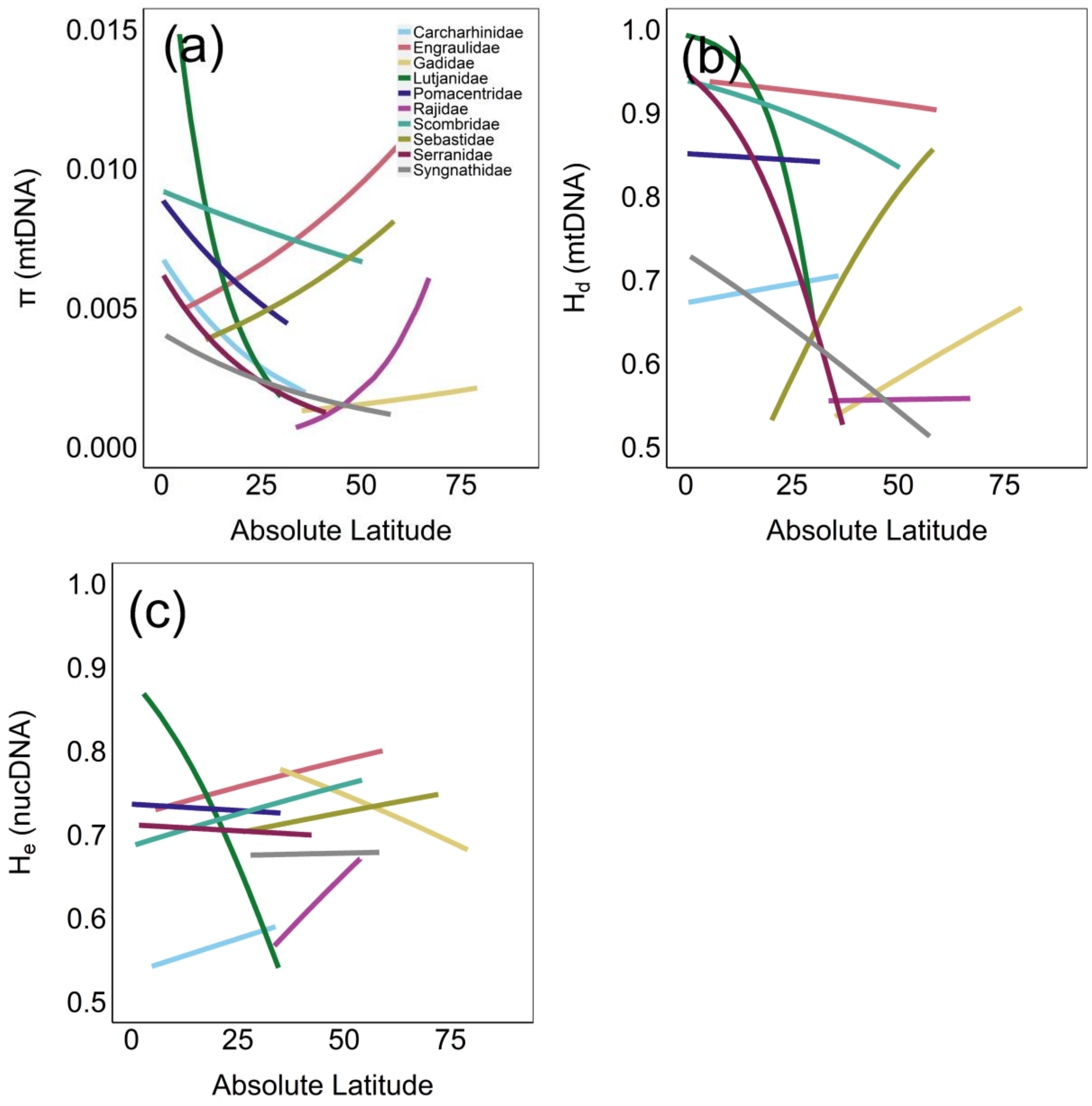


**Figure S2.11.** Relationship between absolute latitude and genetic diversity for 10 families (a: mitochondrial π; b: mitochondrial $H_d$; c: nuclear microsatellite $H_e$).

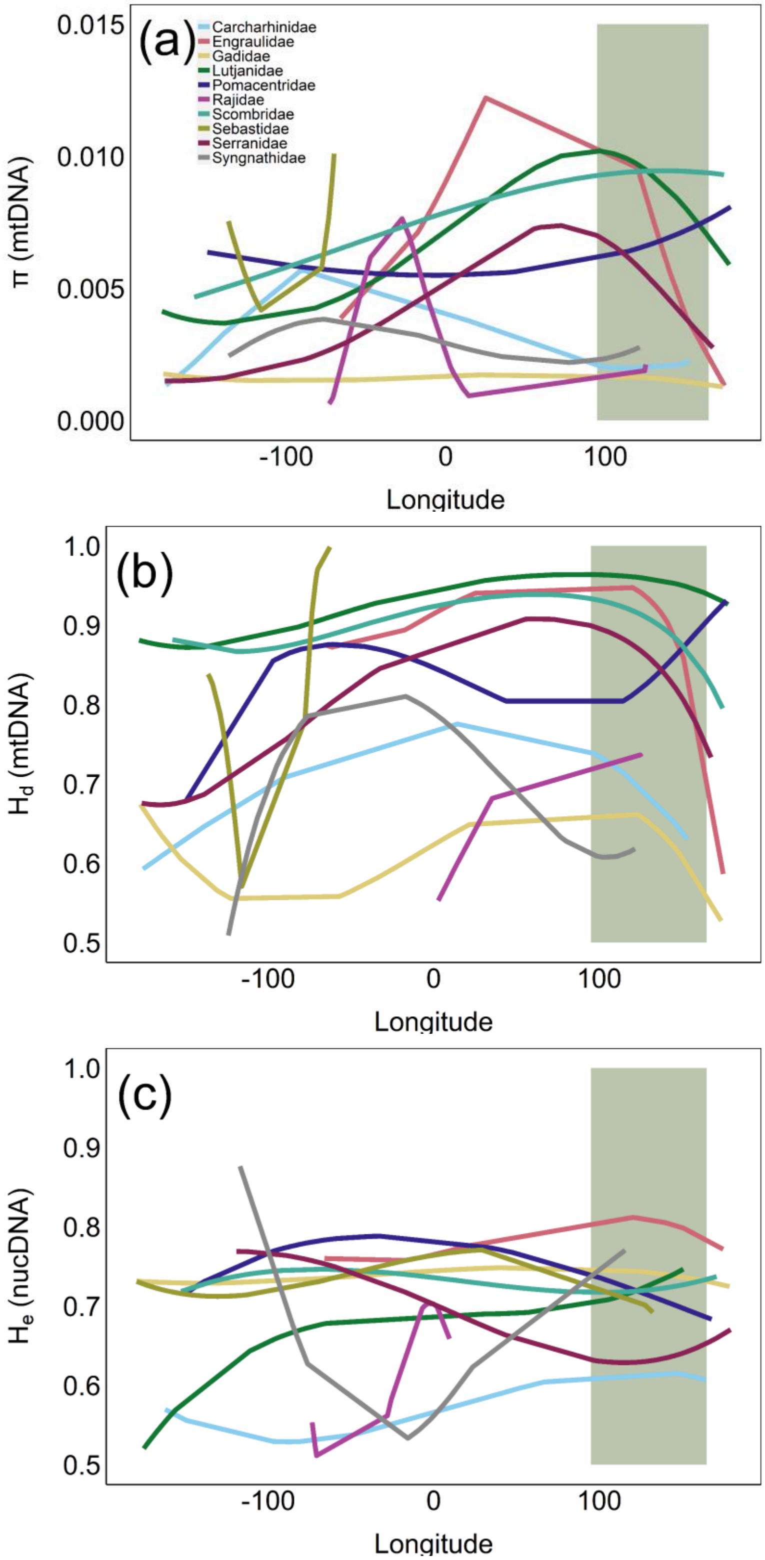


**Figure S2.12.** Relationship between longitude and genetic diversity for 10 families (a: mitochondrial π; b: mitochondrial $H_d$; c: nuclear microsatellite $H_e$). Green highlighted region represents the Coral Triangle (longitudes 95 - 165).

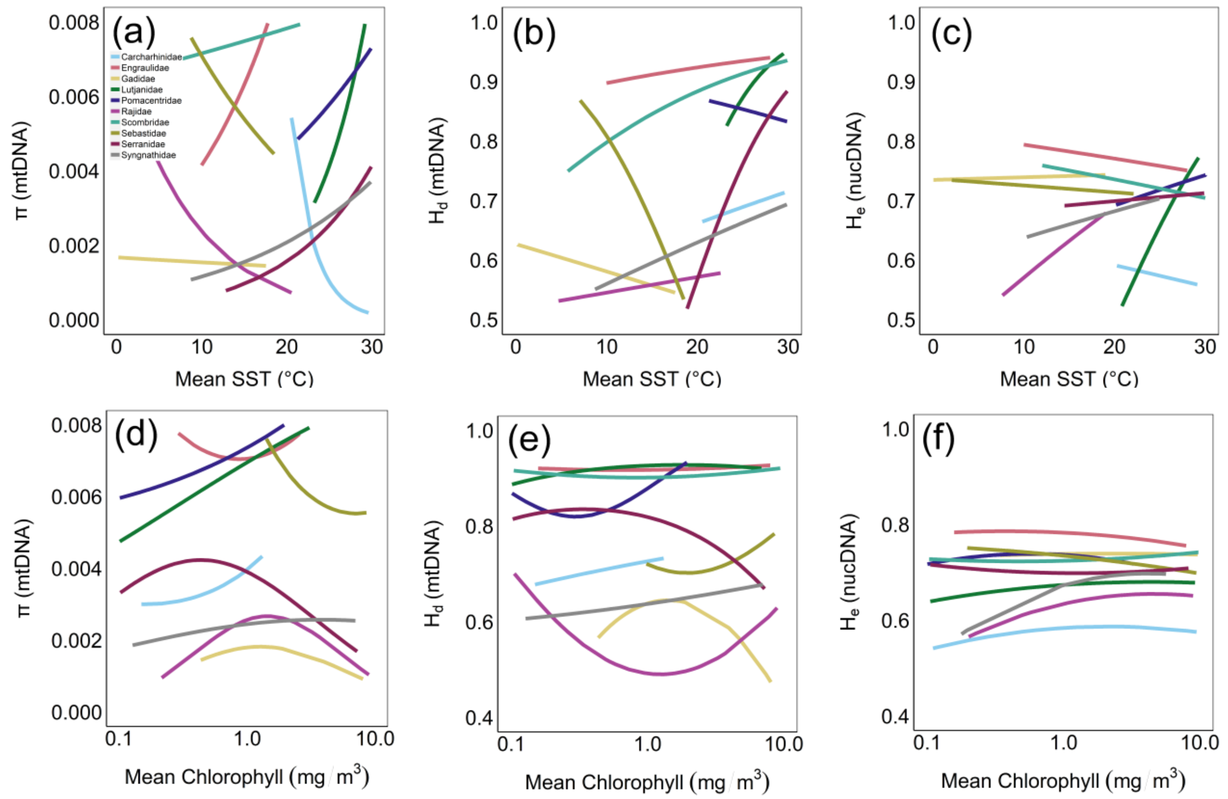


**Figure S2.13.** Relationship between mean SST (a-c), mean chlorophyll-a concentration (d-f) and genetic diversity for 10 families (a & d: mitochondrial π; b & e: mitochondrial $H_d$; c & f: nuclear microsatellite $H_e$). Mean chlorophyll-a concentration is plotted on a common logarithm scale.

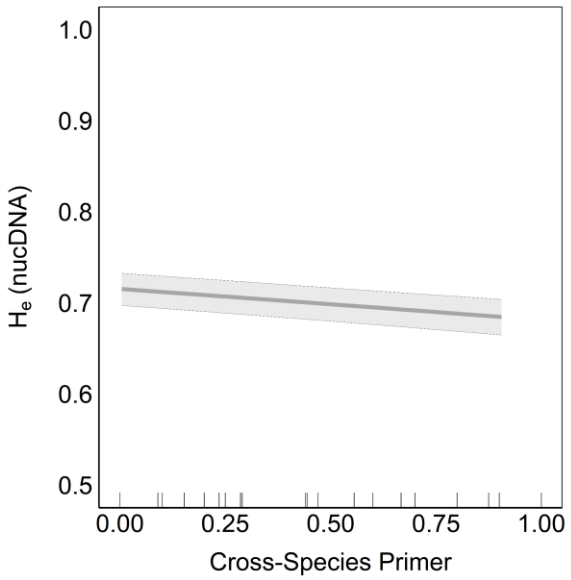


**Figure S2.14.** Relationship between cross-species primers (e.g. whether DNA was amplified with a primer developed in a different species) and nuclear $H_e$. Gray line represents the predicted relationship based on the mixed effects model (null) with shaded 95% confidence intervals. Rug plot on the x-axis illustrates the cross-species primer sampling extent (0 = same species, 1 = different species, values in-between indicate $H_e$ was averaged across multiple microsatellite markers and a fraction were developed cross-species).

**Table S2.1.** Moran's I for all mitochondrial DNA (π and $H_d$) and nuclear (microsatellite $H_e$) model residuals. 95% confidence intervals provided in brackets.

| **Model** | **π (mtDNA)** | **$H_d$ (mtDNA)** | **$H_e$ (mtDNA)** |
|---|---|---|---|
| **null** | 0.0041 [-0.0124, 0.0207] | -0.0055 [-0.0220, 0.0109] | 0.0013 [-0.0104, 0.0131] |
| **GEOGRAPHIC** | | | |
| **absolute latitude** | 0.0054 [-0.0112, 0.0219] | -0.0055 [-0.0220, 0.0109] | 0.0013 [-0.0104, 0.0131] |
| **latitude** | 0.0041 [-0.0125, 0.0206] | -0.0055 [-0.0220, 0.0109] | 0.0013 [-0.0104, 0.0131] |
| **longitude** | 0.0019 [-0.0147, 0.0184] | -0.0055 [-0.0220, 0.0109] | 0.0013 [-0.0104, 0.0131] |
| **absolute latitude & longitude** | 0.0018 [-0.0147, 0.0184] | -0.0055 [-0.0220, 0.0109] | 0.0013 [-0.0104, 0.0131] |
| **latitude & longitude** | 0.0004 [-0.0161, 0.0170] | -0.0055 [-0.0220, 0.0109] | 0.0013 [-0.0104, 0.0131] |
| **SEA SURFACE TEMPERATURE** | | | |
| **mean** | 0.0052 [-0.0113, 0.0218] | -0.0055 [-0.0220, 0.0109] | 0.0013 [-0.0104, 0.0131] |
| **CHLOROPHYLL-A CONCENTRATION** | | | |
| **mean** | 0.0044 [-0.0121, 0.0210] | -0.0055 [-0.0220, 0.0109] | 0.0013 [-0.0104, 0.0131] |
| **SEA SURFACE TEMPERATURE & CHLOROPHYLL-A CONCENTRATION** | | | |
| **mean(s)** | 0.0032 [-0.0133, 0.0198] | -0.0055 [-0.0220, 0.0109] | 0.0013 [-0.0104, 0.0131] |

p-value : * < 0.05
