# Supplementary material for: Global patterns of nuclear and mitochondrial genetic diversity in marine fishes
Source: Ecol Evol. 2024 May 6;14(5):e11365. doi: 10.1002/ece3.11365 (PMC11070773; doi:10.1002/ece3.11365)
Supplement: Supplementary file 3 — Appendix S3. [file ECE3-14-e11365-s003.pdf]

SUPPORTING INFORMATION: DATA SOURCES

TABLE S3.1. List of studies incorporated into both the nuclear (microsatellite) and mitochondrial DNA datasets. For mitochondrial DNA, all studies reported both H_d and π unless otherwise specified. References are provided after the table.

Family	Species	Common name	nuclear	mitochondrial	References
Pomacentridae	*Abudefduf saxatilis*	Seargeant-major	X	X	Piñeros *et al*. (2015)[a]; Piñeros *et al*. (2015)[b]; Piñeros & Gutiérrez-Rodriguez (2017)
Pomacentridae	*Acanthochromis polyacanthus*	Spiny chromis	X		Miller-Sims *et al*. (2005)[a]; Miller-Sims *et al*. (2008)
Scombridae	*Acanthocybium solandri*	Wahoo		X	Garber *et al*. (2005)
Sparidae	*Acanthopagrus australis*	Yellowfin bream	X		Roberts & Ayre (2010)
Sparidae	*Acanthopagrus berda*	Goldsilk seabream	X		Jean *et al*. (2006); Tseng *et al*. (2009)
Sparidae	*Acanthopagrus latus*	Yellowfin seabream	X		Ghasemi & Shadi (2018)
Sparidae	*Acanthopagrus schlegelii*	Blackhead seabream	X	X	An *et al*. (2010); Jean *et al*. (1998); Jeong *et al*. (2003); Kim *et al*. (2010)[a]; Liu *et al*. (2007)
Sparidae	*Acanthopagrus taiwanensis*	Taiwan picnic seabream	X		Tseng *et al*. (2009)
Acanthuridae	*Acanthurus leucosternon*	Powder blue surgeonfish	X		DiBattista *et al*. (2011)[a]; Otwoma *et al*. (2018)[a]
Acanthuridae	*Acanthurus nigricans*	Whitecheek surgeonfish	X		DiBattista *et al*. (2011)[a]
Acanthuridae	*Acanthurus nigrofuscus*	Brown surgeonfish		X	Eble *et al*. (2011)[a]

Acanthuridae	*Acanthurus nigroris*	Bluelined surgeonfish		X	DiBattista *et al*. (2011)[b]
Acanthuridae	*Acanthurus triostegus*	Convict surgeonfish	X	X	Otwoma *et al*. (2018)[b]; Otwoma & Reuter (2019)
Myliobatidae	*Aetobatus narinari*	Spotted eagle ray	X	X	Sellas *et al*. (2011); Sellas *et al*. (2015)
Alopiidae	*Alopias vulpinus*	Thresher		X	Vella *et al*. (2017)
Rajidae	*Amblyraja radiata*	Starry ray		X	Chevolot *et al*. (2007)
Ammodytidae	*Ammodytes personatus*	Pacific sandlance	X	X	Deng *et al*. (2019); Kim *et al*. (2006); Ren *et al*. (2015)
Pomacentridae	*Amphiprion akallopisos*	Skunk clownfish	X	X	Huyghe & Kochzius (2018); O'Donnell *et al*. (2017)
Pomacentridae	*Amphiprion clarkii*	Yellowtail clownfish	X		Pinsky *et al*. (2010)
Pomacentridae	*Amphiprion frenatus*	Tomato clownfish	X		Sato *et al*. (2014); Sato *et al*. (2017)
Pomacentridae	*Amphiprion latezonatus*	Wide-band anemonefish		X	Steinberg *et al*. (2016)
Pomacentridae	*Amphiprion mccullochi*	Whitesnout anemonefish	X		van der Meer *et al*. (2012)
Pomacentridae	*Amphiprion melanopus*	Fire clownfish	X	X (π)	Bonin *et al*. (2016); Drew *et al*. (2008)
Pomacentridae	*Amphiprion ocellaris*	Clown anemonefish	X		Madduppa *et al*. (2014); Timm *et al*. (2012); Timm *et al*. (2017)
Pomacentridae	*Amphiprion percula*	Orange clownfish	X		Bonin *et al*. (2016); Buston *et al*. (2007)
Pomacentridae	*Amphiprion perideraion*	Pink anemonefish	X		Donha *et al*. (2015); Madduppa *et al*. (2014); Sato *et al*. (2014); Sato *et al*. (2017)

Pomacentridae	*Amphiprion polymnus*	Saddleback clownfish	X		Saenz-Agudelo *et al*. (2012)
Anarhichadidae	*Anarhichas lupus*	Atlantic wolffish	X		McCusker & Bentzen (2010); Pampoulie *et al*. (2012)
Anoplopomatidae	*Anoplopoma fimbria*	Sablefish	X	X	Tripp-Valdez *et al*. (2012)
Moridae	*Antimora rostrata*	Blue antimoral	X		White *et al*. (2011)[a]
Trichiuridae	*Aphanopus carbo*	Black scabbardfish	X		Knutsen *et al*. (2009)
Trichiuridae	*Aphanopus intermedius*	Intermediate scabbardfish	X		Knutsen *et al*. (2009)
Gobiidae	*Aphia minuta*	Transparent goby	X		Ruggeri *et al*. (2016)[a]
Apogonidae	*Apogon imberbis*	Cardinal fish	X		Galarza *et al*. (2007)[a]; Galarza *et al*. (2009)[a]; Muths *et al*. (2015)
Sparidae	*Archosargus probatocephalus*	Sheepshead		X (π)	Seyoum *et al*. (2017)
Sciaenidae	*Argyrosomus coronus*	Dusky kob	X		Henriques *et al*. (2018)
Sciaenidae	*Argyrosomus inodorus*	Mild meagre	X		Henriques *et al*. (2015)
Sciaenidae	*Argyrosomus japonicus*	Japanese meagre	X		Barnes *et al*. (2015)
Sciaenidae	*Argyrosomus regius*	Meagre	X		Haffray *et al*. (2012); Porta *et al*. (2010)
Atherinopsidae	*Atherinella brasiliensis*	Brazilian silverside		X	da Silva Cortinhas *et al*. (2016)
Sciaenidae	*Atractoscion aequidens*	Geelbeck croaker	X		Henriques *et al*. (2012)[a]; Henriques *et al*. (2014)
Scombridae	*Auxis rochei*	Bullet tuna		X	Habib & Sulaiman (2016)

Scombridae	*Auxis thazard*	Frigate tuna		X	Habib & Sulaiman (2016); Kumar *et al*. (2012)[a]; Pedrosa-Gerasmio *et al*. (2015)
Balistidae	*Balistes capriscus*	Grey triggerfish		X	Antoni *et al*. (2011)
Gobiidae	*Bathygobius cocosensis*	Cocos frill-goby		X	Mukai *et al*. (2009)
Dasyatidae	*Bathytoshia centroura*	Roughtail stingray		X	Vella *et al*. (2009)
Rajidae	*Beringraja pulchra*	Mottled skate		X	Im *et al*. (2017)
Tripterygiidae	*Bellapiscis lesleyae*	Mottled twister		X	Hickey *et al*. (2009)
Tripterygiidae	*Bellapiscis medius*	Twister		X	Hickey *et al*. (2009)
Berycidae	*Beryx decadactylus*	Alfonsino		X	Friess & Sedberry (2011)
Berycidae	*Beryx splendens*	Splendid alfonsino		X	Lévy-Hartmann *et al*. (2011)
Gadidae	*Boreogadus saida*	Polar cod	X	X	Madsen *et al*. (2016); Pálsson *et al*. (2009); Wilson *et al*. (2019)
Malacanthidae	*Branchiostegus japonicus*	Horsehead tilefish		X	Nohara *et al*. (2009)[a]
Branchiostomati dae	*Branchiostoma belcheri*	NA		X	Li *et al*. (2013)[a]
Branchiostomati dae	*Branchiostoma japonicum*	NA		X	Li *et al*. (2013)[a]
Clupeidae	*Brevoortia gunteri*	Finescale menhaden	X		Anderson & Karel (2007); Anderson & McDonald (2007); Lynch (2008)
Clupeidae	*Brevoortia patronus*	Gulf menhaden	X		Anderson & Karel (2007); Anderson & McDonald (2007); Lynch (2010)
Clupeidae	*Brevoortia smithi*	Yellowfin menhaden	X		Anderson & Karel (2014); Lynch (2008)

Clupeidae	*Brevoortia tyrannus*	Atlantic menhaden	X		Anderson & Karel (2007); Lynch *et al*. (2010)
Lotidae	*Brosme brosme*	Cusk	X		Knutsen *et al*. (2007)
Gobiidae	*Caffrogobius caffer*	NA		X	Neethling *et al*. (2008)
Carcharhinidae	*Carcharhinus albimarginatus*	Silvertip shark	X		Green *et al*. (2019)
Carcharhinidae	*Carcharhinus amblyrhynchos*	Gray reef shark	X		Boissin *et al*. (2019)
Carcharhinidae	*Carcharhinus galapagensis*	Galapagos shark		X	Pazmiño *et al*. (2017)
Carcharhinidae	*Carcharhinus isodon*	Finetooth shark	X		Portnoy *et al*. (2016)
Carcharhinidae	*Carcharhinus leucas*	Bull shark	X		Pirog *et al*. (2019)
Carcharhinidae	*Carcharhinus limbatus*	Blacktip shark	X		Almojil *et al*. (2018); Keeney *et al*. (2005)
Carcharhinidae	*Carcharhinus signatus*	Night shark	X		Domingues *et al*. (2019)
Carcharhinidae	*Carcharhinus sorrah*	Spot-tail shark	X		Almojil *et al*. (2018)
Carcharhinidae	*Carcharias taurus*	Sand tiger shark	X		Feldheim *et al*. (2007)
Lamnidae	*Carcharodon carcharias*	Great white shark	X	X	Andreotti *et al*. (2016); Blower *et al*. (2012); Pardini *et al*. (2000)
Centrophoridae	*Centrophorus harrissoni*	Dumb gulper shark		X (π)	Daley *et al*. (2012)
Centrophoridae	*Centrophorus moluccensis*	Smallfin gulper shark		X (π)	Daley *et al*. (2012)
Centrophoridae	*Centrophorus uyato*	Little gulper shark		X	Vella *et al*. (2017)
Centrophoridae	*Centrophorus zeehaani*	Southern dogfish		X (π)	Daley *et al*. (2012)

Serranidae	*Centropristis striata*	Black seabass		X	Roy *et al*. (2012)
Somniosidae	*Centroscymnus crepidater*	Longnose velvet dogfish	X	X	Cunha *et al*. (2012); Helyar *et al*. (2011)
Serranidae	*Cephalopholis argus*	Peacock hind		X	Gaither *et al*. (2011)[a]; Gaither *et al*. (2012)
Serranidae	*Cephalopholis fulva*	Coney	X	X	De Souza *et al*. (2015); Renshaw *et al*. (2010)
Engraulidae	*Cetengraulis edentulous*	Atlantic anchoveta		X (H_d)	Grant *et al*. (2010)
Chaetodontidae	*Chaenocephalus aceratus*	Blackfin icefish	X		Damerau *et al*. (2012); Damerau *et al*. (2014)[a]; Papetti *et al*. (2007); Susana *et al*. (2007)
Chaetodontidae	*Chaetodon austriacus*	Blacktail butterflyfish		X	Waldrop *et al*. (2016)
Chaetodontidae	*Chaetodon lunulatus*	Oval butterflyfish	X	X	Lawton *et al*. (2010); Lawton *et al*. (2011); Montanari *et al*. (2011); Waldrop *et al*. (2016)
Chaetodontidae	*Chaetodon melapterus*	Arabian butterflyfish		X	Waldrop *et al*. (2016)
Chaetodontidae	*Chaetodon meyeri*	Scrawled butterflyfish		X	DiBattista *et al*. (2012)
Chaetodontidae	*Chaetodon multicinctus*	Pebbled butterflyfish	X		Heist *et al*. (2008)
Chaetodontidae	*Chaetodon ornatissimus*	Ornate butterflyfish		X	DiBattista *et al*. (2012)
Chaetodontidae	*Chaetodon tricinctus*	Three-striped butterflyfish	X	X	van der Meer *et al*. (2013)[a]
Chaetodontidae	*Chaetodon trifasciatus*	Melon butterflyfish	X	X	Lawton *et al*. (2010); Lawton *et al*. (2011); Montanari *et al*. (2011); Waldrop *et al*. (2016)

Channichthyidae	*Champsocephalus gunnari*	Mackerel icefish	X		Damerau *et al*. (2012); Damerau *et al*. (2014)[a]; Young *et al*. (2015)
Labridae	*Cheilinus undulatus*	Humphead wrasse	X		Hu *et al*. (2013)
Apogonidae	*Cheilodipterus artus*	Wolf cardinalfish	X		Underwood (2010)
Channichthyidae	*Chionodraco rastrospinosus*	Ocellated icefish	X		Damerau *et al*. (2012); Papetti *et al*. (2012)
Pomacentridae	*Chromis margaritifer*	Bicolor chromis	X		Underwood (2009); Underwood *et al*. (2012)
Pomacentridae	*Chromis multilineata*	Brown chromis		X	Rocha *et al*. (2008)
Pomacentridae	*Chromis viridis*	Blue green damselfish		X	Liu *et al*. (2019)[a]
Pomacentridae	*Chrysiptera talboti*	Talbot's demoiselle		X (π)	Drew *et al*. (2008)
Sparidae	*Chrysoblephus laticeps*	Roman seabream	X	X	Teske *et al*. (2009); Teske *et al*. (2010)
Sparidae	*Chrysoblephus puniceus*	Slinger seabream	X	X	Chopelet *et al*. (2009); Duncan *et al*. (2015)
Labridae	*Cirrhilabrus punctatus*	Dotted wrasse		X (π)	Drew *et al*. (2008)
Pleuronectidae	*Cleisthenes herzensteini*	Sôhachi		X	Xiao *et al*. (2011)
Clupeidae	*Clupea harengus*	Atlantic herring	X		Bekkevold *et al*. (2005); Bekkevold *et al*. (2016); Jørgensen *et al*. (2005)[a]; Jørgensen *et al*. (2005)[b]; Larsson *et al*. (2010); Mariani *et al*. (2005); McPherson *et al*. (2001)[a]; McPherson *et al*. (2001)[b]; Pampoulie *et al*. (2015); Shaw *et al*. (1999); Teacher *et al*. (2013); Wennerström *et al*. (2013)

Clupeidae	*Clupea pallasii pallassii*	Pacific herring	X	X	Beacham *et al*. (2001); Grant *et al*. (2012); Mitchell (2006); O'Connell *et al*. (1998)[a]; O'Connell *et al*. (1998)[b]; Olsen *et al*. (2002); Semenova *et al*. (2015); Semenova *et al*. (2018); Small *et al*. (2005); Wildes *et al*. (2011)
Sciaenidae	*Collichthys lucidus*	Spinyhead croaker		X	Xiao *et al*. (2011)
Congridae	*Conger conger*	European conger		X	Correia *et al*. (2006); Correia *et al*. (2012)
Labridae	*Coris bulbifrons*	Doubleheader	X	X	van der Meer *et al*. (2013)[a]; van der Meer *et al*. (2015)
Labridae	*Coris julis*	Mediterranean rainbow wrasse	X		Guillemaud *et al*. (2000)
Coryphaenidae	*Coryphaena hippurus*	Common dolphinfish	X		Tripp-Valdez *et al*. (2010)
Macrouridae	*Coryphaenoides armatus*	Abyssal grenadier	X		Ritchie *et al*. (2013)
Macrouridae	*Coryphaenoides brevibarbis*	Shortbeard grenadier	X		White *et al*. (2011)[b]
Macrouridae	*Coryphaenoides mediterraneus*	Mediterranean grenadier	X	X	Catarino *et al*. (2017)
Liparidae	*Crystallias matsushimae*	NA		X	Tohkairin *et al*. (2016)
Labridae	*Ctenolabrus rupestris*	Goldsinny-wrasse	X		Jansson *et al*. (2017)
Cyclopteridae	*Cyclopterus lumpus*	Lumpfish	X		Jónsdóttir *et al*. (2018); Pampoulie *et al*. (2014)
Embiotocidae	*Cymatogaster aggregata*	Shiner perch	X		Liu & Avise (2011)
Cynoglossidae	*Cynoglossus semilaevis*	Tongue sole	X		Liu *et al*. (2008); Liu *et al*. (2011)

Sciaenidae	*Cynoscion arenarius*	Sand weakfish	X		Anderson *et al*. (2009)
Sciaenidae	*Cynoscion nebulosus*	Spotted weakfish	X	X	Anderson & Karel (2009); Anderson & Karel (2010); Renshaw *et al*. (2009)[a]
Sciaenidae	*Cynoscion nothus*	Silver seatrout	X		Anderson *et al*. (2009)
Dalatiidae	*Dalatias licha*	Kitefin shark		X	Vella *et al*. (2017)
Pomacentridae	*Dascyllus aruanus*	Humbug damselfish	X		Liu *et al*. (2014)[a]; Pini *et al*. (2011)
Pomacentridae	*Dascyllus strasburgi*	Strasburg's dascyllus	X		Leray *et al*. (2010)
Pomacentridae	*Dascyllus trimaculatus*	Threespot dascyllus	X		Bernardi *et al*. (2012); Leray *et al*. (2010)
Dasyatidae	*Dasyatis pastinaca*	Common stingray		X	Vella *et al*. (2017)
Sparidae	*Dentex dentex*	Common dentex	X		Viret *et al*. (2018)
Myctophidae	*Diaphus theta*	California headlightfish		X	Kojima *et al*. (2009)
Moronidae	*Dicentrarchus labrax*	European seabass	X	X	Bahri-Sfar *et al*. (2000);Fritsch *et al*. (2007); García de León *et al*. (1997); Lemaire *et al*. (2005); Naciri *et al*. (1999)
Sparidae	*Diplodus hottentotus*	Zebra seabream	X		Gwilliam *et al*. (2018)
Sparidae	*Diplodus sargus*	White seabream	X	X	Di Franco *et al*. (2012); Exadactylos *et al*. (2019); González-Wangüemert *et al*. (2010); González-Wangümert *et al*. (2011); González-Wangüemert & Pérez-Ruzafa (2012); González-Wangüemert *et al*. (2012); Kaouèche *et al*. (2013); Pujolar *et al*. (2013)
Sparidae	*Diplodus vulgaris*	Common two-banded	X		Galarza *et al*. (2009)[a]; Roques *et al*.

		seabream			(2007); Steffani *et al*. (2015); Sahyoun *et al*. (2016)
Rajidae	*Dipturus oxyrinchus*	Longnosed skate		X	Vella *et al*. (2017)
Rajidae	*Dipturus trachyderma*	Roughskin skate	X	X	Vargas-Caro *et al*. (2017)
Nototheniidae	*Dissostichus eleginoides*	Patagonian toothfish	X		Appleyard *et al*. (2004); Arenada *et al*. (2017); Garcia *et al*. (2019); Reilly & Ward (1999); Rogers *et al*. (2006); Shaw *et al*. (2004); Smith & McVeagh (2000)
Myctophidae	*Electrona antarctica*	Antarctica lanternfish	X		Valenzuela-Quiñonez (2014); Van de Putte *et al*. (2012)[a]
Eleginopsidae	*Eleginops maclovinus*	Patagonian blennie	X	X	Ceballos *et al*. (2012); Ceballos *et al*. (2016)
Elopidae	*Elops saurus*	Ladyfish	X		Sha *et al*. (2009)
Embiotocidae	*Embiotoca jacksoni*	Black perch	X		Bernardi (2008)
Engraulidae	*Engraulis australis*	Australian anchovy	X	X	Silva *et al*. (2017)
Engraulidae	*Engraulis capensis*	Southern African anchovy	X	X	Silva *et al*. (2017)
Engraulidae	*Engraulis encrascicolus*	European anchovy	X	X	Borrell *et al*. (2012); Ouazzani *et al*. (2017); Pakaki *et al*. (2009); Ruggeri *et al*. (2016)[b]; Silva *et al*. (2014); Viñas *et al*. (2014)
Engraulidae	*Engraulis eurystole*	Silver anchovy	X	X	Silva *et al*. (2014)
Engraulidae	*Engraulis japonicus*	Japanese anchovy	X	X	Chen *et al*. (2010); Lin *et al*. (2011); Liu *et al*. (2006); Silva *et al*. (2017); Yu *et al*. (2002); Yu *et al*. (2005); Zheng *et al*.

					(2015)
Serranidae	*Epinephelus akaara*	Hong Kong grouper	X	X	Chen *et al*. (2008); Koedprang *et al*. (2007)
Serranidae	*Epinephelus awoara*	Yellow grouper	X		Zhao *et al*. (2009)[a]
Serranidae	*Epinephelus bleekeri*	Duskytail grouper	X		Koedprang *et al*. (2007)
Serranidae	*Epinephelus bruneus*	Kelp grouper	X		An *et al*. (2012)[a]; Kang *et al*. (2013)
Serranidae	*Epinephelus coioides*	Orange-spotted grouper	X		Antoro *et al*. (2006); Koedprang *et al*. (2007); Pumitinsee *et al*. (2009); Wang *et al*. (2010)[a]
Serranidae	*Epinephelus fasciatus*	Blacktip grouper		X (H_d)	Kuriiwa *et al*. (2014)
Serranidae	*Epinephelus guttatus*	Red hind	X		Ramírez *et al*. (2006); Renshaw *et al*. (2010)
Serranidae	*Epinephelus itajara*	Atlantic goliath grouper		X	Craig *et al*. (2009)
Serranidae	*Epinephelus lanceolatus*	Giant grouper	X		Wang *et al*. (2016); Yang *et al*. (2011)
Serranidae	*Epinephelus maculatus*	Highfin grouper	X		Koedprang *et al*. (2007)
Serranidae	*Epinephelus malabaricus*	Malabar grouper	X		Koedprang *et al*. (2007)
Serranidae	*Epinephelus marginatus*	Dusky grouper	X		Buchholz-Sørensen & Vella (2016); de Innocentiis *et al*. (2001); Schunter *et al*. (2011)[a]
Serranidae	*Epinephelus merra*	Honeycomb grouper	X	X	Koedprang *et al*. (2007); Matias *et al*. (2013); Muths & Bourjea (2011)
Serranidae	*Epinephelus morio*	Red grouper	X		Zatcoff *et al*. (2004)

Serranidae	*Epinephelus ongus*	White-streaked grouper	X		Koedprang *et al*. (2007)
Serranidae	*Epinephelus polyphekadion*	Camouflage grouper	X		Ma *et al*. (2018); Rhodes *et al*. (2003)
Serranidae	*Epinephelus striatus*	Nassau grouper	X	X	Jackson *et al*. (2014); Sherman *et al*. (2017)
Lutjanidae	*Etelis carbunculus*	Deep-water red snapper		X	Andrews *et al*. (2014)
Lutjanidae	*Etelis coruscans*	Deep-water longtail red snapper		X	Andrews *et al*. (2014)
Etmopteridae	*Etmopterus molleri*	Slendertail lanternshark	X		Oury *et al*. (2019)
Etmopteridae	*Etmopterus spinax*	Velvet belly	X		Oury *et al*. (2019)
Gobiidae	*Eucyclogobius newberryi*	Tidewater goby	X		McCraney *et al*. (2010)
Scombridae	*Euthynnus affinis*	Kawakawa		X	Kumar *et al*. (2012)[b]; Santos *et al*. (2010)
Gobiidae	*Eviota albolineata*	White-line dwarfgoby		X	Farnsworth *et al*. (2010)
Gobiidae	*Eviota queenslandica*	Queensland dwarfgoby		X	Farnsworth *et al*. (2010)
Fistulariidae	*Fistularia commersonii*	Bluespotted cornetfish		X (H_d)	Golani *et al*. (2007)
Tripterygiidae	*Forsterygion capito*	Spotted robust triplefin	X	X	Hickey *et al*. (2009); Rabone *et al*. (2015)
Tripterygiidae	*Forsterygion gymnotum*	Tasmanian robust triplefin		X	Hickey *et al*. (2009)

Tripterygiidae	*Forsterygion lapillum*	Common triplefin	X	X	Hickey *et al*. (2009); Rabone *et al*. (2015)
Tripterygiidae	*Forsterygion nigripenne*	Estuarine triplefin		X	Hickey *et al*. (2009)
Tripterygiidae	*Forsterygion varium*	Striped triplefin		X	Hickey *et al*. (2009)
Gadidae	*Gadus chalcogrammus*	Alaska pollock	X		O'Reilly *et al*. (2004); Olsen *et al*. (2002); Shubina *et al*. (2009)
Gadidae	*Gadus macrocephalus*	Pacific cod	X	X	Canino *et al*. (2005); Canino *et al*. (2010); Cunningham (2007); Cunningham *et al*. (2009); Gwak & Nakayama (2011); Kim *et al*. (2010)[b]; Liu *et al*. (2010)[a]; Song *et al*. (2016); Spies (2012); Stroganov *et al*. (2009)
Gadidae	*Gadus morhua*	Atlantic cod	X	X	Bentzen *et al*. (1996); Bradbury *et al*. (2009); Carr *et al*. (1995); Dahle *et al*. (2018); Karlsson & Monk (2005); Kijewska *et al*. (2011); Knutsen *et al*. (2003); Lait *et al*. (2018); Nielsen *et al*. (2003); O'Leary *et al*. (2007); Pampoulie *et al*. (2006); Poulsen *et al*. (2006); Ruzzante *et al*. (1996)[a]; Ruzzante *et al*. (1996)[b]; Ruzzante *et al*. (1997); Ruzzante *et al*. (1998); Ruzzante *et al*. (2000); Ruzzante *et al*. (2001); Skarstein *et al*. (2007); Wennevik *et al*. (2008)
Triakidae	*Galeorhinus galeus*	School shark	X		Bester-van der Merwe *et al*. (2017); Maduna *et al*. (2017)
Scyliorhinidae	*Galeus melastomus*	Blackmouth catshark		X (H_d)	Ferrari *et al*. (2018)
Ophidiidae	*Genypterus capensis*	Kingklip	X	X	Henriques *et al*. (2017)
Ginglymostomati	*Ginglymostoma cirratum*	Nurse shark	X	X	Karl *et al*. (2012); Heist *et al*. (2003)

dae					
Girellidae	*Girella laevifrons*	NA	X		Cerda *et al*. (2019)
Girellidae	*Girella punctata*	Largescale blackfish	X	X	Saito *et al*. (2008); Umino *et al*. (2009)
Glaucosomatidae	*Glaucosoma hebraicum*	West Australian dhufish	X		Berry *et al*. (2012)[a]; Burridge & England (2009)
Pleuronectidae	*Glyptocephalus stelleri*	Blackfin flounder		X	Xiao *et al*. (2010)
Gobiidae	*Gnatholepis anjerensis*	Eye-bar goby	X		Thacker *et al*. (2008)
Gobiidae	*Gnatholepis cauerensis*	Eyebar goby	X		Thacker *et al*. (2008)
Nototheniidae	*Gobionotothen gibberifrons*	Humped rockcod	X		Damerau *et al*. (2012); Matschiner *et al*. (2009)
Scombridae	*Gymnosarda unicolor*	Dogtooth tuna	X		Bentley *et al*. (2014)
Muraenidae	*Gymnothorax chilospilus*	Lipspot moray	X		Ribout *et al*. (2018)
Haemulidae	*Haemulon flavolineatum*	French grunt	X		Purcell *et al*. (2006); Williams *et al*. (2004)[a]
Haemulidae	*Haemulon plumierii*	White grunt	X		O'Donnell *et al*. (2019)
Labridae	*Halichoeres cyanocephalus*	Yellowcheek wrasse		X	Rocha (2004)
Labridae	*Halichoeres garnoti*	Yellowhead wrasse		X	Rocha (2004)
Labridae	*Halichoeres maculipinna*	Clown wrasse		X	Rocha (2004)
Hapalogenyidae	*Hapalogenys nigripinnis*	Short barbeled velvetchin	X		An *et al*. (2014)
Scorpaenidae	*Helicolenus dactylopterus*	Blackbelly rosefish	X		Aboim *et al*. (2003)

Hexanchidae	*Heptranchias perlo*	Sharpnose sevengill shark		X	Vella *et al*. (2017)
Hexagrammidae	*Hexagrammos agrammus*	Spotty-bellied greenling		X	Habib *et al*. (2015)
Hexagrammidae	*Hexagrammos otakii*	Fat greenling		X	Habib *et al*. (2011); Ren *et al*. (2013)
Hexanchidae	*Hexanchus griseus*	Bluntnose sixgill shark	X	X	Larson *et al*. (2011); Vella *et al*. (2017)
Syngnathidae	*Hippocampus angustus*	Western spiny seahorse	X		Jones *et al*. (1998)[a]
Syngnathidae	*Hippocampus guttulatus*	Long-snouted seahorse	X		Pardo *et al*. (2007); Woodall *et al*. (2015)
Syngnathidae	*Hippocampus hippocampus*	Short-snouted seahorse	X	X	Lopez *et al*. (2010)[a]; Woodall *et al*. (2011)
Syngnathidae	*Hippocampus ingens*	Pacific seahorse		X	Saarman *et al*. (2010)
Syngnathidae	*Hippocampus kuda*	Spotted seahorse		X	Goswami *et al*. (2009); Panithanarak *et al*. (2010); Teske *et al*. (2005)
Syngnathidae	*Hippocampus mohnikei*	Japanese seahorse		X	Zhang *et al*. (2014)[a]
Syngnathidae	*Hippocampus trimaculatus*	Longnose seahorse		X	Goswami *et al*. (2009); Zhang *et al*. (2014)[a]
Pleuronectidae	*Hippoglossus hippoglossus*	Atlantic halibut	X		Galindo *et al*. (2011); McGowan & Reith (1999)
Pleuronectidae	*Hippoglossus stenolepis*	Pacific halibut	X	X	Drinan *et al*. (2016); Galindo *et al*. (2011); Hauser *et al*. (2006); Nielsen *et al*. (2010)
Holocentridae	*Holocentrus adscensionis*	Squirrelfish		X	Bowen *et al*. (2006)

Trachichthyidae	*Hoplostethus atlanticus*	Orange roughy	X	X	Carlsson *et al*. (2011); Coughlan *et al*. (2010); Varela *et al*. (2012); Varela *et al*. (2013); White *et al*. (2009)
Centrolophidae	*Hyperoglyphe antarctica*	Bluenose warehou		X	Robinson *et al*. (2008)
Serranidae	*Hypoplectrus nigricans*	Black hamlet	X		Puebla *et al*. (2008)
Serranidae	*Hypoplectrus puella*	Barred hamlet	X		Puebla *et al*. (2008); Puebla *et al*. (2009)
Serranidae	*Hyporthodus acanthistius*	Rooster hind	X		Beldade *et al*. (2009); Beldade *et al*. (2014)
Serranidae	*Hyporthodus septemfasciatus*	Convict grouper	X		Zhao *et al*. (2009)[b]
Lamnidae	*Isurus oxyrinchus*	Shortfin mako		X	Vella *et al*. (2017)
Istiophoridae	*Kajikia albida*	Atlantic white martin	X		Graves & McDowell (2006); Mamoozadeh *et al*. (2018)
Pleuronectidae	*Kareius bicoloratus*	Stone flounder	X		Kim *et al*. (2009)[a]; Tian *et al*. (2009)
Scombridae	*Katsuwonus pelamis*	Skipjack tuna	X	X	Dammannagoda (2007); Dammannagoda *et al*. (2011); Menezes *et al*. (2008); Menezes *et al*. (2012)
Labridae	*Labroides dimadiatus*	Bluestreak cleaner wrasse		X	Drew *et al*. (2008); Sims *et al*. (2014)
Labridae	*Larabicus quadrilineatus*	Fourline wrasse		X	Froukh & Kochzius (2007)
Sciaenidae	*Larimichthys crocea*	Large yellow croaker	X		Guo *et al*. (2005); Le Wang *et al*. (2012); Lü *et al*. (2013)
Sciaenidae	*Larimichthys polyactis*	Yellow croaker	X	X	Chen *et al*. (2009); Chen & Cheng (2013); Li *et al*. (2013)[b]; Liu *et al*. (2014)[b]; Liu *et*

					al. (2016); Ma *et al*. (2011)[a]; Wang *et al*. (2010)[b]; Wang *et al*. (2013); Wang *et al*. (2015); Xiao *et al*. (2009); Xiao *et al*. (2015)
Gobiesocidae	*Lepadogaster lepadogaster*	Shore clingfish	X		Klein *et al*. (2016)
Nototheniidae	*Lepidonotothen squamifrons*	Grey rockcod	X		Damerau *et al*. (2012)
Scophthalmidae	*Lepidorhombus boscii*	Four-spotted megrim	X	X	Campo & Garcia-Vazquez (2010); Danancher & Garcia-Vazquez (2009)
Scophthalmidae	*Lepidorhombus whiffiagonis*	Megrim	X		Danancher & Garcia-Vazquez (2009)
Lethrinidae	*Lethrinus harak*	Thumbprint emperor	X	X	Healey *et al*. (2018)[a]
Lethrinidae	*Lethrinus laticaudis*	Grass emperor	X		Barton *et al*. (2018)
Lethrinidae	*Lethrinus mahsena*	Sky emperor	X	X	Healey *et al*. (2018)[a]
Lethrinidae	*Lethrinus miniatus*	Trumpet emperor	X		Van Herwerden *et al*. (2003)
Lethrinidae	*Lethrunus nebulosus*	Spangled emperor	X	X	Berry *et al*. (2012)[b]; Healey *et al*. (*et al*. (2018)[b]
Atherinopsidae	*Leuresthes tenuis*	California grunion		X	Johnson *et al*. (2009)
Carangidae	*Lichia amia*	Leerfish		X	Henriques *et al*. (2012)[b]
Pleuronectidae	*Limanda limanda*	Common dab	X		Tysklind *et al*. (2009); Tysklind *et al*. (2013)
Pleuornectidae	*Limanda punctatissima*	Speckled flounder	X		Kim *et al*. (2009)[a]

Blenniidae	*Lipophrys pholis*	Shanny		X	Francisco *et al*. (2006); Francisco *et al*. (2011)
Sparidae	*Lithognathus mormyrus*	Sand steenbras	X	X	Sala-Bozano *et al*. (2009)[a]; Sala-Bozano *et al*. (2009)[b]
Lophiidae	*Lophius budegassa*	Blackbellied angler	X		Blanco *et al*. (2006)
Lophiidae	*Lophius piscatorius*	Angler	X		Blanco *et al*. (2006); Chevolot *et al*. (2006)[a]
Lutjanidae	*Lutjanus analis*	Mutton snapper	X	X	Carson *et al*. (2011); De Souza *et al*. (2019); Renshaw *et al*. (2007); Shulzitski (2005)
Lutjanidae	*Lutjanus campechanus*	Northern red snapper	X		Hollenbeck *et al*. (2015); Saillant (2003); Saillant & Gold (2006); Saillant *et al*. (2006); Saillant *et al*. (2010)
Lutjanidae	*Lutjanus carponotatus*	Spanish flag snapper	X	X	Evans *et al*. (2010); Harrison *et al*. (2014); Veilleux *et al*. (2011)
Lutjanidae	*Lutjanus fulvus*	Blacktail snapper		X	Gaither *et al*. (2010)[a]; Gaither *et al*. (2012); Harrison *et al*. (2014)
Lutjanidae	*Lutjanus jocu*	Dog snapper		X	De Souza *et al*. (2019)
Lutjanidae	*Lutjanus kasmira*	Common bluestripe snapper		X	Gaither *et al*. (2010)[a]; Gaither *et al*. (2010)[b]; Gaither *et al*. (20120
Lutjanidae	*Lutjanus peru*	Pacific red snapper	X		Munguía-Vega *et al*. (2018); Paz-García *et al*. (2017)
Lutjanidae	*Lutjanus purpureus*	Southern red snapper		X	Gomes *et al*. (2008)
Lutjanidae	*Lutjanus russellii*	Russell’s snapper	X		Guo *et al*. (200)

Lutjanidae	*Lutjanus synagris*	Lane snapper	X	X	Gold 2011*et al*. (); Karlsson *et al*. (2009); Renshaw *et al*. (2007); Silva *et al*. (2018)
Sciaenidae	*Macrodon ancylodon*	King weakfish		X	Santos *et al*. (2006)
Macrouridae	*Macrourus berglax*	Roughhead grenadier	X		Coscia *et al*. (2018); Helyar *et al*. (2010)
Macruronidae	*Macruronus margellanicus*	Patagonian grenadier	X		D'Amato *et al*. (1999); D'Amato (2006); Machado-Schiaffino & Garcia-Vazquez (2011)
Istiophoridae	*Makaira nigricans*	Blue marlin	X	X	McDowell *et al*. (2007); Sorenson *et al*. (2011)
Carangidae	*Megalaspis cordyla*	Torpedo scad	X		Kempter *et al*. (2017)
Gadidae	*Melanogrammus aeglefinus*	Haddock	X		Lage & Kornfield (1999); O'Reilly *et al*. (2002)
Atherinopsidae	*Menidia menidia*	Atlantic silverside	X	X	Mach *et al*. (2011); Sbrocco *et al*. (2011)
Sciaenidae	*Menticirrhus americanus*	Southern kingcroaker		X	Freitas *et al*. (2017)
Gadidae	*Merlangius merlangus*	Whiting	X	X	Charrier *et al*. (2007); Eiríksson & Árnason (2014)
Merlucciidae	*Merluccius albidus*	Offshore silver hake		X	Machado-Schiaffino *et al*. (2010)
Merlucciidae	*Merluccius bilinearis*	Silver hake	X	X	Machado-Schiaffino *et al*. (2010); Machado-Schiaffino *et al*. (2011)
Merlucciidae	*Merluccius capensis*	Shallow-water cape hake		X	von der Heyden *et al*. (2007)
Merlucciidae	*Merluccius hubbsi*	Argentine hake	X		Machado-Schiaffino *et al*. (2011)
Merlucciidae	*Merluccius merluccius*	European hake	X		Castillo *et al*. (2005); Lundy *et al*. (2000);

					Pita *et al*. (2011); Pita *et al*. (2016); Tanner *et al*. (2014)
Merlucciidae	*Merluccius paradoxus*	Deep-water cape hake		X	von der Heyden *et al*. (2007)
Merlucciidae	*Merluccius productus*	North Pacific hake	X		García-De León *et al*. (2018)
Soleidae	*Microchirus azevia*	Bastard sole	X		Catanese *et al*. (2008)
Gadidae	*Micromesistius poutassou*	Blue whiting	X		Was *et al*. (2008)
Sciaenidae	*Micropogonias furnieri*	Whitemouth croaker	X	X	D'Anatro *et al*. (2011); Periera *et al*. (2009)
Sciaenidae	*Micropogonias undulatus*	Atlantic croaker		X	Anderson *et al*. (2019)
Pleuronectidae	*Microstomus pacificus*	Dover sole		X	Stepien (1999)
Sciaenidae	*Miichthys miiuy*	Mi-iuy croaker	X	X	Cheng *et al*. (2011); Wang *et al*. (2010)[c]; Xu *et al*. (2014); Zhao *et al*. (2009)[c]
Lotidae	*Molva molva*	Ling	X		Gonzalez *et al*. (2015); Ring *et al*. (2009)
Mullidae	*Mulloidichthys flavolineatus*	Yellowstripe goatfish	X	X	Fernandez-Silva *et al*. (2015)
Mullidae	*Mullus barbatus barbatus*	Red mullet	X		Félix-Hackradt *et al*. (2013); Galarza *et al*. (2007)[b]; Galarza *et al*. (2009)[b]; Garoia *et al*. (2004); Maggio *et al*. (2009); Matić-Skoko *et al*. (2018)
Mullidae	*Mullus surmuletus*	Surmullet	X		Félix-Hackradt *et al*. (2013); Galarza *et al*. (2007)[b]; Galarza *et al*. (2009)[a]; Galarza *et al*. (2009)[b]; Matić-Skoko *et al*. (2018)
Triakidae	*Mustelus asterias*	Starry smooth-hound		X	Vella *et al*. (2017)

Triakidae	*Mustelus mustelus*	Smooth-hound	X	X	Hull *et al*. (2019); Maduna *et al*. (2016); Vella *et al*. (2017)
Triakidae	*Mustelus palumbes*	Whitespotted smooth-hound	X		Maduna *et al*. (2017)
Serranidae	*Mycteroperca microlepis*	Gag	X		Cushman *et al*. (2009); Jue (2010)
Serranidae	*Mycteroperca phenax*	Scamp	X		Zatcoff *et al*. (2004)
Holocentridae	*Myripristis berndti*	Blotcheye soldierfish		X	Craig *et al*. (2007); Muths *et al*. (2011)
Holocentridae	*Myripristis jacobus*	Blackbar soldierfish		X	Bowen *et al*. (2006)
Acanthuridae	*Naso unicornis*	Bluespine unicornfish	X	X	Horne *et al*. (2010); Horne *et al*. (2013)
Cheilodactylidae	*Nemadactylus macropterus*	Tarakihi	X		Burridge & Smolenski (2003)
Pomacentridae	*Neopomacentrus filamentosus*	Brown demoiselle	X		Jones & Barber (2005)
Sciaenidae	*Nibea albiflora*	Yellow drum	X		Ma *et al*. (2011)[b]; Xing *et al*. (2009)[a]; Xu *et al*. (2017)
Nototheniidae	*Notothenia rossii*	Marbled rockcod	X		Young *et al*. (2015)
Nototheniidae	*Nototheniops larseni*	Painted notie	X		Damerau *et al*. (2014)[b]
Leiognathidae	*Nuchequula mannusella*	NA		X	Gao *et al*. (2019)[a]
Sparidae	*Oblada melanura*	Saddled seabream	X		Calò *et al*. (2016); Galarza *et al*. (2009)[a]
Lutjanidae	*Ocyurus chrysurus*	Yellowtail snapper	X		Renshaw *et al*. (2007)
Balistidae	*Odonus niger*	Red-toothed triggerfish		X	Matias *et al*. (2013)

Rajidae	*Okamejei kenojei*	Ocellate spot skate		X	Misawa *et al*. (2018)
Hexagrammidae	*Ophiodon elongatus*	Lingcod	X	X (π)	LeClair *et al*. (2006); Markos *et al*. (2007); Withler *et al*. (2003)
Opistognathidae	*Opistognathus aurifrons*	Yellowhead jawfish		X	Ho *et al*. (2012)
Oplegnathidae	*Oplegnathus fasciatus*	Barred knifefish		X	Xiao *et al*. (2016)
Apogonidae	*Ostorhinchus doederleini*	Doederlein's cardinalfish	X		Miller-Sims *et al*. (2004)
Sparidae	*Pagellus bogaraveo*	Blackspot seabream	X		Stockley *et al*. (2005)
Sparidae	*Pagellus erythrinus*	Common pandora		X	Angiulli *et al*. (2016)
Nototheniidae	*Pagothenia borchgrevinki*	Bald notothen	X		Van de Putte *et al*. (2009)
Sparidae	*Pagrus auratus*	Silver seabream	X		Bernal-Ramírez *et al*. (2003); Gardner *et al*. (2017); Hauser *et al*. (2002); Le Port *et al*. (2017)
Sparidae	*Pagrus auriga*	Redbanded seabream	X		Ponce *et al*. (2006)
Sparidae	*Pagrus major*	Red seabream	X	X	Hamasaki *et al*. (2010); Perez-Enriques & Taniguchi (1999); Shishidou *et al*. (2008)
Sparidae	*Pagrus pagrus*	Red porgy	X	X	Ball *et al*. (2007)
Stromateidae	*Pampus argenteus*	Silver pomfret	X	X	Peng *et al*. (2009); Sun *et al*. (2019)
Stromateidae	*Pampus chinensis*	Chinese silver pomfret	X	X	Li *et al*. (2019); Sun & Tang (2018)
Stromateidae	*Pampus minor*	Southern lesser pomfret		X	Li *et al*. (2019)
Terarogidae	*Paracentropogon*	NA	X		Kokita *et al*. (2006)

	rubripinnis				
Serranidae	*Paralabrax albomaculatus*	Camotillo	X		Bertolotti *et al*. (2015)
Serranidae	*Paralabrax clathratus*	Kelp bass	X		Selkoe *et al*. (2006)
Seranidae	*Paralabrax nebulifer*	Barred sand bass	X		Domínguez-Contreras *et al*. (2018); Paterson *et al*. (2015)
Paralichthyidae	*Paralichthys californicus*	California flounder		X	Craig *et al*. (2011)
Paralichthyidae	*Paralichthys olivaceus*	Bastard halibut	X	X	Kim *et al*. (2003); Kim *et al*. (2009)[b]; Kim *et al*. (2010)[c]; Li *et al*. (2011); Sekino & Hara (2001); Shigenobu *et al*. (2007); Takagi *et al*. (1999)[a]; Xu *et al*. (2012)
Haemulidae	*Parapristipoma trilineatum*	Chicken grunt	X		Kumagai *et al*. (2004)
Mullidae	*Parupeneus multifasciatus*	Manybar goatfish		X	Matias *et al*. (2013)
Sciaenidae	*Pennahia argentata*	Silver croaker		X	Han *et al*. (2008)
Pholidae	*Pholis fangi*	NA		X	Gao *et al*. (2019)[b]
Mugilidae	*Planiliza affinis*	Eastern keelback	X		Liu *et al*. (2019)[b]
Serranidae	*Plectropomus areolatus*	Squaretail coralgrouper	X		Ma *et al*. (2018)
Serranidae	*Plectropomus leopardus*	Leopard coralgrouper	X	X	Ding *et al*. (2009); Herwerden *et al*. (2009): Ma *et al*. (2018)
Serranidae	*Plectropomus maculatus*	Spotted coralgrouper		X	Evans *et al*. (2010)
Nototheniidae	*Pleuragramma antarctica*	Antarctic silverfish	X	X	Papetti *et al*. (2011); Zane *et al*. (2006)
Hexagrammidae	*Pleurogrammus*	Atka mackerel	X		Spies *et al*. (2005)

	monopterygius				
Pleuronectidae	*Pleuronectes platessa*	European plaice	X		Hemmer-Hansen (2007); Hoarau *et al*. (2002); Hoarau *et al*. (2004); Was *et al*. (2010); Watts *et al*. (1999); Watts *et al*. (2001); Watts *et al*. (2004); Watts *et al*. (2010)
Polyprionidae	*Polyprion americanus*	Wreckfish	X		Ball *et al*. (2000)
Polyprionidae	*Polyprion oxygeneios*	Hapuku wreckfish	X	X	Ball *et al*. (2000); Lane *et al*. (2016)
Pomacentridae	*Pomacentrus amboinensis*	Ambon damsel	X		Jones *et al*. (2008)
Pomacentridae	*Pomacentrus coelestis*	Neon damselfish	X	X	Frédérich *et al*. (2012); Liu *et al*. (2010)[b]; Liu *et al*. (2012); Miller-Sims *et al*. (2005)[b]
Pomacentridae	*Pomacentrus moluccensis*	Lemon damsel		X (π)	Drew *et al*. (2008)
Pomatomidae	*Pomatomus saltatrix*	Bluefish	X		Dos Santos *et al*. (2008); Miralles *et al*. (2014); Reid *et al*. (2016)
Gobiidae	*Pomatoschistus marmoratus*	Marbled goby	X		Berrebi *et al*. (2006)
Scorpaenidae	*Pontinus kuhlii*	Offshore rockfish		X	Catarino *et al*. (2013)
Carcharhinidae	*Prionace glauca*	Blue shark		X	Vella *et al*. (2017)
Lutjanidae	*Pristipomoides filamentosus*	Crimson jobfish	X	X	Gaither *et al*. (2011)[b]; Mzingirwa *et al*. (2019)
Lutjanidae	*Pristipomoides zonatus*	Oblique-banded snapper		X	Kennington *et al*. (2017)
Sciaenidae	*Protonibea diacanthus*	Blackspotted croaker	X		Taillebois *et al*. (2017)

Pleuronectidae	*Pseudopleuronectes americanus*	Winter flounder	X		Crivello *et al*. (2004); McClelland *et al*. (2005)
Pleuronectidae	*Pseudopleuronectes herzensteini*	Yellow striped flounder	X	X	Kim *et al*. (2007); Kim *et al*. (2010)[d]
Pleuronectidae	*Pseudopleuronectes yokohamae*	Marbled flounder	X	X (H_d)	Kim *et al*. (2009)[b]; Kitanishi *et al*. (2014); Lee *et al*. (2012); Sato *et al*. (2018)
Apogonidae	*Pterapogon kauderni*	Banggai cardinalfish	X		Hoffman *et al*. (2004); Hoffman *et al*. (2005)
Gobiidae	*Pterogobius elapoides*	NA	X		Nohara *et al*. (2009)[b]
Gobiidae	*Pterogobius zonoleucus*	NA	X		Nohara *et al*. (2009)[b]
Scorpaenidae	*Pterois miles*	Devil firefish		X	Kochzius & Blohm (2005)
Rachycentridae	*Rachycentron canadum*	Cobia	X		Divya *et al*. (2019); Phinchongsakuldit *et al*. (2013)
Rajidae	*Raja clavata*	Thornback ray	X	X	Chevolot *et al*. (2005); Chevolot *et al*. (2006)[a]; Chevolot *et al*. (2006)[b]; Ferrari *et al*. (2018); Vella *et al*. (2017)
Rajidae	*Raja miraletus*	Brown ray		X	Ferrari *et al*. (2018); Vella *et al*. (2017)
Rajidae	*Raja polystigma*	Speckled ray		X	Vella *et al*. (2017)
Rajidae	*Raja radula*	Rough ray		X	Vella *et al*. (2017)
Scombridae	*Rastrelliger kanagurta*	Indian mackerel		X	Akib *et al*. (2015); Pedrosa-Gerasmio *et al*. (2015)
Pleuronectidae	*Reinhardtius hippoglossoides*	Greenland halibut		X	Vis *et al*. (1997)

Rhincodontidae	*Rinchodon typus*	Whale shark	X	X	Vignaud *et al*. (2014)
Carcharhinidae	*Rhizoprionodon acutus*	Milk shark		X	Ovenden *et al*. (2011)
Lutjanidae	*Rhomboplites aurorubens*	Vermilion snapper	X		Bagley & Geller (1998); Bagley *et al*. (1999)
Tripterygiidae	*Ruanoho whero*	Spectacled triplefin		X	Hickey *et al*. (2009)
Scombridae	*Sarda sarda*	Atlantic bonito	X		Turan (2015)
Clupeidae	*Sardina pilchardus*	European pilchard	X		Gonzalez & Zardoya (2007); Ruggeri *et al*. (2012); Ruggeri *et al*. (2013)
Clupeidae	*Sardinella lemuru*	Bali sardinella		X	Pedrosa-Gerasmio *et al*. (2015)
Clupeidae	*Sardinella longiceps*	Indian oil sardine	X		Sebastian *et al*. (2017)
Clupeidae	*Sardinops sagax*	South American pilchard		X	Bowen & Grant (1997); García-Rodríguez *et al*. (2011)
Synodontidae	*Saurida elongata*	Slender lizardfish		X	Tu *et al*. (2016)
Blenniidae	*Scartella cristata*	Molly miller	X		Mackiewicz *et al*. (2005)
Scaridae	*Scarus ghobban*	Blue-barred parrotfish		X	Visram *et al*. (2010)
Scaridae	*Scarus psittacus*	Common parrotfish		X	Winters *et al*. (2010)
Sciaenidae	*Sciaenops ocellatus*	Red drum	X		Chapman *et al*. (2002); Karlsson *et al*. (2008)[a]; Karlsson *et al*. (2008)[b]; Turner *et al*. (1998)
Scombridae	*Scomber australasicus*	Blue mackerel	X	X	Scoles *et al*. (1989); Tang *et al*. (2009); Tzeng *et al*. (2007); Yagashita & Kobayashi (2008)

Scombridae	*Scomber japonicus*	Chub mackerel	X	X	Cha *et al*. (2010); Cheng *et al*. (2014); Cheng *et al*. (2015); Scoles *et al*. (1998); Tzeng *et al*. (2007); Yagashita & Kobayashi (2008)
Scombridae	*Scomber scombrus*	Atlantic mackerel	X	X	de Souza *et al*. (2006); Scoles *et al*. (1998); Papetti *et al*. (2013)
Scombridae	*Scombmeromorus brasiliensis*	Serra Spanish mackerel	X	X	Gold *et al*. (2010); Renshaw *et al*. (2009)[b]
Scombridae	*Scomberomorus cavalla*	King mackerel		X	Santa Brígida *et al*. (2007)
Scombridae	*Scomberomorus commerson*	Narrow-barred Spanish mackerel	X	X	Fauvleot & Borso (2011) Hoolihan *et al*. (2006); Radhakrishnan *et al*. (2018); van Herwerden *et al*. (2006)
Scombridae	*Scomberomorus concolor*	Monterey Spanish mackerel	X	X	Magallón-Gayón *et al*. (2016)
Scombridae	*Scomberomorus niphonius*	Japanese Spanish mackerel	X	X	Shui *et al*. (2009); Nakajima *et al*. (2014); Xing *et al*. (2009)[b]; Yokoyama *et al*. (2006)
Scombridae	*Scomberomorus semifasciatus*	Broad-barred king mackerel		X	Broderick *et al*. (2011)
Scombridae	*Scomberomorus sierra*	Pacific sierra		X	López *et al*. (2010)[b]
Scombropidae	*Scombrops boops*	Gnomefish		X	Noguchi *et al*. (2012)
Scombropidae	*Scombrops gilberti*	NA		X	Itoi *et al*. (2011)
Scophthalmidae	*Scophthalmus maximus*	Turbot	X		Bouza *et al*. (2002); Florin & Höglund (2007); Nielsen *et al*. (2004); Pardo *et al*. (2005); Vilas *et al*. (2015)

Scophthalmidae	*Scophthalmus rhombus*	Brill	X		Bouza *et al*. (2002)
Scyliorhinidae	*Scyliorhinus canicula*	Small-spotted catshark	X	X	Ferrari *et al*. (2018); Kousteni *et al*. (2015); Vella *et al*. (2017)
Scyliorhinidae	*Scyliorhinus stellaris*	Nursehound		X	Vella *et al*. (2017)
Sebastidae	*Sebastes alutus*	Pacific ocean perch	X		Palof *et al*. (2011)
Sebastidae	*Sebastes atrovirens*	Kelp rockfish	X		Gilbert-Horvath *et al*. (2006)
Sebastidae	*Sebastes auriculatus*	Brown rockfish	X		Buonaccorsi *et al*. (2005); Hauser *et al*. (2007)
Sebastidae	*Sebastes borealis*	Shortraker rockfish	X		Matala *et al*. (2004)[a]
Sebastidae	*Sebastes caurinus*	Copper rockfish	X		Buonaccorsi *et al*. (2002)
Sebastidae	*Sebastes crameri*	Darkblotched rockfish	X		Gomez-Uchida & Banks (2005)
Sebastidae	*Sebastes emphaeus*	Puget Sound rockfish		X (π)	Sitka *et al*. (2005)
Sebastidae	*Sebastes fasciatus*	Acadian redfish	X		Pampoulie & Daníelsdóttir (2008); Roques *et al*. (1999); Roques *et al*. (2001); Schmidt (2005); Valentin *et al*. (2014)
Sebastidae	*Sebastes flavidus*	Yellowtail rockfish	X	X	Hess *et al*. (2011)
Sebastidae	*Sebastes helvomaculatus*	Rosethorn rockfish		X	Rocha-Olivares & Vetter (1999)
Sebastidae	*Sebastes inermis*	Dark-banded rockfish	X		An *et al*. (2009)[a]; Gonzalez *et al*. (2009)
Sebastidae	*Sebastes macdonaldi*	Mexican rockfish	X		Rocha-Olivares & Sandoval-Castillo (2003)
Sebastidae	*Sebastes melanops*	Black rockfish	X		Lotterhos & Markel (2012); Lotterhos 2014); Miller *et al*. (2005)

Sebastidae	*Sebastes mentella*	Beaked redfish	X		Pampoulie & Daníelsdóttir (2008); Roques *et al*. (1999); Roques *et al*. (2001); Schmidt (2005); Stefánsson *et al*. (2009)[a]; Stefánsson *et al*. (2009)[b]; Valentin *et al*. (2014)
Sebastidae	*Sebastes miniatus*	Vermilion rockfish		X	Hyde & Vetter (2009)
Sebastidae	*Sebastes mystinus*	Blue rockfish	X		Burford & Larson (2007); Burford & Bernardi (2008); Burford (2009)
Sebastidae	*Sebastes norvegicus*	Golden redfish	X		Pampoulie & Daníelsdóttir (2008); Pampoulie *et al*. (2009); Roques *et al*. (1999); Schmidt (2005)
Sebastidae	*Sebastes oculatus*	Patagonian redfish		X	Nuñez *et al*. (2010)
Sebastidae	*Sebastes paucispinis*	Bocaccio rockfish	X		Matala *et al*. (2004)[b]
Sebastidae	*Sebastes pinniger*	Canary rockfish	X		Gomez-Uchida (2006); Hyde *et al*. (2008)
Sebastidae	*Sebastes polyspinus*	Northern rockfish	X		Gharret *et al*. (2012)
Sebastidae	*Sebastes rastrelliger*	Grass rockfish	X		Buonaccorsi *et al*. (2004); Westerman *et al*. (2005)
Sebastidae	*Sebastes ruberrimus*	Yelloweye rockfish	X		Siegle *et al*. (2013); Yamanaka & Lacko (2001)
Sebastidae	*Sebastes schlegelii*	Korean rockfish	X	X	An *et al*. (2009)[b]; An *et al*. (2012)[b]**; Gao** *et al*. (2016); Yoshida *et al*. (2005); Zhang *et al*. (2014)[b]
Sebastidae	*Sebastes thompsoni*	Goldeye rockfish	X		Sekino & Hara (2001)
Sebastidae	*Sebastes viviparus*	Norway redfish	X		Pampoulie & Daníelsdóttir (2008); Roques *et al*. (1999)

Scorpaenidae	*Sebastiscus marmoratus*	False kelpfish	X	X	Deng *et al*. (2015); Li *et al*. (2014)[a]; Liu *et al*. (2019)[c]; Yin *et al*. (2012)
Carangidae	*Selar crumenopthalmus*	Bigeye scad		X	Pedrosa-Gerasmio *et al*. (2015)
Labridae	*Semicossyphus darwini*	Galápagos sheephead wrasse	X		Poortvliet *et al*. (2013)
Labridae	*Semicossyphus pulcher*	California sheephead	X		Poortvliet *et al*. (2013)
Carangidae	*Seriola dumerili*	Greater amberjack	X	X (H_d)	Babbucci *et al*. (2006); Renshaw *et al*. (2006); Šegvić-Bubić *et al*. (2016)
Carangidae	*Seriola lalandi*	Yellowtail amberjack	X	X	Nugruho *et al*. (2001); Sepúlveda & González (2017)
Carangidae	*Seriola rivoliana*	Longfin yellowtail		X (H_d)	Šegvić-Bubić *et al*. (2016)
Centrolophidae	*Seriolella brama*	Common warehou		X	Robinson *et al*. (2008)
Serranidae	*Serranus cabrilla*	Comber	X		Carreras-Carbonell *et al*. (2006); Galarza *et al*. (2009)[a]; Schunter *et al*. (2011)[b]
Centrolophidae	*Seriolella punctata*	Silver warehou		X	Robinson *et al*. (2008)
Siganidae	*Siganus fuscescens*	Mottled spinefoot	X	X	Li *et al*. (2013)[c]; Ravago-Gotanco *et al*. (2010); Ravago-Gotanco & Junio-Meñez (2010); Ravago-Gotanco *et al*. (2018)
Siganidae	*Siganus guttatus*	Orange-spotted spinefoot		X	Iwamoto *et al*. (2009); Iwamoto *et al*. (2012)
Siganidae	*Siganus spinus*	Little spinefoot	X	X	Iwamoto *et al*. (2009); Priest *et al*. (2012)
Sillaginidae	*Sillago japonica*	Japanese sillago	X	X	Gao *et al*. (2019)[c]; Ueno *et al*. (2013); Umino *et al*. (2013)

Sillaginidae	*Sillago parvisquamis*	Small-scale sillago	X		Ueno *et al*. (2013); Umino *et al*. (2013)
Apogonidae	*Siphamia tubifer*	Tubifer cardinalfish	X		Alpermann *et al*. (2014)
Soleidae	*Solea senegalensis*	Senegalese sole	X		Díaz-Ferguson *et al*. (2012); Funes *et al*. (2004)
Soleidae	*Solea solea*	Common sole	X	X	Cuveliers *et al*. (2011); Cuveliers *et al*. (2012); Garoia *et al*. (2006)
Somniosidae	*Somniosus rostratus*	Little sleeper shark		X	Vella *et al*. (2017)
Sparidae	*Sparus aurata*	Gilthead seabream	X	X	Alarcón *et al*. (2004); Chaoui *et al*. (2012); Coscia *et al*. (2012); De Innocentiis *et al*. (2004); Franchini *et al*. (2012); Karaiskou *et al*. (2009); Šegvić-Bubić *et al*. (2011)
Apogonidae	*Sphaeramia orbicularis*	Orbiculate cardinalfish		X	Gotoh *et al*. (2009)
Sphyraenidae	*Sphyraena barracuda*	Great barracuda		X	Daly-Engel *et al*. (2012)
Sphyraenidae	*Sphyraena viridensis*	Yellowmouth barracuda		X (H_d)	Milana *et al*. (2014)
Sphyrnidae	*Sphyrna lewini*	Scalloped hammerhead	X	X	Castillo-Olguín *et al*. (2012); Duncan *et al*. (2006); Nance *et al*. (2011); Ovenden *et al*. (2011)
Gasterosteidae	*Spinachia spinachia*	Sea stickleback	X		Jones *et al*. (1998)[b]
Clupeidae	*Sprattus fuegensis*	Falkland sprat	X		Canales-Aguirre *et al*. (2016); Ferrada-Fuentes *et al*. (2014)
Clupeidae	*Sprattus sprattus*	European sprat	X	X	Debes *et al*. (2008); Dailianis *et al*. (2008); Glover *et al*. (2011); Limborg *et*

					al. (2009); Limborg *et al*. (2012)
Squalidae	*Squalus acanthias*	Picked dogfish	X		McCauley *et al*. (2004); Thorburn *et al*. (2018)
Squalidae	*Squalus blainville*	Longnose spurdog	X	X	Kousteni *et al*. (2016); Vella *et al*. (2017)
Pomacentridae	*Stegastes partitus*	Bicolor damselfish	X		Hepburn *et al*. (2009); Purcell *et al*. (2009); Pusack *et al*. (2014); Salas *et al*. (2010); Williams *et al*. (2003)
Myctophidae	*Stenobrachius leucopsarus*	Northern lampfish		X	Kojima *et al*. (2009)
Myctophidae	*Stenobrachius nannochir*	Giant lanternfish		X	Kojima *et al*. (2009)
Monacanthidae	*Stephanolepis cirrhifer*	Threadsail filefish	X		An *et al*. (2011); An *et al*. (2013)[a]
Labridae	*Symphodus melops*	Corkwing wrasse	X		Knutsen *et al*. (2013)
Labridae	*Symphodus tinca*	East Atlantic peacock wrasse	X		Galarza *et al*. (2006); Galarza *et al*. (2009)[a]
Syngnathidae	*Synghathus auliscus*	Barred pipefish	X		Wilson (2006)
Syngnathidae	*Syngnathus floridae*	Dusky pipefish	X		Mobley *et al*. (2010)
Syngnathidae	*Syngnathus leptorhynchus*	Bay pipefish		X	Wilson (2006)
Syngnathidae	*Syngnathus scovelli*	Gulf pipefish	X		Jones & Avise (1997)
Syngnathidae	*Syngnathus typhle*	Broadnosed pipefish	X		Jones *et al*. (1999)
Tetraodontidae	*Takifugu rubripes*	Japanese pufferfish		X	Reza *et al*. (2011)
Labridae	*Thalassoma bifasciatum*	Bluehead	X	X	Haney *et al*. (2007); Purcell *et al*. (2006); Williams *et al*. (2004)[b]
Monacanthidae	*Thamnaconus*	Leser-spotted		X	Li *et al*. (2014)[b]

	hypargyreus	leatherjacket			
Monacanthidae	*Thamnaconus modestus*	Black scraper	X		An *et al*. (2013)[b]
Monacanthidae	*Thamnaconus septentrionalis*	Greenfin horse-faced filefish	X		Xu *et al*. (2009)
Scombridae	*Thunnus alalunga*	Albacore	X		Davies *et al*. (2011); Montes *et al*. (2012); Takagi *et al*. (1999)[b]; Takagi *et al*. (2001)
Scombridae	*Thunnus albacares*	Yellowfin tuna	X	X	Aguila *et al*. (2015); Dammannagoda *et al*. (2008); Diaz-Jaimes & Uribe-Alcocer (2006); Kunal *et al*. (2013); Qiu & Miyamoto (2011); Takagi *et al*. (1999)[b]
Scombridae	*Thunnus obesus*	Bigeye tuna	X	X	Durand *et al*. (2005); Gonzalez *et al*. (2008); Takagi *et al*. (1999)[b]; Wu *et al*. (2014)
Scombridae	*Thunnus orientalis*	Pacific bluefin tuna	X		Morishima *et al*. (2009); Qiu & Miyamoto (2011); Takagi *et al*. (1999)[b]
Scombridae	*Thunnus thynnus*	Atlantic bluefin tuna	X		Riccioni *et al*. (2010); Takagi *et al*. (1999)[b]; Vella *et al*. (2009); Vella *et al*. (2016); Viñas *et al*. (2011)
Torpedinidae	*Torpedo marmorata*	Marbled electric ray		X	Vella *et al*. (2017)
Sciaenidae	*Totoaba macdonaldi*	Totoaba	X	X	Valenzuela-Quiñonez *et al*. (2014); Valenzuela-Quiñonez *et al*. (2016)
Plesiopidae	*Trachinops caudimaculatus*	Southern hulafish	X		McWilliam *et al*. (2015)
Carangidae	*Trachinotus carolinus*	Florida pompano	X		Seyoum *et al*. (2007)
Carangidae	*Trachinotus falcatus*	Permit	X		Seyoum *et al*. (2007)

Carangidae	*Trachinotus goodei*	Great pompano	X		Seyoum *et al*. (2007)
Carangidae	*Trachurus japonicus*	Japanese jack mackerel	X		Chang *et al*. (2009)
Carangidae	*Trachurus mediterraneus*	Mediterranean horse mackerel		X	Bektas & Belduz (2008)
Carangidae	*Trachurus murphyi*	Chilean jack mackerel	X	X	Canales-Aguirre *et al*. (2010); Cárdenas *et al*. (2009)
Carangidae	*Trachurus picturatus*	Blue jack mackerel		X	Bektas & Belduz (2008)
Carangidae	*Trachurus trachurus*	Atlantic horse mackerel	X	X	Bektas & Belduz (2008); Kasapidis & Magoulas (2008)
Nototheniidae	*Trematomus bernacchii*	Emerald rockcod	X		Van de Putte *et al*. (2009); Van de Putte *et al*. (2012)[b]
Nototheniidae	*Trematomus eulepidotus*	Blunt scalyhead	X		Damerau *et al*. (2012); Van de Putte *et al*. (2009)
Nototheniidae	*Trematomus hansoni*	Striped rockcod	X		Van de Putte *et al*. (2009); Van de Putte *et al*. (2012)[b]
Nototheniidae	*Trematomus newnesi*	Dusky rockcod	X		Damerau *et al*. (2012); Van de Putte *et al*. (2009); Van de Putte *et al*. (2012)[b]; Van Houdt *et al*. (2006)
Nototheniidae	*Trematomus scotti*	Crowned rockcod	X		Van de Putte *et al*. (2009)
Carcharhinidae	*Triaenodon obesus*	Whitetip reef shark		X	Whitney *et al*. (2012)
Triakidae	*Triakis megalopterus*	Sharptooth houndshark	X		Maduna *et al*. (2017)
Tripterygiidae	*Tripterygion delais*	Black-faced blenny	X		Carreras-Carbonell *et al*. (2004); Galarza

					et al. (2009)[a]
Rhinobatidae	*Trygonorrhina dumerilii*	Sothern fiddler ray		X (H_d)	Donnellan *et al*. (2015)
Rhinobatidae	*Trygonorrhina fasciata*	Eastern fiddler ray		X (H_d)	Donnellan *et al*. (2015)
Urotrygonidae	*Urobatis helleri*	Haller's round ray	X		Plank *et al*. (2010)
Phycidae	*Urophycis tenuis*	White hake	X		Bradbury *et al*. (2009); Roy *et al*. (2010)
Muraenidae	*Uropterygius micropterus*	Tidepool snake moray		X	Huang *et al*. (2018)
Pleuronectidae	*Verasper moseri*	Barfin flounder	X		Ma & Chen (2009); Miao *et al*. (2009); Ortega-Villaizán Romo *et al.* (2003); Ortega-Villaizán Romo *et al.* (2006)[a]; Ortega-Villaizán Romo *et al.* (2006)[b]
Pleuronectidae	*Verasper variegatus*	Spotted halibut	X	X	Ortega-Villaizán Romo *et al*. (2006)[a]; Ortega-Villaizán Romo *et al*. (2006)[b]; Sekino *et al*. (2011)
Xiphiidae	*Xiphias gladius*	Swordfish		X	Alvarado Bremer *et al*. (2005); Muths *et al*. (2005)
Labridae	*Xyrichtys novacula*	Pearly razorfish		X (H_d)	Nirchio *et al*. (2019)
Rajidae	*Zearaja chilensis*	Yellownose skate	X	X	Vargas-Caro *et al*. (2017)
Rajidae	*Zearaja maugeana*	Maugean skate	X		Weltz *et al*. (2018)
Acanthuridae	*Zebrasoma flavescens*	Yellow tang		X	Eble *et al*. (2011)[b]
Zoarcidae	*Zoarces viviparus*	Viviparous eelpout	X		Kinitz *et al*. (2013)
Gobiidae	*Zosterisesor ophiocephalus*	Grass goby	X		Bisol *et al*. (2007)

DATA REFERENCES

Aboim MA, Rogers AD, Menezes GM, Maggioni R & Pearson CVM (2003) Isolation of polymorphic microsatellite markers for the demersal fish *Helicolenus dactylopterus* (Dela Roche 1809). *Molecular Ecology Notes*, **3**, 18-20. doi:10.1046/j.1471-8286.2003.00337.x

Aguila RD, Perez SKL, Catacutan BJN, Lopez GV, Barut NC & Santos MD (2015) Distinct yellowfin tuna (*Thunnus albacares*) stocks detected in Western and Central Pacific Ocean (WCPO) using DNA microsatellites. *PLoS ONE*, **10**, e0138292. doi:10.1371/journal.pone.0138292

Akib NAM, Tam BM, Phumee P, Abidin MZ, Tamadoni S, Mather PB & Nor SAM (2015) High connectivity in *Rastrelliger kanagurta*: Influence of historical signatures and migratory behaviour inferred from mtDNA cyctochrome *b*. *PLoS ONE*, **10**, e0119749. doi:10.1371/journal.pone.0119749

Alarcón JA, Magoulas A, Georgakopoulos T, Zouros E & Alvarez MC (2004) Genetic comparison of wild and cultivated European populations of the gilthead sea bream (*Sparus aurata*). *Aquaculture*, **230**, 65-80. doi:10.1016/S0044-8486(03)00434-4

Almojil D, Cliff G & Spaet JLY (2018) Weak population structure of the spot-tail shark *Carcharhinus sorrah* and the blacktail shark *C. limbatus* along the coasts of the Arabian Peninsula, Pakistan, and South Africa. *Ecology an Evolution*, **8**, 9536-9549. doi:10.1002/ece3.4468

Alpermann TJ, Plieske J, Mal AO, Gon O & Berumen ML (2014) Isolation and characterization of opolymorphic microsatellite loci for *Siphamia ubifer* Weber (Perciformes: Apogonidae). *Conservation Genetics Resources*, **6**, 1031-1034. doi:10.1007/s12686-014-0278-5

Alvarado Bremer JR, Mejuto J, Gómez-Márquez J, Boán F, Carpintero P, Rodríguez JM, Viñas J, Greig TW & Ely B (2005) Hierarchical analyses of genetic variation of samples from breeding and feeding grounds confirm the genetic partitioning of northwest Atlantic and South Atlantic populations of swordfish (*Xiphias gladius* L.). *Journal of Experimental Marine Biology and Ecology*, **327**, 167-182. doi:10.1016/j.embe.2005.06.02

An HS, Kim KS, Lee HY, Kim EM & Kwon MG (2009a) Isolation and characterization of polymorphic microsatellite markers for the black rockfish *Sebastes inermis*. *Genes & Genomics*, **31**, 29-34. doi:10.1007/BF03191135

An HS, Park JY, Kim M-J, Lee EY & Kim KK (2009b) Isolation and characterization of microsatellite markers for the heavily exploited rockfish *Sebastes schlegeli*, and cross-species amplification in four related *Sebastes* spp. *Conservation Genetics*, **10**, 1969-1972. doi:10.1007/s10592-009-9870-8

An HS, Hong SW, Lee JU, Park JY & Kim K (2010) Genetic diversity of wild and farmed black sea bream populations in Jeju. *Animal Cells and Systems*, **14**, 37-44. doi:10.1080/19768351003765020

An HS, Hong SW, Kim EM & Myeong JI (2011) Comparative genetic diversity of wild and hatchery populations of Korean threadsail filefish *Stephanolepis cirrhifer* using cross-species microsatellite markers. *Genes & Genomics*, **33**, 605-611. doi:10.1007/s13258-011-0109-y

An HS, Kim JW, Lee JW, Kim SK, Lee BI, Kim DJ & Kim YC (2012a) Development and characterization of microsatellite markers for an endangered species, *Epinephelus bruneus*, to establish a conservation program. *Animal Cells and Systems*, **16**, 50-56. doi:10.1080/19768354.2011.611255

An H, Kim M-J, Park K, Cho K, Bae B, Kim J & Myeong J-I (2012b) Genetic diversity and population structure in the heavily exploited Korean rockfish, *Sebastes schlegeli*, in Korea. *Journal of the World Aquaculture Society*, **43**, 73-83. doi:10.1111/j.1749-7345.2011.00544.x

An HS, Lee JW, Hong SW, Myeong JI & An CM (2013a) Population genetic structure of the Korean threadsail filefish (*Stephanolepis cirrhifer*) based on microsatellite marker analysis. *Biochemical Systematics and Ecology*, **50**, 397-405. doi:10.1016/j.bse.2013.06.001

An HS, Lee JW, Park JY & Jung HT (2013b) Genetic structure of the Korean black scraper *Thamnaconus modestus* inferred from microsatellite marker analysis. *Molecular Biology Reports*, **40**, 3445-3456. doi:10.1007/s11033-012-2044-7

An HS, Kang HW, Han HS, Park JY, Hong CG, Park J, Myeong JI & An CM (2014) Genetic differences between the wild and hatchery-produced populations of Korean short barbeled grunter (*Hapalogenys nitens*) determined with microsatellite markers. *Genetics and Molecular Research*, **13**, 8901-8912. doi:10.4328/2014.October.31.5

Anderson JD & McDonald DL (2007) Morphological and genetic investigations of two west Gulf of Mexico menhadens (*Brevoortia* spp.) *Journal of Fish Biology*, **70**, 139-147. doi:10.1111/j.1095-8649.2007.01326.x

Anderson JD & Karel WJ (2007) Genetic evidence for asymmetric hybridization between menhadens (*Brevoortia* spp.) from peninsular Florida. *Journal of Fish Biology*, **71**, 235-249. doi:10.1111/j.1095-8649.2007.01597.x

Anderson JD & Karel WJ (2009) A genetic assessment of current management strategies for spotted seatrout in Texas. *Marine and Coastal Fisheries: Dynamics, Management, and Ecosystem Science*, **1**, 121-132. doi:10.1577/C09-001.1

Anderson JD, McDonald DL, Sutton GR & Karel WJ (2009) Evolutionary associations between sand seatrout (*Cynoscion arenarius*) and silver seatrout (*C. nothus*) inferred from morphological characters, mitochondrial DNA, and microsatellite markers. *Fishery Bulletin*, **107**, 13-23.

Anderson JD & Karel WJ (2010) Population genetics and dynamics of spotted seatrout in the estuarine waters of Texas. *Fisheries and Aquaculture Journal*, **2010**, FAJ2.

Anderson JD & Karel WJ (2014) Limited genetic structure of Gulf menhaden (*Brevoortia patronus*), as revealed by microsatellite markers developed for the genus *Brevoortia* (Glupeidae). *Fishery Bulletin*, **112**, 71-81. doi:10.7755/FB.112.1.5

Anderson JD, O'Leary SJ & Cooper PT (2019) Population structure of Atlantic croakers from the Gulf of Mexico: Evaluating a single-stock hypothesis using a genomic approach. *Marine and Coastal Fisheries: Dynamics, Management, and Ecosystem Science*, **11**, 3-16. doi:10.1002/mcf2.10055

Andrews KR, Moriwake VN, Wilcox C, Grau EG, Kelley C, Pyle RL & Bowen BW (2014) Phylogeographic analyses of submesophotic snappers *Etelis coruscans* and *Etelis "marshi"* (Family Lutjanidae) reveal concordant genetic structure across the Hawaiian Archipelago. *PLoS ONE*, **9**, e91665. doi:10.1371/journal.pone.0091665

Andreotti S, von der Heyden S, Henriques R, Rutzen M, Meÿer M, Oosthuizen H & Matthee CA (2016) New insights into the evolutionary history of white sharks, *Carcharodon carcharias*. *Journal of Biogeography*, **43**, 328-339. doi:10.1111/jbi.12641

Angiulli E, Sola L, Ardizzone G, Fassatoui C & Rossi AR (2016) Phylogeography of the common pandora *Pagellus erythrinus* in the central Mediterranean Sea: Sympatric mitochondrial lineages and genetic homogeneity. *Marine Biology Research*, **12**, doi:10.1080/17451000.2015.1069355

Antoni L, Emerick N & Saillant E (2011) Genetic variation of gray triggerfish in U.S. waters of the Gulf of Mexico and western Atlantic Ocean as inferred from mitochondrial DNA sequences. *North American Journal of Fisheries Management*, **31**, 714-721. doi:10.1080/02755947.2011.611861

Antoro S, Na-Nakorn U & Koedprang W (2006) Study of genetic diversity of orange-spotted grouper, *Epinephelus coioides*, from Thailand and Indonesia using microsatellite markers. *Marine Biotechnology*, **8**, 17-26. doi:10.1007/s10126-005-5026-0

Appleyard SA, Williams R & Ward RD (2004) Population genetic structure of Patagonian toothfish in the West Indian Ocean sector of the Southern Ocean. *CCAMLR Science*, **11**, 21-32.

Araneda C, Lam N, Iturra P, Jilberto F, Cordova V & Gallardo P (2017) Utility of five SSR markers for genetic diversity and paternity exclusion analysis in the Patagonian toothfish. *Latin American Journal of Aquatic Research*, **45**, 188-192. doi:10.3856/vol45-issue1-fulltext-18

Babbucci M, Zane L, Andaloro F & Patarnello T (2006) Isolation and characterization of microsatellite loci from yellowtail *Seriola dumerilii* (Perciformes: Carangidae). *Molecular Ecology Notes*, **6**, 1126-1128. doi:10.1111/j.1471-8286.2006.01459.x

Bagley MJ & Geller JB (1998) Characterization of microsatellite loci in the vermillion snapper *Rhomboplites aurorubens* (Percoidei: Lutjanidae). *Molecular Ecology*, **7**, 1089-1090.

Bagley MJ, Lindquist DG & Geller JB (1999) Microsatellite variation, effective population size, and population genetic structure of vermilion snapper, *Rhomboplites aurorubens*, off the southeastern USA. *Marine Biology*, **134**, 609-620. doi:10.1007/s002270050576

Bahri-Sfar L, Lemaire C, Ben Hassine OK & Bonhomme F (2000) Fragmentation of sea bass populations in the western and eastern Mediterranean as revealed by microsatellite polymorphism. *Proceedings of the Royal Society B: Biological Sciences*, **267**, 929-935. doi:10.1098/rspb.2000.1092

Ball AO, Sedberry GR, Zatcoff MS, Chapman RW & Carlin JL (2000) Population structure of the wreckfish *Polyprion americanus* determined with microsatellite genetic markers. *Marine Biology*, **137**, 1077-1090. doi:10.1007/s00227000039

Ball AO, Beal MG, Chapman RW & Sedberry GR (2007) Population structure of red porgy, *Pagrus pagrus*, in the Atlantic Ocean. *Marine Biology*, **150**, 1321-1332. doi:10.1007/s00227-006-0425-y

Barnes TC, Junge C, Myers SA, Taylor MD, Rogers PJ, Ferguson GJ, Lieschke JA, Donnellan SC & Gillanders BM (2015) Population structure in a wide-ranging coastal teleost (*Argyrosomus japonicus*, Sciaenidae) reflects marine biogeography across southern Australia. *Marine and Freshwater Research*, **67**, 1103-1113. doi:10.1071/MF1504

Barton DP, Taillebois L, Taylor J, Crook DA, Saunders T, Hearnden M, Greig A, Welch DJ, Newman SJ, Travers MJ, Saunders RJ, Errity C, Maher S, Dudgeon C & Ovenden J (2018) Stock structure of *Lethrinus laticaudis* (Lethrinidae) across northern Australia determined using genetics, otolith microchemistry and parasite assemblage composition. *Marine and Freshwater Research*, **69**, 487-501. doi:10.1071/MF17087

Beacham TD, Schweigert JF, MacConnachie C, Le KD, Labaree K & Miller KM (2002) *Population structure of herring (*Clupea pallassi*) in British Columbia determined by microsatellites, with comparisons to southeast Alaska and California.* Fisheries & Oceans Canada, Science, Canadian Science Advisory Secretariat.

Bekkevold D, Aandré C, Dahlgren TG, Clausen LAW, Torstensen E, Mosegaard H, Carvalho GR, Christensen TB, Norlinder E & Ruzzante DE (2005) Environmental correlates of population differentiation in Atlantic herring. *Evolution*, **59**, 2656-2668. doi:10.1111/j.0014-3820.2005.tb00977.x

Bekkevold D, Gross R, Arula T, Helyar SJ & Ojaveer H (2016) Outlier loci detect intraspecific biodiversity amongst spring and autumn spawning herring across local scales. *PLoS ONE*, **11**, e0148499. doi:10.1371/journal.pone.0148499

Bektas Y & Belduz AO (2008) Molecular phylogeny of Turkish *Trachurus* species (Perciformes: Carangidae) inferred from mitochondrial DNA analyses. *Journal of Fish Biology*, **73**, 1228-1248. doi:10.1111/j.1095-8649.2008.01996.x

Beldade R, Cudney-Buerno R, Raimondi PT & Bernardi G (2009) Molecular Ecology Resources Primer Development Consortium. Permanent genetic resources added to molecular ecology resources database, 1 January 2009-30 April 2009. *Molecular Ecology Resources*, **9**, 1375-1379. doi:10.1111/j.1755-0998.2009.02746.x

Beldade R, Jackson AM, Cudney-Bueno R, Raimondi P & Bernardi G (2014) Genetic structure among spawning aggregations of the gulf coney *Hyporthodus acanthistius*. *Marine Ecology Progress Series*, **499**, 193-201. doi:10.3354/meps1063

Bentley BP, Harvey ES, Newman SJ, Welch DJ, Smith AK & Kennington WJ (2014) Local genetic patchiness but no regional differences between Indo-West Pacific populations of the dogtooth tuna *Gymnosarda unicolor*. *Marine Ecology Progress Series*, **506**, 267-2777. doi:10.3354/meps10819

Bentzen P, Taggart CT, Ruzzante DE & Cook D (1996) Microsatellite polymorphism and the population structure of Atlantic cod (*Gadus morhua*) in the northwest Atlantic. *Canadian Journal of Fisheries and Aquatic Sciences*, **53**, 2706-2721. doi:10.1139/f96-238

Bernal-Ramírez JH, Adcock GJ, Hauser L, Carvalho GR & Smith PJ (2003) Temporal stability of genetic population structure in the New Zealand snapper, *Pagrus auratus*, and relationship to coastal currents. *Marine Biology*, **142**, 567-574. doi:10.1007/s00227-002-0972-9

Bernardi G (2008) Isolation and characterization of 12 microsatellites from the black surfperch, *Embiotoca jacksoni*, a reef fish that lacks a pelagic larval phase. *Molecular Ecology Resources*, **8**, 1512-1514. doi:10.1111/j.1755-0998.2008.02346.x

Bernardi G, Beldade R, Holbrook SJ & Schmitt RJ (2012) Full-sibs in cohorts of newly settled coral reef fishes. *PLoS ONE*, **7**, e44953. doi:10.1371/journal.pone.0044953

Berrebi P, Lasserre B, Barbisan F & Zane L (2006) Isolation of microsatellite loci and cross-species amplifications in three gobiid fish of the genus *Pomatoschistus*. *Molecular Ecology Notes*, **6**, 724-727. doi:10.1111/j.1471-8286.2006.01322.

Berry O, England P, Fairclough D, Jackson G & Greenwood J (2012a) Microsatellite DNA analysis and hydrodynamic modelling reveal the extent of larval transport and gene flow between management zones in an exploited marine fish (*Glaucosoma hebraicum*). *Fisheries Oceanography*, **21**, 243-254. doi:10.1111/j.1365-2419.2012.00623.x

Berry O, England P, Marriott RJ, Burridge C & Newman SJ (2012b) Understanding age-specific dispersal in fishes through hydrodynamic modelling, genetic simulations and microsatellite DNA analysis. *Molecular Ecology*, **21**, 2145-2159. doi:10.1111/j.1365-294X.2012.05520.x

Bertolotti AC, Griffiths SM, Truelove NK, Box SJ, Preziosi RF & de Leon PS (2015) Isolation and characterization of 10 polymorphic microsatellite loci for the endangered Galapagos-endemic whitespotted sandbass (*Paralabrax albomaculatus*). *PeerJ*, **3**, e1253. doi:10.7717/pperj.1253

Bester-van der Merwe AE, Bitalo D, Cuevas JM, Ovenden J, Hernández S, da Silva C, McCord M & Roodt-Wilding R (2017) Population genetics of Southern Hemisphere tope shark (*Galeorhinus galeus*): Intercontinental divergence and constrained gene flow at different geographical scales. *PLoS ONE*, **12**, e0184481. doi:10.1371/journal.pone.0184491

Bisol PM, Gallini A, Prevedello S, Rianni E, Bernardinelli E, Franco A & Zane L (2007) Low variation at allozyme loci and differences between age classes at microsatellites in grass goby (*Zosterisessor ophiocephalus*) populations. *Hydrobiologia*, **577**, 151-159. doi:10.1007/s10750-006-0424-

Blanco G, Borrell YJ, Cagigas E, Vázquez E & Prado JAS (2006) A new set of highly polymorphic microsatellites for the white and black anglerfish (Lophiidae). *Molecular Ecology Notes*, **6**, 767-769. doi:10.1111/j.1471-8286.2006.01336.x

Blower DC, Pandolfi JM, Bruce BD, Gomez-Cabrera MC & Ovenden JR (2012) Population genetics of Australian white sharks reveals fine-scale spatial structure, transoceanic dispersal events and low effective population sizes. *Marine Ecology Progress Series*, **455**, 229-244. doi:10.3354/meps09659

Boissin E, Thorrold SR, Braun CD, Zhou Y, Clua EE & Planes S (2019) Contrasting global, regional and local patterns of genetic structure in gray reef shark populations from the Indo-Pacific region. *Scientific Reports*, **9**, 15816.

Bonin MC, Saenz-Agudelo P, Harrison HB, Nanninga GB, van der Meer MH, Mansour H, Perumal S, Jones GP & Berumen ML (2016) Characterization and cross-amplification of microsatellite markers in four species of anemonefish (Pomacentridae, *Amphiprion* spp.). *Marine Biodiversity*, **46**, 135-140. doi:10.1007/s12526-015-0336-6

Borrell YJ, Piñera JA, Prado JAS & Blanco G (2012) Mitochondrial DNA and microsatellite genetic differentiation in the European anchovy *Engraulis encrasicolus* L. *ICES Journal of Marine Science*, **69**, 1357-1371. doi:10.1093/icesjms/fss129

Bouza C, Presa P, Castro J, Sánchez L & Martínez P (2002) Allozyme and microsatellite diversity in natural and domestic populations of turbot (*Scophthalmus maximus*) in comparison with other Pleuronectiformes. *Canadian Journal of Fisheries and Aquatic Sciences*, **59**, 1460-1473. doi:10.1139/f02-114

Bowen BW & Grant WS (1997) Phylogeography of the sardines (*Sardinops* spp.): Assessing biogeographic models and population histories in temperate upwelling zones. *Evolution*, **51**, 1601-1610. doi:10.1111/j.1558-5646.1997.tb01483.x

Bowen BW, Bass AL, Muss A, Carlin J & Robertson DR (2006) Phylogeography of two Atlantic squirrelfishes (Family Holocentridae): Exploring links between pelagic larval duration and population connectivity. *Marine Biology*, **149**, 899-913. doi:10.1007/s00227-006-0252-1

Bradbury IR, Snelgrove PVR, Bentzen P, De Young B, Gregory RS & Morris CJ (2009) Structural and functional connectivity of marine fishes within a semi-enclosed Newfoundland fjord. *Journal of Fish Biology*, **75**, 1393-1409. doi:10.1111/j.1095-8649.2009.02391.x

Broderick D, Ovenden JR, Buckworth RC, Newman SJ, Lester RJG & Welch DJ (2011) Genetic population structure of grey mackerel *Scomberomorus semifasciatus* in northern Australia. *Journal of Fish Biology*, **79**, 633-661. doi:10.1111/j.1095-8649.2011.03055.x

Buchholz-Sørensen M & Vella A (2016) Population structure, genetic diversity, effective population size, demographic history and regional connectivity patterns of the endangered dusky grouper, *Epinephelus marginatus* (Teleostei: Serranidae), within Malta's fisheries management zone. *PLoS ONE*, **11**, e0159864. doi:10.1371/journal.pone.0159864

Burford MO & Larson RJ (2007) Genetic heterogeneity in a single year-class from a panmictic population of adult blue rockfish (*Sebastes mystinus*). *Marine Biology*, **151**, 451-465. doi:10.1007/s00227-006-0475-1

Burford MO & Bernardi G (2008) Incipient speciation within a subgenus of rockfish (*Sebastosomus*) provides evidence of recent radiations within an ancient species flock. *Marine Biology*, **154**, 701-717. doi:10.1007/s00227-008-0963-6

Burford MO (2009) Demographic history, geographical distribution and reproductive isolation of distinct lineages of blue rockfish (*Sebastes mystinus*), a marine fish with a high dispersal potential. *Journal of Evolutionary Biology*, **22**, 1471-1486. doi:10.1111/j.1420-9101.2009.01760.x

Buonaccorsi VP, Kimbrel CA, Lynn EA & Vetter RD (2002) Population structure of copper rockfish (*Sebastes caurinus*) reflects postglacial colonization and contemporary patterns of larval dispersal. *Canadian Journal of Fisheries and Aquatic Sciences*, **59**, 1374-1384. doi:10.1139/f02-101

Buonaccorsi VP, Westerman M, Stannard J, Kimbrell C, Lynn E & Vetter RD (2004) Molecular genetic structure suggests limited larval dispersal in grass rockfish, *Sebastes rastrelliger*. *Marine Biology*, **145**, 779-788. doi:10.1007/s00227-004-1362-2

Buonaccorsi VP, Kimbrell CA, Lynn EA &Vetter RD (2005) Limited realized dispersal and introgressive hybridization influence genetic structure and conservation strategies for brown rockfish, *Sebastes auriculatus*. *Conservation Genetics*, **6**, 697-713. doi:10.1007/s10592-005-9029-1

Burridge CP & Smolenski AJ (2003) Lack of genetic divergence found with microsatellite DNA markers in the tarakihi *Nemadactylus macropterus*. *New Zealand Journal of Marine and Freshwater Research*, **37**, 223-230. doi:10.1080/00288330.2003.9517160

Burridge CP & England PR (2009) Tri- and tetranucleotide microsatellites in dhufish *Glaucosoma hebracium* (Perciformes). *Molecular Ecology Resources*, **9**, 948-951. doi:10.1111/j.1755-0998.2008.02508.x

Buston PM, Bogdanowicz SM, Wong A & Harrison RG (2007) Are cownfish groups composed of close relatives? An analysis of microsatellite DNA variation in *Amphiprion percula*. *Molecular Ecology*, **16**, 3671-3678. doi:10.1111/j.1365-294X.2007.03421.x

Calò A, Muñoz I, Pérez-Ruzafa Á, Vergara-Chen C & García-Charton JA (2016) Spatial genetic structure in the saddled sea bream (*Oblada Melanura* [Linnaeus, 1758]) suggests multi-scaled patterns of connectivity between protected and unprotected areas in the Western Mediterranean Sea. *Fisheries Research*, **176**, 30-38. doi:10.1016/j.fishres.2015.12.001

Campo D & Garcia-Vazquez E (2010) Evolutionary history of the four-spotted megrim (*Lepidorhombus boscii*) and speciation time within the genus based on mitochondrial genes analysis. *Journal of Sea Research*, **64**, 360-368. doi:10.1016/j.seares.2010.05.004

Canales-Aguirre CB, Ferrada S & Galleguillos R (2010) Isolation and characterization of microsatellite loci for the jack mackerel (*Trachurus Murphyi* Nichols, 1920). *Conservation Genetics*, **11**, 1235-1237. doi:10.1007/s10592-009-9932-y

Canales-Aguirre CB, Ferrada-Fuentes S, Galleguillos R & Hernández CE (2016) Genetic structure in a small pelagic fish coincides with a marine protected area: Seascape genetics in Patagonian fjords. *PLoS ONE*, **11**, e0160670. doi:10.1371/journal.pone.0160670

Canino MF, Spies IB & Hauser L (2005) Development and characterization of novel di- and tetranucleotide microsatellite markers in Pacific cod (*Gadus macrocephalus*). *Molecular Ecology Notes*, **5**, 908-910. doi:10.1111/j.1471-8286.2005.01109.x

Canino MF, Spies IB, Cunningham KM, Hauser L & Grant WS (2010) Multiple ice-age refugia in Pacific cod, *Gadus macrocephalus*. *Molecular Ecology*, **19**, 4339-4351. doi:10.1111/j.1365-294X.2010.04815.x

Cantanese G, Crespo A, Zuasti E, Perez L & Funes V (2008) Isolation and characterization of polymorphic microsatellites for the bastard sole (*Microchirus azevia*). *Molecular Ecology Resources*, **8**, 1111-1113. doi:10.1111/j.1755-0998.2008.02172.x

Cárdenas L, Silva AX, Magoulas A, Cabezas J, Poulin E & Ojeda FP (2009) Genetic population structure in the Chilean jack mackerel, *Trachurus murphyi* (Nichols) across the South-eastern Pacific Ocean. *Fisheries Research*, **100**, 109-115. doi:10.1016/j.fishres.2009.06.01

Carlsson J, Shephard S, Coughlan J, Trueman CN, Rogan E & Cross TF (2011) Fine-sòe population structure in a deep-sea teleost (orange roughy, *Hoplostethus atlanticus*). *Deep-Sea Research I*, **58**, 627-636. doi:10.1016/j.dsr.2011.03.009

Carr SM, Snellen AJ, Howse KA & Wroblewski JS (1995) Mitochondrial DNA sequence variation and geetic stock structure of Atlantic cod (*Gadus morhua*) from bay and offshore locations on the Newfoundland continental shelf. *Molecular Ecology*, **4**, 79-88. doi:10.1111/j.1365-294X.1995.tb00194.x

Carreras-Carbonell J, MacPherson E & Pascual M (2004) Isolation and characterization of microsatellite loci in *Tripterygion delaisi*. *Molecular Ecology Notes*, **4**, 438-439. doi:10.1111/j.1471-8286.2004.00688.x

Carreras-Carbonell J, MacPherson E & Pascual M (2006) Characterization of 12 microsatellites markers in *Serranus cabrilla* (Pisces: Serranidae). *Molecular Ecology Notes*, **6**, 204-206. doi:10.1111/j.1471-8286.2005.01193.

Carson EW, Saillant EA, Renshaw MA, Commings NJ & Gold JR (2011) Population structure, long-term connectivity, and effective size of mutton snapper (*Lutjanus analis*) in the Caribbean Sea and Florida Keys. *Fishery Bulletin*, **109**, 416-428.

Castillo AGF, Alvarez P, Garcia-Vazquez E (2005) Population structure of *Merluccius merluccius* along the Iberian Peninsula coast. *ICES Journal of Marine Science*, **62**, 1699-1704. doi:10.1016/j.icesjms.2005.06.001

Castillo-Olguín E, Uribe-Alcocer M & Díaz-Jaimes P (2012) Assessment of the population genetic structure of *Sphyrna lewini* to identify conservation units in the Mexican Pacific. *Ciencias Marinas*, **38**, 635-652. doi:10.7773/cm.v38i4.2110

Catarino D, Stefanni S & Menezes GM (2013) Size distribution and genetic diversity of the offshore rockfish (*Pontinus kuhlii*) from three Atlantic archipelagos and seamounts. *Deep Sea Research Part II: Topical Studies in Oceanography*, **98**, 160-169. doi:10.1016/j.dsr2.2013.02.010

Catarino D, Stefanni S, Jorde PE, Menezes GM, Company JB, Neat F & Knutsen H (2017) The role of the Strait of Gibraltar in shaping the genetic structure of the Mediterranean Grenadier, *Coryphaenoides mediterraneus*, between the Atlantic and Mediterranean Sea. *PLoS ONE*, **12**, e0174988. doi:10.1371/journal.pone.017498

Ceballos SG, Lessa EP, Victorio MF & Fernández DA (2012) Phylogeography of the sub-Antarctic notothenioid fish *Eleginops maclovinus*: Evidence of population expansion. *Marine Biology*, **159**, 499-505. doi:10.1007/s00227-011-1830-4

Ceballos SG, Lessa EP, Licandeo R & Fernández DA (2016) Genetic relationships between Atlantic and Pacific populations of the notothenioid fish *Eleginops maclovinus*: The footprints of Quaternary glaciations in Patagonia. *Heredity*, **116**, 372-377.

Cerda JM, López F, Palacios-Fuentes P & Ojeda FP (2019) Genetic structure analysis of *Girella laevifrons* populations in central Chile. *Journal of Applied Ichthyology*, **35**, 1147-1153. doi:10.1111/jai.13952

Cha HK, An HS, Choi JH, Kang S, Park JY & Kim KK (2010) Isolation and characterization of polymorphic microsatellite markers for genetic analysis of chub mackerel (*Scomber japonicus*). *Conservation Genetics Resources*, **2**, 7-9. doi:10.1007/s12686-009-9123-

Chang DS, An HS, Oh TY, Lee JB, Park JY & Kim KK (2009) Eleven new microsatellite markers in jack mackerel (*Trachurus japonicus*) derived from an enriched genomic library. *Genes & Genomics*, **31**, 397-402. doi:10.1007/BF0319185

Chaoui L, Gagnaire P, Guinand B, Quignard J, Tsigenopoulos C, Kara MH & Bonhomme F (2012) Microsatellite length variation in candidate genes correlates with habitat in the gilthead sea bream *Sparus aurata*. *Molecular Ecology*, **21**, 5497-5511. doi:10.1111/mec.12062

Chapman RW, Ball AO & Mash LR (2002) Spatial homogeneity and temporal heterogeneity of red drum (*Sciaenops ocellatus*) microsatellites: effective population sizes and management implications. *Marine Biotechnology*, **4**, 589-603. doi:10.1007/s10126-002-0038-5

Charrier G, Coombs SH, McQuinn IH & Laroche J (2007) Genetic structure of whiting *Merlangius merlangus* in the northeast Atlantic and adjacent waters. *Marine Ecology Progress Series*, **220**, 201-211. doi:10.3354/meps330201

Chen S, Liu T, Li Z & Gao T (2008) Genetic population structuring and demographic history of red spotted grouper (*Epinephelus akaara*) in South and East China Sea. *African Journal of Biotechnology*, **7**, 3554-3562.

Chen S, Xing S, Xu G, Liao X & Yang J (2009) Isolation and characterization of 10 polymorphic microsatellite loci from small yellow croaker (*Pseudosciaena polyactis*). *Conservation Genetics*, **10**, 1469. doi:10.1007/s10592-008-9762-3

Chen C, Tzeng C & Chiu T (2010) Morphological and molecular analyses reveal separations among spatiotemporal populations of anchovy (*Engraulis japonicus*) in the southern east China Sea. *Zoological Studies*, **49**, 270-282.

Chen W & Cheng Q (2013) Development of thirty-five novel polymorphic microsatellite markers in *Pseudosciaena polyactis* (Perciformes: Sciaenidae) and cross-species amplification in closely related species, *Pseudosciaena crocea*. *Biochemical Systematics and Ecology*, **47**, 111-115. doi:10.1016/j.bse.2012.11.007

Cheng Y, Jin X, Shi G, Wang R & Xu T (2011) Genetic diversity and population structure of miiuy croaker populations in East China Sea revealed by the mitochondrial DNA control region sequence. *Biochemical Systematics and Ecology*, **39**, 718-724. doi:10.1016/j.bse.2011.06.009

Cheng Q, Zhu Y & Chen X (2014) High polymorphism and moderate differentiation of chub mackerel, *Scomber japonicus* (Perciformes: Scombridae), along the coast of China revealed by fifteen novel microsatellite markers. *Conservation Genetics*, **15**, 1021-1035. doi:10.1007/s10592-014-0596-x

Cheng J, Yanagimoto T, Song N & Gao T (2015) Population genetic structure of chub mackerel *Scomber japonicus* in the Northwestern Pacific inferred from microsatellite analysis. *Molecular Biology Reports*, **42**, 373-382. doi:10.1007/s11033-014-3777-

Chevolot M, Reusch TB, Boele-Bos S, Stam WT & Olsen JL (2005) Characterization and isolation of DNA microsatellite primers in *Raja clavate* L. (thornback ray, Rajidae). *Molecular Ecology Notes*, **5**, 427-429. doi:10.1111/j.1471-8286.2005.00951.x

Chevolot M, Ellis JR, Hoarau G, Rijnsdorp AD, Stam WT & Olsen JL (2006a) Population structure of the thornback ray (*Raja clavata* L.) in British waters. *Journal of Sea Research*, **56**, 305-316. doi:10.1016/j.seares.2006.05.005

Chevolot M, Hoarau G, Rijnsdorp AD, Stam WT & Olsen JL (2006b) Phylogeography and population structure of thornback rays (*Raja clavate* L., Rajidae). *Molecular Ecology*, **15**, 3693-3705. doi:10.1111/j.1365-294X.2006.03043.x

Chevolot M, Wolfs PHJ, Pálsson J, Rijnsdorp AD, Stam, WT & Olsen JL (2007) Population structure and historical demography of the thorny skate (*Amblyraja radiata*, Rajidae) in the North Atlantic. *Marine Biology*, **151**, 1275-1286. doi:10.1007/s00227-006-0556-1

Chopelet J, Helyar S, Mann B & Mariani S (2009) Novel polymorphic microsatellite loci for the protogynous hermaphrodite slinger sea bream (*Chrysoblephus puniceus*, Sparidae). *Molecular Ecology Resources*, **9**, 1223-1226. doi:10.1111/j.1755-0998.2009.02615.x

Correia AT, Faria R, Alexandrino P, Antunes C, Isidro EJ & Coimbra J (2006) Evidence for genetic differentiation in the European conger eel *Conger conger* based on mitochondrial DNA analysis. *Fisheries Science*, **72**, 20-27. doi:10.1111/j.1444-2906-2006.01111.

Correia AT, Ramos AA, Barros F, Silva G, Hamer P, Morais P, Cunha RL & Castilho R (2012) Population structure and connectivity of the European conger eel (*Conger conger*) across the north-eastern Atlantic and western Mediterranean: Integrating molecular and otolith elemental approaches. *Marine Biology*, **159**, 1509-1525. doi:10.1007/s00227-012-1936-3

Coscia I, Vogiatzi E, Kotoulas G, Tsigenopoulos CS & Mariani S (2012) Exploring neutral and adaptive processes in expanding populations of the gilthead sea bream, *Sparus aurata* L., in the North-East Atlantic. *Heredity*, **108**, 537-546.

Coscia I, Castilho R, Massa-Gallucci A, Sacchi C, Cunha RL, Stefanni S, Helyar SJ, Knutsen H & Mariani S (2018) Genetic homogeneity in the deep-sea grenadier *Macrourus berglax* across the North Atlantic Ocean. *Deep Sea Research Part I: Oceanographic Research Papers*. **132**, 60-67. doi:10.1016/j.dsr.2017.12.001

Coughlan J, Connolly P, Cross TF, Mall N, Rinville O & Galway C (2010) Eight novel tetranucleotide microsatellite loci for orange roughy (*Hoplestethus atlanticus*). *Molecular Ecology Resources*, **10**, 576-579.

Craig MT, Eble JA, Bowen BW & Robertson DR (2007) High genetic connectivity across the Indian and Pacific Oceans in the reef fish *Myripristis berndti* (Holocentridae). *Marine Ecology Progress Series*, **334**, 245-254. doi:10.3354/meps334245

Craig MT, Graham RT, Torres RA, Hyde JR, Freitas MO, Ferreira BP, Hostim-Silva M, Gerhardinger LC, Bertoncini AA & Robertson DR (2009) How many species of goliath grouper are there? Cryptic genetic divergence in a threatened marine fish and the resurrection of a geopolitical species. *Endangered Species Research*, **7**, 167-174. doi:10.3354/esr00117

Craig MT, Fodrie EJ, Allen LG, Chartier LA & Toonen RJ (2011) Discordant phylogeographic and biogeographic breaks in California halibut. *Bulletin, Southern California Academy of Sciences*, **110**, 141-151. doi:10.3160/0038-3872-110.3.141

Crivello JF, Danila DJ, Lorda E, Keser M & Roseman EF (2004) The genetic stock structure of larval and juvenile winter flounder larvae in Connecticut waters of eastern Long Island Sound and estimations of larval entrainment. *Journal of Fish Biology*, **65**, 62-76. doi:10.1111/j.0022-1112.2004.00424.x

Cunha RL, COscia I, Madeira C, Mariani S, Steffani S & Castilho R (2012) Ancient divergence in the trans-oceanic deep-sea shark *Centroscymnus crepidater*. *PLoS ONE*, **7**, e49196. doi:10.1371/journal.pone.0049196

Cunningham KM (2007) *Population genetics of Pacific cod (*Gadus macrocephalus*): Evidence for large and small scale population structure*, University of Washington.

Cunningham KM, Canino MF, Spies IB & Hauser L (2009) Genetic isolation by distance and localized fjord population structure in Pacific cod (*Gadus macrocephalus*): Limited effective dispersal in the northeastern Pacific Ocean. *Canadian Journal of Fisheries and Aquatic Sciences*, **66**, 153-166. doi:10.1139/F08-199

Cushman EL, Jue NK, Strand AE & Sotka EE (2009) Evaluating the demographic significance of genetic homogeneity using a coalescent-based simulation: A case study with gag (*Mycteroperca microlepis*). *Canadian Journal of Fisheries and Aquatic Sciences*, **66**, 1821-1830. doi:10.1139/F09-140.

Cuveliers EL, Volckaert FAM, Rijnsdorp AD, Larmuseau MHD & Maes GE (2011) Temporal genetic stability and high effective population size Despite fisheries-induced life-history trait evolution in the North Sea sole. *Molecular Ecology*, **20**, 3555-3568. doi:10.1111/j.1365-294X.2011.05196.x

Cuveliers EL, Larmuseau MHD, Hellemans B, Verherstraeten SLNA, Volckaert FAM & Maes GE (2012) Multi-marker estimate of genetic connectivity of sole (*Solea solea*) in the North-East Atlantic Ocean. *Marine Biology*, **159**, 1239-1253. doi:10.1007/s00227-012-1905-x

D'Amato ME (2006) Demographic expansion and subtle differentiation in the long-tailed hake *Macruronus magellanicus*:: Evidence from microsatellite data. *Marine Biotechnology*, **8**, 189-201. doi:10.1007/s10126-005-5075-4

D'Amato ME, Lunt DH & Carvalho GR (1999) Microsatellite markers for the hake *Macruronus magellanicus* amplify other gadoid fish. *Molecular Ecology*, **8**, 1086-1088. doi:10.1046/j.1365-294X.1999.00655_7.x

D'Anatro A, Pereira AN & Lessa EP (2011) Genetic structure of the white croaker, *Micropogonias furnieri* Desmarest 1823 (Perciformes: Sciaenidae) along Uruguayan coasts: Contrasting marine, estuarine, and lacustrine populations. *Environmental Biology of Fishes*, **91**, 407. doi:10.1007/s10641-011-9799-x

da Silva Cortinhas MC, Kersanach R, Proietti M, Dumont LFC, D'Incao F, Lacerda ALF, Prata PS, Matoso DA, Noleto RB, Ramsdorf W, Boni TA, Prioli AJ & Cestari MM (2016) Genetic structuring among silverside fish (*Atherinella brasiliensis*) populations from different Brazilian regions. *Estuarine, Coastal and Shelf Science*, **178**, 148-157. doi:10.1016/j.ecss.2016.06.007

Dahle G, Quintela M, Johansen T, Westgaard J, Besnier F, Aglen A, Jørstad KE & Glover KA (2018) Analysis of coastal cod (*Gadus morhua* L.) sampled on spawning sites reveals a genetic gradient throughout Norway's coastline. *BMC Genetics*, **19**, 42. doi:10.1186/s12863-018-0625-8

Dailianis T, Limborg M, Hanel R, Bekkevold D, Lagnel J, Magoulas A & Tsigenopopulos CS (2008) Characterization of nine polymorphic Microsatellite markers in sprat (*Sprattus sprattus* L.). *Molecular Ecology Resources*, **8**, 861-863. doi:10.1111/j.1755-0998.2008.02091.x

Daley RK, Appleyard SA & Koopman M (2012) Genetic catch verification to support recovery plans for deepsea gulper sharks (genus *Centrophorus*, family Centrophoridae) – an Australian example using the 16S gene. *Marine and Freshwater Research*, **63**, 708-714. doi:10.1071/MF11264

Daley-Engel TS, Randall JE & Bowen BW (2012) Is the great barracuda (*Sphyraena barracuda*) a reef fish or a pelagic fish? The phylogeographic perspective. *Marine Biology*, **159**, 975-985. doi:10.1007/s00227-012-1878-9

Damerau M, Matschiner M, Salzburger W & Hanel R (2012) Comparative population genetics of seven notothenioid fish species reveals high levels of gene flow along ocean currents in the southern Scotia Arc, Antarctica. *Polar Biology*, **35**, 1073-1086. doi:10.1007/s00300-012-1155-x

Damerau M, Matschiner M, Salzburger W & Hanel R (2014a) Population divergence despite long pelagic larval stages: Lessons from crocodile icefishes (Channichthyidae). *Molecular Ecology*, **23**, 284-299. doi:10.1111/mec.12612

Damerau M, Salzburger W & Hanel R (2014b) Population genetic structure of *Lepidonotothen larseni* revisited: *cytb* and microsatellites suggest limited connectivity in the Southern Ocean. *Marine Ecology Progress Series*, **517**, 251-263. doi:10.3354/meps11061

Dammannagoda ST (2007) *Genetic stock structure and inferred migratory patterns of skipjack tuna (*Katsuwonus pelamis*) and yellowfin tuna (*Thunnus albacares*) in Sri Lankan waters*. Queensland University of Technology.

Dammannagoda ST, Hurwood DA & Mather PB (2008) Evidence for fine geographical scale heterogeneity in gene frequencies in yellowfin tuna (*Thunnus albacares*) from the north Indian Ocean around Sri Lanka. *Fisheries Research*, **90**, 147-157. doi:10.1016/j.fishres.2007.10.006

Dammannagoda ST, Hurwood DA & Mather PB (2011) Genetic analysis reveals two stocks of skipjack tuna (*Katsuwonus pelamis*) in the northwestern Indian Ocean. *Canadian Journal of Fisheries and Aquatic Sciences*, **68**, 210-223. doi:10.1139/F10-136

Danancher D & Garcia-Vazquez E (2009) Population differentiation in megrim (*Lepidorhombus whiffiagonis*) and four spotted megrim (*Lepidorhombus boscii*) across Atlantic and Mediterranean waters and implications for wild stock management. *Marine Biology*, **156**, 1869-1880. doi:10.1007/s00227-009-1219-9

Davies CA, Gosling EM, Was A, Brophy D & Tysklind N (2011) Microsatellite analysis of albacore tuna (*Thunnus alalunga*): Population genetic structure in the North-East Atlantic Ocean and Mediterranean Sea. *Marine Biology*,**158**, 2727-2740. doi:10.1007/s00227-011-1772-x

De Innocentiis S, Sola L, Cataudella S & Bentzen P (2001) Allozyme and microsatellite loci provide discordant estimates of population differentiation in the endangered dusky grouper (*Epinephelus marginatus*) within the Mediterranean Sea. *Molecular Ecology*, **10**, 2163-2175. doi:10.1046/j.1365-294X.2001.01371.x

De Innocentiis S, Lesti A, Livi S, Rossi AR, Crosetti D & Sola L (2004) Microsatellite markers reveal population structure in gilthead sea bream *Sparus auratus* from the Atlantic Ocean and Mediterranean Sea. *Fisheries Science*, **70**, 852-859. doi:10.1111/j.1444-2906.2004.00879.x

de Souza LL, Sévigny J, Chanut J, Barry WF & Grégoire F (2006) High genetic variability in the mtDNA control region of a Northwestern Atlantic teleost, *Scomber scombrus* L. *Canadian Technical Report of Fisheries and Aquatic Sciences*, 2625.

de Souza AS, Dias Jr. EA, Galetti Jr. PM, Machado EG, Pichorim M & Molina WF (2015) Wide-range genetic connectivity of Coney, *Cephalopholis fulva* (Epinephelidae), through oceanic islands and continental Brazilian coast. *Annals of the Brazilian Academy of Sciences*, **87**, 121-136. doi:10.1590/0001-3765201520130411

de Souza AS, Dias Jr. EA, Perez MF, Cioffi MB, Bertollo LAC, Garcia-Machado E, Vallinoto MNS, Galetti Jr. PM & Molina WF (2019) Phylogeography and historical demography of two sympatric Atlantic snappers: *Lutjanus analis* and *L. jocu*. *Frontiers in Marine Science*, **6**, 545. doi:10.3389/fmars.2019.00545

Debes PV, Zachos FE & Hanel R (2008) Mitochondrial phylogeography of the European sprat (*Sprattus sprattus*) L., Clupeidae) reveals isolated climatically vulnerable populations in the Mediterranean Sea and range expansion in the northeast Atlantic. *Molecular Ecology*, **17**, 3872-3888. doi:10.1111/j.1365-294X.2008.03872.

Deng HW, Li ZB, Dai G, Yuan Y, Ning YF, Shangguan JB & Huang YS (2015) Isolation of new polymorphic microsatellite markers from the marbled rockfish *Sebastiscus marmoratus*. *Genetics and Molecular Research*, **14**, 758-762. doi:10.4238/2015.January.30.19

Deng Z, Wang X, Xu S, Gao T & Han Z (2019) Population genetic structure and selective pressure on the mitochondrial ATP6 gene of the Japanese sand lance *Ammodytes personatus* Girard. *Journal of the Marine Biological Association of the United Kingdom*, **99**, 1409-1416. doi:10.1017/S0025315419000225

Díaz-Ferguson E, Cross I, Barrios M, Pino A, Castro J, Bouza C, Martínez P & Rebordinos L (2012) Genetic characterization, based on microsatellite loci, of *Solea senegalensis* (Soleidae, Pleuronectiformes) in Atlantic coast populations of the SW Iberian Peninsula. *Ciencias Marinas*, **38**, 129-142.

Díaz-Jaimes P & Uribe-Alcocer M (2006) Spatial differentiation in the eastern Pacific yellowfin tuna revealed by microsatellite variation. *Fisheries Science*, **72**, 590-596. doi:10.1111/j.1444-2906.2006.01188.x

DiBattista JD, Feldheim KA & Bowen BW (2011a) Microsatellite DNA markers to resolve population structure and hybridization of two closely related surgeonfish species, *Acanthurus nigricans* and *Acanthurus leucosternon*. *Conservation Genetics Resources*, **3**, 159-162. doi:10.1007/s12686-010-9313-3

DiBattista JD, Wilcox C, Craig MT, Rocha LA & Bowen BW (2011b) Phylogeography of the Pacific blueline surgeonfish, *Acanthurus nigrosis*, reveals high genetic connectivity and a cryptic endemic species in the Hawaiian Archipelago. *Journal of Marine Sciences*, **2011**, 839134. doi:10.1155/2011/83913

DiBattista JD, Rocha LA, Craig MT, Feldheim KA & Bowen BW (2012) Phylogeography of two closely related Indo-Pacific butterflyfishes reveals divergent evolutionary histories and discordant results from mtDNA and microsatellites. *Journal of Heredity*, **103**, 617-629. doi:10.1093/jhered/ess056

Ding S, Zeng H, Wang Y, Pan Y & Shi X (2009) Characterization of eight polymorphic microsatellite loci for the leopard coralgrouper (*Plectropomus leopardus* Lacepède). *Molecular Ecology Resources*, **9**, 1485-1487. doi:10.1111/j.1755-0998.2009.02690.x

Divya PR, Linu J, Mohitha C, Kathirvelpandian A, Manoj P, Basheer VS & Gopalakrishnan A (2019) Deciphering demographic history and fine scale population structure of cobia, *Rachycentron canadum* (Pisces: Rachycentridae) using microsatellite and mitochondrial markers. *Marine Biodiversity*, **49**, 381-393. doi:10.1007/s12526-017-0817-x

Dohna TA, Timm J, Hamid L & Kochzius M (2015) Limited connectivity and a phylogeographic break characterize populations of the pink anemonefish, *Amphiprion perideraion*, in the Indo-Malay Archipelago: Inferences from a mitochondrial and microsatellite loci. *Ecology and Evolution*, **5**, 1717-1733. Doi:10.1002/ece3.1455

Domingues RR, Bruels CC, Gadig OBF, Chapman DD, Hilsdorf AWS & Shivji MS (2019) Genetic connectivity and phylogeography of the night shark (*Carcharhinus signatus*) in the western Atlantic Ocean: Implications for conservation management. *Aquatic Conservation: Marine and Freshwater Ecosystems*, **29**, 102-114. doi:10.1002/aqc.2961

Domínguez-Contreras JF, Munguia-Vega A, Castillo-Lopez A, Gracia-Olea JA, Blasco CM & Peckham SH (2018) Characterization by next-generation sequencing of 24 new microsatellite loci for the barred sand-bass, *Paralabrax nebulifer* (Girard, 1854), from the Baja California Peninsula, Mexico. *Marine Biodiversity*, **48**, 2207-2210. doi:10.1007/s12526-017-0687-2

Donnellan SC, Foster R, Junge C, Huveneers C, Rogers P, Kilian A & Bertozzi T (2015) Fiddling with the proof: The magpie fiddler ray is a colour pattern variant of the common southern fidler ray (Rhinobatidae:*Trygonorrhina*). *Zootaxa* **3981**, 367-384. doi:10.11646/zootaxa.3981.3.3

Dos Santos SMR, Klopper AW, Oosthuizen CJ & Bloomer P (2008) Isolation and characterization of polymorphic tetranucleotide microsatellite loci in the pelagic perciform fish *Pomatomus saltatrix* (Linnaeus, 1766) from South Africa. *Molecular Ecology Resources*, **8**, 1065-1067. doi:10.1111/j.1755-0998.2008.02156.x

Drew J, Allen GR, Kaufman L & Barber PH (2008) Endemism and regional color and genetic differences in five putatively cosmopolitan reef fishes. *Conservation Biology*, **22**, 965-976. doi:10.1111/j.1523-1739.2008.01011.x

Drinan DP, Galindo HM, Loher T & Hauser L (2016) Subtle genetic population structure in Pacific halibut *Hippoglossus stenolepis*, **89**, 2571-2594. doi:10.1111/jfb.13148

Duncan KM, Martin AP, Bowen BW & de Couet HG (2006) Global phylogeography of the scalloped hammerhead shark (*Sphyrna lewini*). *Molecular Ecology*, **15**, 2239-2251. doi:10.1111/j.1365-294X.2006.02933.x

Duncan M, James N, Fennessy ST, Mutombene RJ & Mwale M (2015) Genetic structure and consequences of stock exploitation of *Chrysoblephus puniceus*, a commercially important sparid in the South West Indian Ocean. *Fisheries Research*, **164**, 64-72. doi:10.1016/j.fishres.2014.10.019

Durand J, Collet A, Chow S, Guinand B & Borsa P (2005) Nuclear and mitochondrial DNA markers indicate unidirectional gene flow of Indo Pacific to Atlantic bigeye tuna (*Thunnus obesus*) populations, and their admixture off southern Africa. *Marine Biology*, **147**, 313-322. doi:10.1007/s00227-005-1564-2

Eble JA, Rocha L, Craig MT & Bowen BW (2011a) Not all larvae stay close to home: Insights into marine population connectivity with a focus on the brown surgeonfish (*Acanthurus nigrofuscus*). *Journal of Marine Sciences*, **2011**, 518516. doi:10.1155/2011/518516

Eble JA, Toonen RJ, Sorenson L, Basch LV, Papastamatiou YP & Bowen BW (2011b) Escaping paradise: larval export from Hawaii in an Indo Pacific reef fish, the yellow tang *Zebrasoma flavescens*. *Marine Ecology Progress Series*, **428**, 245-258. doi:10.3354/meps09083

Eiríksson GM & Árnason E (2014) Mitochondrial DNA sequence variation in whiting *Merlangius merlangus* in the North East Atlantic. *Environmental Biology of Fishes*, **97**, 103-110. doi:10.1007/s10641-013-0143-5

Evans RD, van Herwerden L, Russ GR & Frisch AJ (2010) Strong genetic but not spatial subdivision of two reef fish species targeted by fishers on the Great Barrier Reef. *Fisheries Research*, **102**, 16-25. doi:10.1016/j.fishres.2009.10.002

Exadactylos A, Vafidis D, Tsigenopoulos CS & Gkafas GA (2019) High connectivity of the white seabream (*Diplodus sargus*, L. 1758) in the Aegean Sea, Eastern Mediterranean Basin. *Animals*, **9**, 979. doi:10.3390/ani9110979

Farnsworth CA, Bellwood DR & van Herwerden L (2010) Genetic structure across the GBR: Evidence from short-lived gobies. *Marine Biology*, **157**, 945-953. doi:10.1007/s00227-009-1375-y

Fauvelot C & Borsa P (2011) Patterns of genetic isolation in a widely distributed pelagic fish, the narrow-barred Spanish mackerel (*Scomberomorus commerson*). *Biological Journal of the Linnean Society*, **104**, 886-902. doi:10.1111/j.1095-8312.2011.01754.x

Feldheim K, Stow AJ, Ahonen H, Chapman DD, Shivji M, Peddemors V & Wintner S (2007) Polymorphic microsatellite markers for studies of the conservation and reproductive genetics of imperiled sand tiger sharks (*Carcharias taurus*). *Molecular Ecology Notes*, **7**, 1366-1368. doi:10.1111/j.1471-8286.2007.01888.x

Félix-Hackradt FC, Hackradt CW, Pérez-Ruzafa Á & García-Charton JA (2013) Discordant patterns of genetic connectivity between two sympatric species, *Mullus barbatus* (Linnaeus, 1758) and *Mullus surmuletus* (Linnaeus, 1758), in south-western Mediterranean Sea. *Marine Environmental Research*, **92**, 23-34. doi:10.1016/j.marenvres.2013.08.008

Fernandez-Silva I, Randall JE, Coleman RR, DiBattista JD, Rocha LA, Reimer JD, Meyer CG & Bowen BW (2015) Yellow tails in the Red Sea: phylogeography of the Indo-Pacific goatfish *Mulloidichthys flavolineatus* reveals isolation in peripheral provinces and cryptic evolutionary lineages. *Journal of Biogeography*, **42**, 2402-2413. doi:10.1111/jbi.1259

Ferrada-Fuentes S, Galleguillos R, Canales-Aguirre CB, Love CN, Jones KL & Lance SL (2014) Development and characterization of thirty-three microsatellite markers for the Patagonian sprat, *Sprattus fuegensis* (Jenyns, 1842), using paired-end Illumina shotgun sequencing. *Conservation Genetics Resources*, **6**, 833-836. doi:10.1007/s12686-014-0281.x

Ferrari A, Tinti F, Maresca VB, Velonà A, Cannas R, Thasitis I, Costa FO, Follesca MC, Golani D, Hemida F, Helyar SJ, Mancusi C, Mulas A, Serena F, Sion L, Stagioni M & Cariani A (2018) Natural history and molecular evolution of demersal Mediterranean sharks and skates inferred by comparative phylogeographic and demographic analyses. *PeerJ*, **6**, e5560. doi:10.7717/peerj.5560

Florin A & Höglund J (2007) Absence of population structure of turbot (*Psetta maxima*) in the Baltic Sea. *Molecular Ecology*, **16**, 115-126. doi:10.1111/j.1365-294X.2006.03120.x

Franchini P, Sola L, Crosetti D, Milana V & Rossi AR (2012) Low levels of population genetic structure in the gilthead sea bream, *Sparus aurata*, along the coast of Italy. *ICES Journal of Marine Science*, **69**, 41-50. doi:10.1093/icesjms/fsr175

Francisco SM, Vieira MN & Almada VC (2006) Genetic structure and historical demography of the shanny *Lipophrys pholis* in the Portuguese coast based on mitochondrial DNA analysis. *Molecular Phylogenetics and Evolution*, **39**, 288-292. doi:10.1.1.902.853

Frédérich B, Liu SV & Dai C (2012) Morphological and genetic divergences in a coral reef damselfish, *Pomacentrus coelestis*. *Evolutionary Biology*, **39**, 359-370. doi:10.1007/s11692-011-9158-z

Freitas AS, da Silva R, Sampaio I, Schneider H (2017) The mitochondrial control region reveals genetic structure in southern kingcroaker populations on the coast of the Southwestern Atlantic. *Fisheries Research*, **191**, 87-94. doi:10.1016/j.fishres.2017.03.008

Friess C & Sedberry GR (2011) Genetic evidence for a single stock of the deep-sea teleost *Beryx decadactylus* in the North Atlantic Ocean as inferred from mtDNA control region analysis. *Journal of Fish Biology*, **78**, 466-478. doi:10.1111/j.1095-8649.2010.02857.

Fritsch M, Morizur Y, Lambert E, Bonhomme F & Guinand B (2007) Assessment of sea bass (*Dicentrarchus labrax*, L.) stock delimitation in the Bay of Biscay and the English Channel based on mark-recapture and genetic data. *Fisheries Research*, **83**, 123-132. doi:10.1016/j.fishres.2006.09.002

Froukh T & Kochzius M (2007) Genetic population structure of the endemic fourline wrasse (*Larabicus quadrilineatus*) suggests limited larval dispersal distances in the Red Sea. *Molecular Ecology*, **16**, 1359-1367. doi:10.1111/j.1365-294X.2007.03236.x

Fune V, Zuasti E, Catanese G, Infante C & Manchado M (2004) Isolation and characterization of ten microsatellite loci for Senegal sole (*Solea Senegalensis* Kaup). *Molecular Ecology Notes*, **4**, 339-341. doi:10.1111/j.1471-8286.2004.00690.x

Gaither MR, Bowen BW, Toonen RJ, Planes S, Messmer V, Earle J & Robertson DR (2010b) Genetic consequences of introducing allopatric lineages of bluestriped snapper (*Lutjanus kasmira*) to Hawaii. *Molecular Ecology*, **19**, 1107-1121. doi:10.1111/j.1365-294X.2010.04535.x

Gaither MR, Toonen RJ, Robertson DR, Planes S & Bowen BW (2010a) Genetic evaluation of marine biogeographical barriers: Perspectives from two widespread Indo-Pacific snappers (*Lutjanus kasmira* and *Lutjanus fulvus*). *Journal of Biogeography*, **37**, 133-147. doi:10.1111/j.1365-2699.2009.02188x

Gaither MR, Bowen BW, Bordenave T, Rocha LA, Newman SJ, Gomez JA, van Herwerden L & Craig MT (2011a) Phylogeography of the reef fish *Cephalopholis argus* (Epinephelidae) indicates Pleistocene isolation across the Indo-Pacific barrier with contemporary overlap in the coral triangle. *BMC Evolutionary Biology*, **11**, 189. doi:10.1186/1471-2148-11-189

Gaither MR, Jones SA, Kelley C, Newman SJ, Sorenson L & Bowen BW (2011b) High connectivity in the deepwater snapper *Pristipomoides filamentosus* (Lutjanidae) across the Indo-Pacific with isolation of the Hawaiian archipelago. *PLoS ONE*, **6**, e28913. doi:10.1371/journal.pone.0028913

Gaither MR, Toonen RJ & Bowen BW (2012) Coming out of the starting blocks: Extended lag time rearranges genetic diversity in introduced marine fishes of Hawai'i. *Proceedings of the Royal Society B: Biological Sciences*, **279**, 3948-3957. doi:10.1098/rspb.2012.1481

Galarza JA, Carreras-Carbonell J, MacPherson E, Turner GF & Rico C (2006) Isolation and characterization of polymorphic microsatellte markers for peacock wrasse (*Symphodus tinca*). *Molecular Ecology Notes*, **6**, 747-749. doi:10.1111/j.1471-8286.2006.01328.x

Galarza JA, Roques S, Carreras-Carbonell J, Macpherson E, Turner GF & Rico C (2007a) Polymorphic microsatellite loci for the cardinal fish (*Apogon imberbis*). *Conservation Genetics*, **8**, 1251-1253. doi:10.1007/s10592-006-9253-3

Galarza JA, Turner GF, MacPherson E, Carreras-Carbonell J & Rico C (2007) Cross-amplification of 10 new isolated polymorphic microsatellite loci for red mullet (*Mullus barbatus*) in striped red mullet (*Mullus surmuletus*). *Molecular Ecology Notes*, **7**, 230-232. doi:10.1111/j.1471-8286.2006.01551.x

Galarza JA, Carreras-Carbonell J, Macpherson E, Pascual M, Roques S, Turner GF & Rico C (2009a) The influence of oceanographic fronts and early-life-history traits on connectivity among littoral fish species. *Proceedings of the National Academy of Sciences*, **106**, 1473-1478. doi:10.1073/pnas.0806804106

Galarza JA, Turner GF, MacPherson E & Rico C (2009b) Patterns of genetic differentiation between two co-occurring demersal species: The red mullet (*Mullus barbatus*) and the striped red mullet (*Mullus surmuletus*). *Candian Journal of Fisheries and Aquatic Sciences*, **66**, 1478-1490. doi:10.1139/F09-098

Galindo HM, Loher T & Hauser L (2011) Genetic sex identification and the potential evolution of sex determination in Pacific halibut (*Hippoglossus stenolepis*). *Marine Biotechnology*, **13**, 1027-1037. doi:10.1007/s10126-011-9366-7

Gao T, Han Z, Zhang X, Luo J, Yanagimoto T & Zhang H (2016) Population genetic differentiation of the black rockfish *Sebastes schlegelii* revealed by microsatellites. *Biochemical Systematics and Ecology*, **68**, 170-177. doi:10.1016/j.bse.2016.07.013

Gao B, Song N, Li Z, Gao T & Liu L (2019a) Population genetic structure of *Nuchequula mannusella* (Perciformes: Leiognathidae) population in the Southern Coast of China inferred from complete sequence of mtDNA *Cytb* gene. *Pakistan Journal of Zoology*, **51**, 1203-1598.

Gao T, Li L, Fang R, Liu G, Wang L, Xu H & Song N (2019) Shallow genetic structure of *Pholis fangi* in Bohai Sea and Yellow Sea inferred from mtDNA control region. *Journal of Ocean University of China*, **18**, 947-952. doi:10.1007/s11802-019-3991-6

Gao T, Yang T, Yanagimoto T & Xiao Y (2019c) Levels and patterns of genetic variation in Japanese whiting (*Sillago japonica*) based on mitochondrial DNA control region. *Mitochondrial DNA Part A*, **30**, 172-183. doi:10.1080/24701394.2018.1467411

Garber AF, Tringali MD & Franks JS (2005) Population genetic and phylogeographic structure of wahoo, *Acanthocybium solandri*, from the western central Atlantic and central Pacific Oceans. *Marine Biology*, **147**, 205-214. doi:10.1007/s00227-004-1533-1

Garcia KK, Touma J, Bravo S, Leiva F, Varags-Chacoff L, Valenzuela A, Datagnan P, Amthauer R, Reyes A & Vidal R (2019) Novel microsatellite markers discovery in Patagonian toothfish (*Dissostichus eleginoides*) using high-throughput sequencing. *Molecular Biology Reports*, **46**, 5525-5530. doi:10.1007/s11033-019-04912-6

García de León FJ, Chikhi L & Bonhomme F (1997) Microsatellite polymorphism and population subdivision in natural populations of European sea bass *Dicentrarchus labrax* (Linnaeus, 1758). *Molecular Ecology*, **6**, 51-62. doi:10.1046/j.1365-1997.t01-1-00151.x

García-De León FJ, Galván-Tirado C, Velasco LS, Silva-Segundo CA, Hernández-Guzmán R, Barriga-Sosa IA, Jaimes PD, Canino M & Cruz Hernández P (2018) Role of oceanography in shaping the genetic structure in the North Pacific hake *Merluccius productus*. *PLoS ONE*, **13**, e0194646. doi:10.1371/journal.pone.0194646

García-Rodríguez F, García-Gasca SA, de la Cruz-Agüero J, Cota-Gómez VM (2011) A study of the population structure of the Pacific sardine *Sardinops sagax* (Jenyns, 1842) in Mexico based on morphometric and genetic analyses. *Fisheries Research*, **107**, 169-176. doi:10.1016/j.fishres.2010.11.002

Gardner MJ, Chaplin JA, Potter I, Fairclough DV & Jackson G (2017) The genetic structure of a marine teleost, *Chrysophrys auratus*, in a large, heterogeneous marine embayment. *Environmental Biology of Fishes*, **100**, 1411-1425. doi:10.1007/s10641-017-0652-8

Garoia F, Guarniero I, Piccinetti C & Tinti F (2004) First microsatellite loci of red mullet (*Mullus barbatus*) and their application to genetic structure analysis of Adriatic shared stock. *Marine Biotechnology*, **6**, 446-452. doi:10.1007/s10126-004-3045-x

Garoia F, Marzola S, Guarniero I, Trentini M & Tinti F (2006) Isolation of polymorphic DNA microsatellites in the common sole *Solea vulgaris*. *Molecular Ecology Notes*, **6**, 144-146. doi:10.1111/j.1471-8286.2005.01169.x

Gharrett AJ, Riley RJ & Spencer PD (2012) Genetic analysis reveals restricted dispersal of northern rockfish along the continental margin of the Bering Sea and Aleutian Islands. *Transactions of the American Fisheries Society*, **141**, 370-392. doi:10.1080/00028487.2012.662419

Ghasemi A & Shadi A (2018) Population structure of *Acanthopagrus latus* from the Northern Persian Gulf and Gulf of Oman based on microsatellite markers. *Turkish Journal of Fisheries and Aquatic Sciences*, **18**, 983-990. doi:10.4194/1303-2712-v18_9_06

Gilbert-Horvath EA, Larson RJ & Garza JC (2006) Temporal recruitment patterns and gene flow in kelp rockfish (*Sebastes atrovirens*). *Molecular Ecology*, **15**, 3801-3815. doi:10.1111/j.1365-294X.2006.03033.x

Glover KA, Skaala Ø, Limborg M, Kvamme C & Torstensen E (2011) Microsatellite DNA reveals population genetic differentiation among sprat (*Sprattus sprattus*) sampled throughout the Northeast Atlantic, including Norwegian fjords. *ICES Journal of Marine Science*, **68**, 2145-2151. doi:10.1093/icesjms/fsr153

Golani D, Azzurro E, Corsini-Foka M, Falautano M, Andolaro F & Bernardi G (2007) Genetic bottlenecks and successful biological invasions: The case of a recent Lessepsian migrant. *Biology Letters*, **3**, 541-545. doi:10.1098/rsbl.2007.0308

Gold JR, Jobity AMC, Saillant E & Renshaw MA (2010) Population structure of carite (*Scomberomorus brasiliensis*) in waters offshore of Trinidad and northern Venezuela. *Fisheries Research*, **103**, 30-39. doi:10.1016/j.fishres.2010.01.009

Gold JR, Saillant E, Cummings NJ & Renshaw MA (2011) Genetic divergence and effective size among lane snapper in U.S. waters of the Western Atlantic Ocean. *North American Journal of Fisheries Management*, **31**, 209-223. doi:10.1080/02755947.2011.568864

Gomes G, Schneider H, Vallinoto M, Santos S, Orti G & Sampaio I (2008) Can *Lutjanus purpureus* (South red snapper) be “legally” considered a red snapper (*Lutjanus campechanus*)? *Genetics and Molecular Biology*, **31**, 372-376.

Gomez-Uchida D & Banks MA (2005) Microsatellite analyses of spatial genetic structure in darkblotched rockfish (*Sebastes crameri*): Is pooling samples safe? *Canadian Journal of Fisheries and Aquatic Sciences*, **62**, 1874-1886. doi:10.1139/f05-084

Gomez-Uchida D (2006) *Spatial and temporal scales of genetic change in two overfished rockfishes*, Oregon State University. Gonzalez EG & Zardoya R (2007) Relative role of life-history traits and historical factors in shaping genetic population structure of sardines (*Sardina pilchardus*). *BMC Evolutionary Biology*, **7**, 197. doi:10.1186/1471-2148-197

Gonzalez EG, Beerli P & Zardoya R (2008) Genetic structuring and migration patterns of Atlantic bigeye tuna, *Thunnus obesus* (Lowe, 1839). *BMC Evolutionary Biology*, **8**, 252. doi:10.1186/1471-2148-8-252

Gonzalez EB, Murakami T, Teshima Y, Yoshioka K, Jeong D-S & Umino T (2009) Paternity testing of wild black rockfish *Sebastes inermis* (brownish type) from the Seto Inland Sea of Japan. *Ichthyological Research*, **56**, 87-91. doi:10.1007/s10228-008-0055-0

Gonzalez EB, Knutsen H, Jorde PE, Glover KA & Bergstad OA (2015) Genetic analyses of ling (*Molva molva*) in the Northeast Atlantic reveal patterns relevant to stock assessments and management advice. *ICES Journal of Marine Science*, **72**, 635-641. doi:10.1093/icesjms/fsu135

González-Wangümert M, Cánovas F, Pérez-Ruzafa A, Marcos C & Alexandrino P (2010) Connectivity patterns inferred from the genetic structure of white seabream (*Diplodus sargus* L.) *Journal of Experimental Marine Biology and Ecology*, **383**, 23-31. doi:10.1016/j.jembe.2009.10.010

González-Wangümert M, Froufe E, Pérez-Ruzafa Á & Alexandrino P (2011) Phylogeographical history of the white seabream *Diplodus sargus* (Sparidae): Implications for insularity. *Marine Biology Research*, **7**, 250-260. doi:10.1080/17451000.2010.499438

González-Wangümert M, Fernández TV, Pérez-Ruzafa A, Giacalone M, D'Anna G & Badalamenti F (2012) Genetic considerations on the introduction of farmed fish in marine protected areas: The case of study of white seabream restocking in the Gulf of Castellammare (Southern Tyrrhenian Sea). *Journal of Sea Research*, **68**, 41-48. doi:10.1016/j.seares.2011.12.005

González-Wangümert M & Pérez-Ruzafa Á (2012) In two waters: Contemporary evolution of lagoonal and marine white seabream (*Diplodus sargus*) populations. *Marine Ecology*, **33**, 337-349. doi:10.111/j.1439-0485.2011.00501.x

Goswami M, Thangaraj K, Chaudhary BK, Bhaskar LVSK, Gopalakrishnan A, Joshi MB, Singh L & Lakra WS (2009) Genetic heterogeneity in the Indian stocks of seahorse (*Hippocampus kuda* and *Hippocampus trimaculatus*) inferred from mtDNA cyctochrome b gene. *Hydrobiologia*, **621**, 213-221. doi:10.1007/s10750-008-9642-3

Gotoh RO, Sekimoto H, Chiba SN & Hanzawa N (2009) Peripatric differentiation among adjacent marine lake and lagoon populations of a coastal fish, *Sphaeramia orbicularis* (Apogonidae, Perciformes, Teleostei). *Genes & Genetic Systems*, **84**, 287-295. doi:10.1266/ggs.84.287

Grant WS, Lecomte F & Bowen BW (2010) Biogeographical contingency and the evolution of tropical anchovies (genus *Cetengraulis*) from temperate anchovies (genus *Engraulis*). *Journal of Biogeography*, **37**, 1352-1362. doi:10.1111/j.1365-2699.2010.02291.

Grant WS, Liu M, Gao T & Yanagimoto T (2012) Limits of Bayesian skyline plot analysis of mtDNA sequences to infer historical demographies in Pacific herring (and other species). *Molecular Phylogenetics and Evolution*, **65**, 203-212. doi:10.1016/j.ympev.2012.06.006

Graves JE & McDowell JR (2006) Genetic analysis of white marlin (*Tetrapturus albidus*) stock structure. *Bulletin of Marine Science*, **79**, 469-482.

Green ME, Appleyard SA, White W, Tracey S, Devloo-Delva F & Ovenden JR (2019) Novel multimarker comparisons address the genetic population structure of silvertip sharks (*Carcharhinus albimarginatus*). *Marine and Freshwater Research*, **70**, 1007-1019. doi:10.1071/MF18296

Guillemaud T, Streiff R, Santos RS, Afonso P, Morato T & Cancela ML (2000) Microsatellite characterization in the rainbow wrasse *Coris julis* (Pisces: Labridae). *Molecular Ecology*, **9**, 629-644. doi:10.1.1.621.6598

Guo W, Wang Z, Wang Y, Zhang Z & Gui J (2005) Isolation and characterization of six microsatellite markers in the large yellow croaker (*Pseudosciaena crocea* Richardson). *Molecular Ecology Notes*, **5**, 369-371. doi:10.1111/j.1471-8286.2005.00930.x

Guo Y, Wang Z, Liu C, Liu L & Liu Y (2007) Isolation and characterization of microsatellite DNA loci from Russell's snapper (*Lutjanus russellii*). *Molecular Ecology Notes*, **7**, 1219-1221. doi:10.1111/j.1471-8286.2007.01837.x

Gwak WS & Nakayama K (2011) Genetic variation and population structure of the Pacific cod *Gadus macrocephalus* in Korean waters revealed by mtDNA and msDNA markers. *Fisheries Science*, **77**, 945-952. doi:10.1007/s12562-011-0403-2

Gwilliam MP, Winkler AC, Potts WM, Santos CV, Sauer WHH, Shaw PW, McKeown NJ (2018) Integrated genetic and morphological data support eco-evolutionary divergence of Angolan and South African populations of *Diplodus hottentotus*. *Journal of Fish Biology*, **92**, 1163-1176. doi:10.1111/jfb.13582

Habib ZA, Jeong D, Myoung J, Kim MS, Jang YS, Shim JS & Lee Y (2011) Population genetic structure and demographic history of the fat greenling *Hexagrammos otakii*. *Genes & Genomics*, **33**, 413-423. doi:10.1007/s13258-011-0059-4

Habib KA, Jeong D, Myoung J-G & Lee Y-H (2015) Population panmixia and the Pleistocene demographic expansion of spotty belly greenling *Hexagrammos agrammus* in the East Sea and Northwest Pacific. *Ocean Science Journal*, **50**, 143-154. doi:10.1007/s12601-015-0011-7

Habib A & Sulaiman Z (2016) Phylogenetic and morphometric relationships between two species of genus *Auxis* from the South China Sea and Java Sea. *Acta Oceanologica Sinica*, **35**, 76-82. doi:10.1007/s13131-016-0915-

Haffray P, Malha R, Sidi MOT, Prista N, Hassan M, Castelnaud G, Karahan-Nomm B, Gamsiz K, Sadek S, Bruant J, Balma P &Bonhomme F (2012) Very high genetic fragmentation in a large marine fish, the meagre *Argyrosomus regius* (Sciaenidae, Perciformes): Impact of reproductive migration, oceanic barriers and ecological factors. *Aquatic Living Resources*, **25**, 173-183. doi:10.1051/alr/2012016

Hamasaki K, Toriya S, Shishidou H, Sugaya T & Kitada S (2010) Genetic effects of hatchery fish on wild populations in red sea bream *Pagrus major* (Perciformes, Sparidae) inferred from a partial sequence of mitochondrial DNA. *Journal of Fish Biology*, **77**, 2123-2136. doi:10.1111/j.1095-8649.2010.02826.x

Han Z, Gao T, Yanagimoto T & Sakurai Y (2008) Deep phylogeographic break among white croaker *Pennahia argentata* (Sciaenidae, Perciformes) populations in North-western Pacific. *Fisheries Science*, **74**, 770-780. doi:10.1111/j.1444-2906.2008.01588.x

Haney RA, Siliman BR & Rand DM (2007) A multi-locus assessment of connectivity and historical demography in the bluehead wrasse (*Thalassoma bifasciatum*). *Heredity*, **98**, 294-302

Harrison HB, Feldheim KA, Jones GP, Mansour H, Perumal S, Williamson DH & Berumen ML (2014) Validation of microsatellite multiplexes for parentage analysis in a coral reef fish (*Lutjanus carponotatus*, Lutjanidae). *Conservation Genetics Resources*, **6**, 803-806. doi:10.1007/s12686-014-0226-4

Hauser L, Adcock GJ, Smith PJ, Bernal-Ramírez JH & Carvalho GR (2002) Loss of microsatellite diversity and low effective population size in an overexploited population of New Zealand snapper (*Pagrus auratus*). *Proceedings of the National Academy of Sciences*, **99**, 18. doi:10.1073/pnas.172242899

Hauser L, Spies I & Loher T (2006) Microsatellite screening in Pacific halibut (*Hippoglossus stenolepis*) and a preliminary examination of population structure based on observed DNA variation. *International Pacific Halibut Commission Scientific Report*, **81**.

Hauser L, Newton L, LeClair L & Buckley RM (2007) Genetic identification of progeny of reef-resident brown rockfish (*Sebastes auriculatus*). *Biology Assessment and Management of North Pacific Rockfishe (eds Heifetz J, Dicosimo J, Gharrett AJ et al.)*, 99-119.

Healey AJE, Gouws G, Fennessy ST, Kuguru B, Sauer WHH, Shaw PW & McKeown NJ (2018b) Genetic analysis reveals harvested *Lethrinus nebulosus* in the Southwest Indian Ocean comprise two cryptic species. *ICES Journal of Marine Science*, **75**, 1465-1472. doi:10.1093/icesjms/fsx245

Healey AJE, McKeown NJ, Taylor AL, Provan J, Sauer W, Gouws G & Shaw PW (2018a) Cryptic species and parallel genetic structuring in Lethrinid fish: Implications for conservation and management in the southwest Indian Ocean. *Ecology and Evolution*, **8**, 2182-2195. doi:10.1002/ece3.377

Heist EJ, Jenkot JL, Keeney DB, Lane RL, Moyer GR, Reading BJ & Smith NL (2003) Isolation and characterization of polymorphic microsatellite loci in nurse shark (*Ginglymostoma cirratum*). *Molecular Ecology Notes*, **3**, 59-61. doi:10.1046/j.1471-8286.2003.00348.x

Heist EJ, Sipiorski J & Tricas TC (2008) Development of DNA microsatellite markers in the multiband butterflyfish (*Chaetodon multicinctus*). *Molecular Ecology Resources*, **8**, 1518-1519. doi:10.1111/j.1755-0998.2008.02181.

Helyar S, Sacchi C, Coughlan J & Mariani S (2010) Novel microsatellite loci for a deep sea fish (*Macrourus berglax*) and their amplification in other grenadiers (Gadiformes: Macrouridae). *Conservation Genetics Resources*, **2**, 1-4. doi:10.1007/s12686-009-9049-0

Helyar S, Corscia I, Sala-Bozano M & Mariani S (2011) New microsatellite loci for the longnose velvet dogfish (*Centroselachus crepidater*) (Squaliformes: Somniosidae) and other deep sea sharks. *Conservation Genetics Resources*, **3**, 173-176. doi:10.1007/s12686-010-9316-0

Hemmer-Hansen J, Nielsen EEG, Grønkjær P & Loeschcke V (2007) Evolutionary mechanisms shaping the genetic population structure of marine fishes; lessons from the European flounder (*Platichthys flesus* L.). *Molecular Ecology*, **16**, 3104-3118. doi:10.1111/j.1365-294X.2007.03367.x

Henriques R, McKeown NJ & Shaw PW (2012a) Isolation of 12 microsatellite markers for geelbeck (*Atractoscion aequidens* (Cuvier, 1860), Sciaenidae), an overexploited marine fish. *Conservation Genetics Resources*, **4**, 85-87. doi:10.1007/s12686-011-9480-x

Henriques R, Potts WM, Sauer WHH & Shaw PW (2012b) Evidence of deep genetic divergence between populations of an important recreational fishery species, *Lichia amia* L. 1758, around southern Africa. *African Journal of Marine Science*, **34**, 585-591. doi:10.2989/1814232X.2012.749809

Henriques R, Potts WM, Santos CV, Sauer WHH & Shaw PW (2014) Population connectivity and Phylogeography of a coastal fish, *Atractoscion aequidens* (Sciaenidae), across the Benguela Current region: Evidence of an ancient vicariant event. *PLoS ONE*, **9**, e87907. doi:10.1371/journal.pone.0087907

Henriques R, Potts WM, Sauer WHH & Shaw PW (2015) Incipient genetic isolation of a temperate migratory coastal sciaenid fish (*Argyrosomus inodorus*) within the Benguela Cold Current system. *Marine Biology Research*, **11**, 423-429. doi:10.1090/17451000.2014.952309

Henriques R, Nielsen ES, Durholtz D, Japp D & von der Heyden S (2017) Genetic population sub-structuring of kingklip (*Genypterus capensis* – Ophidiidiae), a commercially exploited demersal fish off South Africa. *Fisheries Research*, **187**, 86-95. doi:10.1016/j.fishres.2016.11.007

Henriques R, Potts WM, Santos CV, Sauer WHH & Shaw PW (2018) Population connectivity of an overexploited coastal fish, *Argyrosomus coronus*, (Sciaenidae), in an ocean-warming hotspot. *African Journal of Marine Science*, **40**, 13-24. doi:10.2989/1814232X.2018.1434090

Hepburn RI, Sale PF, Dixon B & Heath DD (2009) Genetic structure of juvenile cohorts of bicolor damselfish (*Stegastes partitus*) along the Mesoamerican barrier reef: Chaos through time. *Coral Reefs*, **28**, 277-288. doi:10.1007/s00338-008-0423-2

Hess JE, Vetter RD & Moran P (2011) A steep genetic cline in yellowtail rockfish, *Sebastes flavidus*, suggests regional isolation across the Cape Mendocino faunal break. *Canadian Journal of Fisheries and Aquatic Sciences*, **68**, 89-104. doi:10.1139/F10-131

Hickey AJR, Lavery SD, Hannan DA, Baker CS & Clements KD (2009) New Zealand triplefin fishes (family Tripterygiidae): contrasting population structure and mtDNA diversity within a marine species flock. *Molecular Ecology*, **18**, 680-696. doi:10.1111/j.1365.294X.2008.04052.x

Ho ALFC, Pruett CL & Lin J (2012) Population genetic structure, coloration, and morphometrics of yellowhead jawfish *Opistognathus aurifrons* (Perciformes: Opistognathidae) in the Caribbean region. *Marine Ecology Progress Series*, **444**, 275-287. doi:10.3354/meps09435

Hoarau G, Rijnsdorp AD, Van Der Veer HW, Stam WG & Olsen JL (2002) Population structure of plaice (*Pleuronectes platessa* L.) in northern Europe: Microsatellites revealed large-scale spatial and temporal homogeneity. *Molecular Ecology*, **11**, 1165-1176. doi:10.1046/j.1365-294X.2002.01515.x

Hoarau G, AM-T Piquet van der Veer HW, Rijnsdorp AD, Stam WT, Olsen JL (2004) Population structure of plaice (*Pleuronectes platessa* L.) in northern Europe: a comparison of resolving power between microsatellites and mitochondrial DNA data. *Journal of Sea Research*, **51**, 183-190. doi:10.1016/j.seares.2003.12.002

Hoffman EA, Arguello JR, Kolm N, Berglund A & Jones AG (2004) Eleven polymorphic microsatellite loci in a coral reef fish, *Pterapogon kauderni*. *Molecular Ecology Notes*, **4** 342-344. doi:10.1111/j.1471-8286.2004.00691.

Hoffman EA, Kolm N, Berglund A, Arguello JR & Jones AG (2005) Genetic structure in the coral-reef-associated Banggai cardinalfish, *Pterapogon kauderni*. *Molecular Ecology*, **14**, 1367-1375. doi:10.1111/j.1365-294X.2005.02538.x

Hollenbeck CM, Portnoy DS, Saillant E & Gold JR (2015) Population structure of red snapper (*Lutjanus campechanus*) in U.S. waters of the western Atlantic Ocean and the northeastern Gulf of Mexico. *Fisheries Research*, **172**, 17-25. doi:10.1016/j.fishres.2015.06.02

Hoolihan JP, Anandh P & van Herwerden L (2006) Mitochondrial DNA analyses of narrow-barred Spanish mackerel (*Scomberomorus commerson*) suggest a single genetic stock in the ROPME sea area (Arabian Gulf, Gulf of Oman, and Arabian Sea). *ICES Journal of Marine Science*, **63**, 1066-1074. doi:10.1016/j.icesjms.2006.03.012

Horne JB, McIlwain JL & van Herwerden L (2010) Isolation of 15 new polymorphic microsatellite markers from the blue-spine unicornfish *Naso unicornis*. *Conservation Genetics Resources*, **2**, 191-194. doi:10.1007/s12686-009-9129-1

Horne JB, van Herwerden L, Abellana S & McIlwain JL (2013) Observations of migrant exchange and mixing in a coral reef fish metapopulation link scales of marine population connectivity. *Journal of Heredity*, **104**, 532-546. doi:10.1093/jhered/est02

Hu J, Zhu XP, Luo J, Yin SW, Peng YH, Hu YL & Zhu F (2013) Development and characterization of microsatellite loci in a threatened marine fish, *Cheilinus undulatus* (humphead wrasse). *Genetics and Molecular Research*, **12**, 2633-2636. doi:10.4238/2013.July.30.2

Huang W, Chang J, Liao C, Tawa A, Iizuka Y, Liao T & Shiao J (2018) Pelagic larval duration, growth rate, and population genetic structure of the tidepool snake moray *Uropterygius micropterus* around the southern Ryukyu Islands, Taiwan, and the central Philippines. *PeerJ*, **6**, e4741. doi:10.7717/peerj.4741

Hull KL, Asbury TA, da Silva C, Dicken M, Veríssimo A, Farrell ED, Mariani S, Mazzoldi C, Marino IAM, Zane L, Maduna SN & Bester-van der Merwe AE (2019) Strong genetic isolation despite wide distribution in a commercially exploited coastal shark. *Hydrobiologica*, **838**, 121-137. doi:10.1007/s10750-019-02982-8

Huyghe F & Kochzius M (2018) Sea surface currents and geographic isolation shape the genetic population structure of a coral reef fish in the Indian Ocean. *PLoS ONE*, **13**, e0193825. doi:10.1371/journal.pone.0193825

Hyde JR, Kimbrell CA, Budrick JE, Lynn EA & Vetter RD (2008) Cryptic speciation in the vermilion rockfish (*Sebastes miniatus*) and the role of bathymetry in the speciation process. *Molecular Ecology*, **17**, 1122-1136. doi:10.1111/j.1365-294X.2007.03653.x

Hyde JR & Vetter RD (2009) Population genetic structure in the redefined vermilion rockfish (*Sebastes miniatus*) indicates limited larval dispersal and reveals natural management units. *Canadian Journal of Fisheries and Aquatic Sciences*, **66**, 1569-1581. doi:10.1139/F09-10

Im YJ, Jo HS, Ji HS, Myoung SH & Kim JK (2017) Geographic variations of the mottled skate, *Beringraja pulchra* (Liu, 1932) (Rajidae) in the Yellow and East seas based on molecular and morphometric data. *Journal of Applied Ichthyology*, **33**, 950-956. doi:10.1111/jai.13408

Itoi S, Odaka J, Noguchi S, Noda T, Yuasa K, Muraki T, Tanabe T, Takai N, Yoshihara K & Sugita H (2011) Genetic homogeneity between adult and juvenile populations of *Scombrops gilberti* (Percoid, Scombropidae) in the Pacific Ocean off the Japanese Islands. *Fisheries Science*, **77**, 975-981. doi:10.1007/s12562-011-0414-z

Iwamoto K, Takemura A, Yoshino T & Imai H (2009) Molecular ecological study of *Siganus spinus* and *S. guttatus* from Okinawan waters based on mitochondrial DNA control region sequences. *Journal of Oceanography*, **65**, 103-112. doi:10.1007/s10872-009-0010-3

Iwamoto K, Chang C, Takemura A & Imai H (2012) Genetically structured population and demographic history of the goldlined spinefoot *Siganus guttatus* in the northwestern Pacific. *Fisheries Science*, **78**, 249-257. doi:10.1007/s12562-011-0455-3

Jackson AM, Semmens BX, de Mitcheson YS, Nemeth RS, Heppell SA, Bush, PG, Aguilar-Perera A, Clayton JAB, Calossa MC, Sealey KS, Schärer MT, Bernardi G (2014) Population structure and Phylogeography in Nassau grouper (*Epinephelus striatus*), a mass-aggregating marine fish. *PLoS ONE*, **9**, e97508. doi:10.1371/journal.pone.009750

Jansson E, Quintela M, Dahle G, Albretsen J, Knutsen H, André C, Strand Å, Mortensen S, Taggart JB, Karlsbakk E, Kvamme BO & Glover KA (2017) Genetic analysis of goldsinny wrasse reveals evolutionary insights into population connectivity and potential evidence of inadvertent translocation via aquaculture. *ICES Journal of Marine Science*, **74**, 2135-2147. doi:10/1093/icesjms/fsx04

Jean C, Lee S, Chen C & Hu C (1998) Variation in mitochondrial DNA sequences of black porgy, *Acanthopagrus schlegeli*, in the coastal waters of Taiwan. *Zoological Studies*, **37**, 22-30.

Jean CT, Lee SC, Liu CW & Tseng MC (2006) Isolation and characterization of eight microsatellite loci from Picnic seabream (*Acanthopagrus berda*). *Molecular Ecology Notes*, **6**, 1269-1271. doi:10.1111/j.1471-8286.2006.01511.x

Jeong DS, Umino T, Kuroda K, Hayashi M, Nakagawa H, Kang JC, Morishima K & Arai K (2003) Genetic divergence and population structure of black sea bream *Acanthopagrus schlegeli* inferred from microsatellite analysis. *Fisheries Science*, **69**, 896-902. doi:10.1046/j.1444-2906.2003.00705.x

Johnson PB, Martin KL, Vandergon TL, Honeycutt RL, Burton RS & Fry A (2009) Microsatellite and mitochondrial genetic comparisons between northern and southern populations of California grunion (*Leuresthes tenuis*). *Copeia*, **3**, 465-474. doi:10.1643/CI-07-253

Jones AG & Avise JC (1997) Microsatellite analysis of maternity and the mating system in the Gulf pipefish *Syngnathus scovelli*, a species with male pregnancy and sex-role reversal *Molecular Ecology*, **6**, 203-213. doi:10.1046/j.1365-294X.1997.00173.x

Jones AG, Kvarnemo C, Moore GI, Simmons LW & Avise JC (1998a) Microsatellite evidence for monogamy and sex-biased recombination in the Western Australian seahorse *Hippocampus angustus*. *Molecular Ecology*, **7**, 1497-1505. doi:1-.1046/j.1365-294X.1998.00481.x

Jones AG, Östlund-Nilsson S & Avise JC (1998b) A microsatellite assessment of sneaked fertilizations and egg thievery in the fifteenspine stickleback. *Evolution*, **52**, 848-858. doi:10.1111/j.1558-5646.1998.tb03709

Jones AG, Rosenqvist G, Berglund A & Avise JC (1999) The genetic mating system of a sex-role-reversed pipefish (*Syngnathus typhle*): a molecular inquiry. *Behavioral Ecology and Sociobiology*, **46**, 357-365. doi:10.1007/s002650050630

Jones ME & Barber PH (2005) Characterization of microsatellite loci for the detection of temporal genetic shifts within a single cohort of the brown demoiselle, *Neopomacentrus filamentosus*. *Molecular Ecology Notes*, **5**, 834-836. doi:10.1111/j.1471-8286.2005.01079.x

Jones DB, Jerry DR, McCormick MI & Bay LK (2008) Development of nine microsatellite markers for *Pomacentrus amboinensis*. *Molecular Ecology Resources*, **8**, 1332-1334. doi:10.1111/j.1755-0998.2008.02295.x

Jónsdóttir ÓDB, Schregel J, Hagen SB, Tobiassen C, Aarnes SG & Imsland AKD (2018) Population genetic structure of lumpfish along the Norwegian coast: Aquaculture implications. *Aquaculture International*, **26**, 49-60. doi:10.1007/s10499-017-0194-2

Jørgensen HBH, Hansen MM, Bekkevold D, Ruzzante DE & Loeschcke V (2005a) Marine landscapes and population genetic structure of herring (*Clupea harengus* L.) in the Baltic Sea. *Molecular Ecology*, **14**, 3219-3234. doi:10.1111/j.1365-294X.2005.02658.x

Jørgensen HBH, Hansen MM & Loeschcke V (2005) Spring-spawning herring (*Clupea harengus* L.) in the southwestern Baltic Sea: Do they form genetically distinct spawning waves? *ICES Journal of Marine Science*, **62**, 1065-1075. doi:10.1016/j.icesjms.2005.04.007

Jue NK (2010)*The role of larval dispersal in the population genetics and ecology of gag,* Mycteroperca microlepis*, in the Gulf of Mexico*, Florida State University.

Kang J, Yang S, Moon T, Park J & Choi T (2013) Development of microsatellite markers for the kelp grouper *Epinephelus bruneus* by 454 pyrosequencing and transfer to related species. *Genetics and Molecular Research*, **12**, 5485-5493. doi:10.4238/2013.November.13.1

Kaouèche M, Bahri-Sfar L, Hammami I & Ben Hassine OK (2013) Morphological and genetic variations of *Diplodus vulgaris* along the Tunisian coasts. *Cybium*, **37**, 111-120.

Karaiskou N, Triantafyllidis A, Katsares V, Abatzopoulos TJ & Triantaphyllidis C (2009) Microsatellite variability of wild and farmed populations of *Sparus aurata*. *Journal of Fish Biology*, **74**, 1816-1825. doi:10.1111/j.1095-8649.2009.02186.x

Karl SA, Castro ALF & Garla RC (2012) Population genetics of the nurse shark (*Ginglymostoma cirratum*) in the western Atlantic. *Marine Biology*, **159**, 489-498. doi:10.1007/s00227-011-1828-y

Karlsson S & Mork J (2005) Deviation from Hardy-Weinberg equilibrium, and temporal instability in allele frequencies at microsatellite loci in a local population of Atlantic cod. *ICES Journal of Marine Science*, **62**, 1588-1596. doi:10.1016/j.icesjms.2005.05.009

Karlsson S, Renshaw MA, Rexroad III CE & Gold JR (2008a) Microsatellite primers for red drum (*Sciaenops ocellatus*). *Fishery Bulletin*, **106**, 476-482. doi:10113/36466

Karlsson S, Renshaw MA, Rexroad III CE & Gold JR (2008b) PCR primers for 100 microsatellites in red drum (*Sciaenops ocellatus*). *Molecular Ecology Resources*, **8**, 393-398. doi:10.1111/j.1471-8286.2007.01969.x

Karlsson S, Saillant E & Gold JR (2009) Population structure and genetic variation of lane snapper (*Lutjanus synagris*) in the northern Gulf of Mexico. *Marine Biology*, **156**, 1841-1855. doi:10.1007/s00227-009-1217-y

Kasapidis P & Magoulas A (2008) Development and application of microsatellite markers to address the population structure of the horse mackerel *Trachurus trachurus*. *Fisheries Research*, **89**, 132-135. doi:10.1016/j.fishres.2007.09.015

Keeney DB, Heupel MR, Huetter RE & Heist EJ (2005) Microsatellite and mitochondrial DNA analyses of the genetic structure of blacktip shark (*Carcharhinus limbatus*) nurseries in the northwestern Atlantic, Gulf of Mexico, and Caribbean Sea. *Molecular Ecology*, **14**, 1911-1923. doi:10.1111/j.1365-294X.2005.02549.x

Kempter J, Kielpinski M, Panicz R, Prüffer K, Keszka S (2017) Development of the method for identification of selected populations of torpedo scad, *Megalaspis cordyla* (Linnaeus, 1758), using microsatellite DNA analyses. CELFISH project – Part 4. *Food Chemistry*, **221**, 944-949. doi:10.1016/j.foodchem.2016.11.070

Kennington WJ, Keron PW, Harvey ES, Wakefield CB, Williams AJ, Halafihi T & Newman SJ (2017) High intra-ocean, but limited inter-ocean genetic connectivity in populations of the deep-water oblique-banded snapper *Pristipomoides zonatus* (Pisces: Lutjanidae). *Fisheries Research*, **193**, 242-249. doi:10.1016/j.fishres.2017.04.015

Kijewska A, Więcaszek B & Kijewski T (2011) Analysis of population and taxonomical structure of Atlantic cod, *Gadus morhua* (Actinopterygii: Gadiformes: Gadidae) from the Baltic Sea with use of microsatellite DNA. *Acta Ichthyologica et Piscatoria*, **41**, 307-314. doi:10.3750/AIP2011.41.4.07

Kim WJ, Kim KK, Lee JH, park DW, Park JY & Lee JY (2003) Isolation and characterization of polymorphic loci in the olive flounder (*Paralichthys olivaceus*). *Molecular Ecology Notes*, **3**, 491-493. doi:10.1046/j.1471-8286.2003.00524.x

Kim JK, Park JY & Kim YS (2006) Genetic diversity, relationships and demographic history of three geographic populations of *Ammodytes personatus* (Ammodytidae) from Korea inferred from mitochondrial DNA control region and 16S rRNA sequence data. *Korean Journal of Genetics*, **28**, 343-351.

Kim SG, Morishima K & Ara K (2007) Isolation and characterization of polymorphic microsatellite DNA markers in the brown sole, *Pleuronectes herzensteini*. *Molecular Ecology Notes*, **7**, 79-81. doi:10.1111/j.1471-8286.2006.01535.x

Kim S-G, Morishima K & Arai K (2009a) Cross-species amplification of microsatellite markers for the brown sole in the family Pleuronectidae. *Fisheries Science*, **75**, 1103-1107. doi:10.1007/s12562-009-0130-0

Kim W, Kang J, Nam B, Kong H, Park E, Lee J, Kim K, Kim Y, Han H, Choi T & Cheong J (2009b) Development of 52 new polymorphic microsatellite markers for the olive flounder, *Paralichthys olivaceus*. *Molecular Ecology Resources*, **9**, 839-842. doi:10.1111/j.1755-0998.2009.02287.x

Kim WJ, Kim KK, Kim YK, Shin EH & Kim SG (2010a) Isolation and characterization of 20 polymorphic microsatellite loci in the black seabream, *Acanthopagrus schlegeli*. *Molecular Ecology Resources*, **10**, 404-408.

Kim M, An H & Choi K (2010b) Genetic characteristics of Pacific cod populations in Korea based on microsatellite markers. *Fisheries Science*, **76**, 595-603. doi:10.1007/s12562-010-0249-z

Kim W, Kim K, Han H, Nam B, Kim Y, Kong H, Noh J & Yoon M (2010c) Population structure of the olive flounder (*Paralichthys olivaceus*) in Korea inferred from microsatellite marker analysis. *Journal of Fish Biology*, **76**, 1958-1971. doi:10.1111/j.1095-8649.2010.02638.x

Kim S, Morishima K & Arai K (2010) Genetic structure of wild brown sole inferred from mitochondrial DNA analysis. *Animal Cells and Systems*, **14**, 197-206. doi:10.1080/19768354.20210.50626

Kinitz T, Quack M, Paulus M, Veith M, Bergek S, Strand J, Tuvikene A, Soirinsuo A & Hochkirch A (2013) Strong isolation-by-distance in the absence of genetic population structure in the eelpout (*Zoarces viviparus*, Linnaeus 1758). *Ecological Indicators*, **27**, 116-122. doi:10.1016/j.ecolind.2012.12.001

Kitanishi S, Fujiwara A, Hori M, Fuji T & Hamaguchi M (2014) Isolation and characterisation of 23 microsatellite markers for marbled sole, *Pleuronectes yokohamae*. *Conservation Genetics Resources*, **6**, 951-953. doi:10.1007/s12686-014-0252-2

Klein M, Teixeira S, Assis J, Serrão EA, Concalves J & Borges R (2016) High interannual variability in connectivity and genetic pool of a temperate clingfish matches oceanographic transport predictions. *PLoS ONE*, **11**, e0165881. doi:10.1371/journal.pone.0165881

Knutsen H, Jorde PE, André C & Stenseth NCHR (2003) Fine-scaled geographical population structuring in a highly mobile marine species: the Atlantic cod. *Molecular Ecology*, **12**, 385-394. doi:10.1046/j.1365-294X.2003.01750.x

Knutsen H, Fiani D, Sannæes H & Hoelzel AR (2007) Isolation and characterization of microsatellite loci in a marine fish species, the tusk (*Brosme brosme*). *Molecular Ecology Notes*, **7**, 851-853. doi:10.1111/j.1471-8286.2007.01726.x

Knutsen H, Catarino D, Sannæs & Stefanni S (2009) Development of eleven microsatellite loci in the deep-sea black scabbardfish (*Aphanopus carbo*). *Conservation Genetics Resources*, **1**, 89. doi:10.1007/s12686-009-9021-z

Knutsen H, Jorde PE, Gonzalez EB, Robalo J, Albretsen J & Almada V (2013) Climate change and genetic structure of leading edge and rear end populations in a northwards shifting marine fish species, the corkwing wrasse (*Symphodus melops*). *PLoS ONE*, **8**, e67492. doi:10.1371/journal.pone.0067492

Kochzius M & Blohm D (2005) Genetic population structure of the lionfish *Pterois miles* (Scorpaenidae, Pteroinae) in the Gulf of Aqaba and northern Red Sea. *Gene*, **347**, 295-301. doi:10.1016/j.gene.2004.12.03

Koedprang W, Na-Nakorn U, Nakajima M & Taniguchi N (2007) Evaluation of genetic diversity of eight grouper species *Epinephelus* spp. Based on microsatellite variations. *Fisheries Science*, **73**, 227-236. doi:10.1111/j.1444-2906.2007.01328.

Kojima S, Moku M & Kawaguchi K (2009) Genetic diversity and population structure of three dominant myctophid fishes (*Diaphus theta*, *Stenobrachius leucopsarus*, and *S. nannochir*) in the North Pacific Ocean. *Journal of Oceanography*, **65**, 187-193. doi:10.1007/s10872-009-0018-8

Kokita T, Matsuoka K, Tominaga O & Seikai T (2006) Isolation and characterization of highly polymorphic microsatellite loci in a pelagic Spawning reef fish *Hypodytes rubripinnis*. *Molecular Ecology Notes*, **6**, 210-211. doi:10.1111/j.1471-8286.2005.01195.x

Kousteni V, Kasapidis P, Kotoulas G & Megalofonou (2015) Strong population genetic structure and contrasting demographic histories for the small-spotted catshark (*Scyliorhinus canicular*) in the Mediterranean Sea. *Heredity*, **114**, 333-343.

Kousteni V, Kasapidis P, Kotoulas G & Megalofonou P (2016) Evidence of high genetic connectivity for the longnose spurdog *Squalus blainville* in the Mediterranean Sea. *Mediterranean Marine Science*, **17**, 371-383. doi:10.12681/mms.1222

Kumagai K, Barinova AA, Nakajima M & Taniguchi N (2004) Genetic diversity between Japanese and Chinese threeline grunt (*Parapristipoma trilineatum*) examined by microsatellite DNA markers. *Marine Biotechnology*, **6**, 221-228. doi:10.1007/s10126-003-0006-8

Kumar G, Kunal SP, Menezes MR & Meena RM (2012a) Three genetic stocks of frigate tuna *Auxis thazard thazard* (Lacepede, 1800) along the Indian coast revealed from sequence analyses of mitochondrial DNA D-loop region. *Marine Biology Research*, **8**, 992-1002. doi:10.1080/17451000.2012.702913

Kumar G, Kunal SP, Menezes MR & Meena RM (2012b) Single genetic stock of kawakawa *Euthynnus affinis* (Cantor, 1849) along the Indian coast inferred from sequence analyses of mitochondrial DNA D-loop region. *Conservation Genetics*, **13**, 1119-1131. doi:10.1007/s10592-012-0359-5

Kunal SP, Kumar G, Menezes MR & Meena RM (2013) Mitochondrial DNA analysis reveals three stocks of yellowfin tuna *Thunnus albacares* (Bonnaterre, 1788) in Indian waters. *Conservation Genetics*, **14**, 205-213. doi:10.1007/s10592-013-0445-3

Kuriiwa K, Chiba SN, Motomura H & Matsuura K (2014) Phylogeography of blacktip grouper, *Epinephelus fasciatus* (Perciformes: Serranidae), and influence of the Kuroshio Current on cryptic lineages and genetic population structure. *Ichthyological Research*, **61**, 361-374. doi:10.1007/s10228-014-0408-9

Lage CR & Kornfield I (1999) Isolation and characterization of microsatellite loci in Atlantic haddock (*Melanogrammus aeglefinus*). *Molecular Ecology*, **8**, 1355-1357. doi:10.1046/j.1365-294X.1999.00701_3.x

Lait LA, Marshall HD & Carr SM (2018) Phylogeographic mitogenomics of Atlantic cod *Gadus morhua*: variation in and among trans-Atlantic, trans-Laurentian, Northern cod, and landlocked fjord populations. *Ecology and Evolution*, **8**, 6420-6437. doi:10.1002/ece3.3873

Lane HS, Symonds JE & Ritchie PA (2016) The phylogeography and population genetics of *Polyprion oxygeneios* based on mitochondrial DNA sequences and microsatellite DNA markers. *Fisheries Research*, **174**, 19-29. doi:10.1016/j.fishres.2015.08.009

Larson S, Christiansen J, Griffing D, Ashe J, Lowry D & Andrews K (2011) Relatedness and polyandry of sixgill sharks, *Hexanchus griseus*, in an urban estuary. *Conservation Genetics*, **12**, 679-690. doi:10.1007/s10592-010-0174-9

Larsson LC, Laikre L, André C, Dahlgren TG & Ryman N (2010) Temporally stable genetic structure of heavily explited Atlantic herring (*Clupea harengus*) in Swedish waters. *Heredity*, **104**, 40-51.

Lawton RJ, Pratchett MS & Bay LK (2010) isolation and characterization of 29 microsatellite loci for studies of population connectivity in the butterflyfishes *Chaetodon trifascialis* and *Chaetodon lunulatus*. *Conservation Genetics Resources*, **2**, 209-213. doi:10.1007/s12686-009-9138-0

Lawton RJ, Messmer V, Pratchett MS & Bay LK (2011) High gene flow across large geographic scales reduces extinction risk for a highly specialised coral feeding butterflyfish. *Molecular Ecology*, **20**, 3584-3598. doi:10.1111/j.1365-294X.2011.05207.x

Le Port A, Montgomery JC, Smith ANH, Croucher AE, McLeod IM & Lavery SD (2017) Temperate marine protected area provides recruitment subsidies to local fisheries. *Proceedings of the Royal Society B: Biological Sciences*, **284**, 20171300. doi:10.1098/rspb.2017.1300

LeClair LL, Young SF & Shaklee JB (2006) Allozyme and microsatellite DNA analyses of lingcod from Puget Sound, Washington, and adjoining waters. *Transactions of the American Fisheries Society*, **135**, 1631-1643. doi:10.1577/T05-116.1

Lee S-J, Lee S-G & Gwak W-S (2012) Population genetic structure and genetic variability of the marbled sole *Pleuronectes yokohamae* on the coast of Gyeongsangnam-do, Korea. *Animal Cells and Systems*, **16**, 498-505. doi:10.1080/19768354.2012.702683

Lemaire C, Versini J & Bonhomme F (2005) Maintenance of genetic differentiation across a transition zone in the sea: Discordance between nuclear and cytoplasmic markers. *Journal of Evolutionary Biology*, **18**, 70-80. doi:10.1111/j.1420-9101.2004.00828.x

Leray M, Beldade R, Holbrook SJ, Schmitt RJ, Planes S & Bernardi G (2010) Allopatric divergence and speciation in coral reef fish: The three spot dascyllus, *Dascyllus trimaculatus*, species complex. *Evolution*, **64**, 1218-1230. doi:10.1111/j.1558-5646.2009.00917.x

Lévy-Hartmann L, Roussel V, Letourneur Y & Sellos DY (2012) Global and New Caledonian patterns of population genetic variation in the deep-sea splendid alfonsino, *Beryx splendens*, inferred from mtDNA. *Genetica*, **139**, 1349-1365. doi:10.1007/s10709-012-9628-y

Li S, Xu D, Lou B, Xu H, You F, Zhan W & Geng Z (2011) Genetic differentiation in Japanese flounder in the Yellow Sea and East China Sea by amplified fragment length polymorphism (AFLP) and mitochondrial DNA markers. *African Journal of Biotechnology*, **10**, 12833-12839. doi:10.5897/AJB11.1231

Li W, Zhong J & Wang Y (2013a) Genetic diversity and population structure of two lancelets along the coast of china. *Zoological Science*, **30**, 83-91. doi:10.2108/zsj.30.83

Li Y, Han Z, Song N & Gao T (2013b) New evidence to genetic analysis of small yellow croaker (*Larimichthys polyactis*) with continuous distribution in China. *Biochemical Systematics and Ecology*, **50**, 331-338. doi:10.1016/j.bse.2013.05.003

Li QH, Li ZB, Dai G, Chen XJ, Chen LN, Cao YY, Shangguan JB & Ning YF (2013c) Characterization of new microsatellite markers of *Siganus fuscescens* (Siganidae). *Genetics and Molecular Research*, **12**, 2751-2754. doi:10.4238/2013.July.30.12

Li Q, Li Z, Dai G, Cao Y, Chen X, Chen L, Shangguan J & Ning Y (2014a) Isolation and characterization of eleven microsatellite loci in the marbled rockfish, *Sebastiscus marmoratus* (Scorpaenidae). *Conservation Genetics Resources*, **6**, 53-55. doi:10.1007/s12686-013-001-y

Li Y, Chen G, Yu J, Wu S, Xiong D, Li X, Cui K & Li Y (2014b) Population genetics of *Thamnaconus hypargyreus* (Tetraodontiformes: Monacanthidae) in the South China Sea. *Mitochondrial DNA Part A*, **27**, 798-805. doi:10.3109/19401736.2014.919451

Li Y, Gao T, Zhou Y & Lin L (2019) Spatial genetic subdivision among populations of *Pampus chinensis* between China and Pakistan: Testing the barrier effect of the Malay Peninsula. *Aquatic Living Resources*, **32**, 8. doi:10.1051/alr/2019004

Limborg MT, Pedersen JS, Hemmer-Hansen J, Tomkiewicz J & Bekkevold D (2009) Genetic population structure of European sprat *Sprattus sprattus*: Differentiation across a steep environmental gradient in a small pelagic fish. *Marine Ecology Progress Series*, **379**, 213-224. doi:10.3354/meps07889

Limborg MT, Hnel R, Debes PV, Ring AK, André C, Tsigenopoulos CS & Bekkevold D (2012) Imprints from genetic drift and mutation imply relative divergence times across marine transition zones in a pan-European small pelagic fish (*Sprattus sprattus*). *Heredity*, **109**,96-107.

Lin L, Zhu L, Liu S, Su Y & Zhuang Z (2011) Polymorphic microsatellite loci for the Japanese anchovy *Engraulis japonicus* (Engraulidae). *Genetics and Molecular Research*, **10**, 764-768. doi:10.4238/vol10-2gmr1085

Liu J, Gao T, Zhuang Z, Jin X, Yokogawa K & Zhang Y (2006) Late Pleistocene divergence and sugsequent population expansion of two closely related fish species, Japanese anchovy (*Engraulis japonicus*) and Australian anchovy (*Engraulis australis*). *Molecular Phylogenetics and Evolution*. **40**, 712-723. doi:10.1016/j.ympev.2006.04.019

Liu YG, Liu LX, Li ZX, Gao AY, Lin H, Li BF & Wang DF (2007) Development of polymorphic microsatellite from RAPD bands of black seabream *Acanthopagrus schlegeli*. *Molecular Ecology Notes*, **7**, 1178-1180. doi:10.1111/j.1471-8286.2007.01823.x

Liu YG, Lao BL, Liu LX, Wang L & Lin H (2008) Isolation and characterization of polymorphic microsatellite loci from RAPD product in half-smooth tongue sole (*Cynoglossus semilaevis*) and a test of cross-species amplification. *Molecular Ecology Resources*, **8**, 202-204. doi:10.1111/j.1471-8286.2007.01923.x

Liu M, Lu ZC, Gao TX, Yanagimoto T & Sakurai Y (2010a) Remarkably low mtDNA control-region diversity and shallow population structure in Pacific cod *Gadus macrocephalus*. *Journal of Fish Biology*, **77**, 1071-1082. doi:10.1111/j.1095-8649.2010.02743.x

Liu SV, Wang C, Shiao J & Dai C (2010b) Population connectivity of neon damsel, *Pomacentrus coelestis*, inferred from otolith microchemistry and mtDNA. *Marine and Freshwater Research*, **61**, 1416-1424. doi:1o.1071/MF10079

Liu J & Avise JC (2011) High degree of multiple paternity in the viviparous shiner perch, *Cymatogaster aggregate*, a fish with long-term female sperm storage. *Marine Biology*, **158**, 893-901. doi:10.1007/s00227-010-1616-0

Liu Y, Guo Y, Liu S, Li J & Ye N (2011) Genetic variability of half-smooth tongue sole *Cynoglossus semilaevis* populations using microsatellite markers. *Acta Oceanologica Sinica*, **30**, 76-83. doi:10.1007/s13131-011-0121-8

Liu SYV, Dai CF, Allen GR, Erdmann MV (2012) Phylogeography of the neon damselfish *Poamcentrus coelestis* indicates a cryptic species and different species origins in the West Pacific Ocean. *Marine Ecology Progress Series*, **458**, 155-167. doi:10.3354/meps09648

Liu SV, Chang F, Borsa P, Chen W & Dai C (2014a) Phylogeography of the humbug damselfish, *Dascyllus aruanus* (Linnaeus, 1758): Evidence of Indo-Pacific vicariance and genetic differentiation of peripheral populations. *Biological Journal of the Linnean Society*, **113**, 931-942. doi:10.1111/bij.12378

Liu B, Gao T & Liu J (2014b) Development of 17 novel polymorphic microsatellites in the small yellow croaker *Larimichthys polyactis*. *Conservation Genetics Resources*, **6**, 397-399. doi:10.1007/s12686-013-0102-7

Liu B, Zhang B, Xue D, Gao T & Liu J (2016) Population structure and adaptive divergence in a high gene flow marine fish: The small yellow croaker (*Larimichthys polyactis*). *PLoS ONE*, **11**, e0154020. doi:10.1371/journal.pone.0154020

Liu SYV, Tuanmu M, Rachmawati R, Mahardika GN & Barber PH (2019a) Integrating phylogeographic and ecological niche approaches to delimitating cryptic lineages in the blue-green damselfish (*Chromis viridis*). *PeerJ*, **7**, e7384. doi:10.7717/peerj.7384

Liu L, Zhang X, Sun D, Gao T & Song N (2019b) Population genetic structure of *Liza affinis* (Eastern keelback mullet), reveals high gene flow inferred from microsatellite analysis. *Ocean Science Journal*, **54**, 245-256. doi:10.1007/s12601-019-0013-y

Liu L, Zhang X, Li C, Zhang H, Yanagimoto T, Song N & Gao T (2019c) Population genetic structure of marbled rockfish, *Sebastiscus marmoratus* (Cuvier, 1829), in the northwestern Pacific Ocean. *Zookeys*, **830**, 127-144. doi:10.3897/zookeys.830.30586

Lotterhos KE & Markel RW (2012) Oceanographic drivers of offspring abundance may increase or decrease reproductive variance in a temperate marine fish. *Molecular Ecology*, **21**, 5009-5026. doi:10.1111/j.1365-294X.2012.12002.x

Lotterhos KE, Dick SJ & Haggarty DR (2014) Evaluation of rockfish conservation areas networks in the United States and Canada relative to the dispersal distance for black rockfish (*Sebastes melanops*). *Evolutionary Applications*, **7**, 238-259. doi:10.1111/eva.12115

López MD, Uribe-Alcocer M & Jaimes PD (2010b) Phylogeography and historical demography of the Pacific Sierra mackerel (*Scomberomorus sierra*) in the Eastern Pacific. *BMC Genetics*, **11**, 34. doi:10.1186/1471-2156-11-34

López A, Vera M, Otero-Ferrer F, Pardo BG, Martínez P, Molina L & Bouza C (2010a) Species identification and genetic structure of threatened seahorses in Gran Canaria Island (Spain) using mitochondrial and microsatellite markers. *Conservation Genetics*, **11**, 2431-2436. doi:10.1007/s10592-010-0116-6

Lü Z, Li H, Liu L, Cui W, Hu X & Wang C (2013) Rapid development of microsatellite markers from the large yellow croaker (*Pseudosciaena crocea*) using next generation DNA sequencing technology. *Biochemical Systematics and Ecology,* **51**, 314-319. doi:10.1016/j.bse.2013.09.019

Lundy CJ, Rico C & Hewitt GM (2000) Temporal and spatial genetic variation in spawning grounds of European hake (*Merluccius merluccius*) in the Bay of Biscay. *Molecular Ecology*, **9**, 2067-2079. doi:10.1046/j.1365-294X.2000.01120.x

Lynch AJ (2008) *A molecular analysis of Atlantic Menhaden (*Brevoortia tyrannus*) stock structure*, College of William and Mary.

Lynch AJ, Mcdowell JR & Graves JE (2010) A molecular genetic investigation of the population structure of Atlantic menhaden (*Brevoortia tyrannus*). *Fishery Bulletin*, **108**, 87-97.

Ma H & Chen S (2009) Isolation and characterization of 31 polymorphic microsatellite markers in barfin flounder (*Verasper moseri*) and the cross-species amplification in spotted halibut (*Verasper variegatus*). *Conservation Genetics*, **10**, 1591-1595. doi:10.1007/s10592-008-9801-0

Ma CY, Ma HY & Ma LB (2011a) Development and testing of 13 polymorphic microsatellite markers in *Larimichthys polyactis* (Sciaenidae) using 5' anchored PCR. *Genetics and Molecular Research*, **10**, 1455-1460. doi:10.4238/vol10-3gmr1464

Ma C,Y Ma HH, Ma LB, Jiang K, Cui HY & Ma QQ (2011b) Isolation and characterization of 16 polymorphic microsatellite markers from *Nibea albiflora*. *Fisheries Science*, **77**, 707-711. doi:10.1007/s12562-011-0376-

Ma KY, van Herwerden L, Newman SJ, Berumen ML, Choat JH, Chu KH & Sadovy de Mitcheson Y (2018) Contrasting population genetic structure in three aggregating groupers (Percoidei: Epinephelidae) in the Indo-West Pacific: The importance of reproductive mode. *BMC Evolutionary Biology*, **18**, 180. doi:10.1186/s12862-018-1284-0

Mach ME, Sbrocco EJ, Hice LA, Duffy TA, Conover DO & Barber PH (2011) Regional differentiation and post-glacial expansion of the Atlantic silverside, *Menidia menidia*, an annual fish with high dispersal potential. *Marine Biology*, **158**, 515-530. doi:10.1007/s00227-010-1577-3

Machado-Schiaffino G, Juanes F, Garcia-Vazquez E (2010) Introgressive hybridization in North American hakes after secondary contact. *Molecular Phylogenetics and Evolution*, **55**, 552-558. doi:10.1016/j.ympev.2010.01.034

Machado-Schiaffino G & Garcia-Vazquez E (2011) Population structure of long tailed hake *Macruronus magellanicus* in the Pacific and Atlantic oceans: Implications for fisheries management. *Fisheries Research*, **111**, 164-169. doi:10.1016/j.fishres.2011.07.007

Machado-Schiaffino G, Juanes F, Garcia-Vazquez E (2011) Identifying unique populations in long-dispersal marine species: Gulfs as priority conservation areas. *Biological Conservation*, **144**, 330-338. doi:10.1016/j.biocon.2010.09.010

Mackiewicz M, Porter BA, Dakin EE & Avise JC (2005) Cuckoldry rates in the Molly Miller (*Scartella cristata*; blenniidae), a hole-nesting marine fish with alternative reproductive tactics. *Marine Biology*, **148**, 213-221. doi:10.1007/s00227-005-0010-9

Madduppa HH, Timm J & Kochzius M (2014) Interspecific, spatial and temporal variability of self-recruitment in anemonefishes. *PLoS ONE*, **9**, e90648. doi:10.1371/journal.pone.0090648

Madsen ML, Nelson RJ, Fevolden S, Christiansen JS & Præbel K (2016) Population genetic analysis of Euro-Arctic polar cod *Boreogadus saida* suggests fjord and oceanic structuring. *Polar Biology*, **39**, 969-980. doi:10.1007/s00300-015-1812-y

Maduna SN, da Silva C, Wintner SP, Roodt-Wilding R & Bester-van der Merwe AE (2016) When two oceans meet: Regional population genetics of an exploited coastal shark, *Mustelus mustelus*. *Marine Ecology Progress Series*, **544**, 183-196. doi:10.3354/meps11596

Maduna SN, Rossouw C, da Silva C, Soekoe M & Bester-van der Merwe AE (2017) Species identification and comparative population genetics of four coastal houndsharks based on novel NGS-mined microsatellites. *Ecology and Evolution*, **7**, 1462-1486. doi:10.1002/ece3.2770

Magallón-Gayón E, Diaz-Jaimes P & Uribe-Alcocer M (2016) Spatial and temporal genetic homogeneity of the Monterey Spanish mackerel, *Scomberomorus concolor*, in the Gulf of California. *PeerJ*, **4**, e2583. doi:10.7717/peerj.2583

Maggio T, Brutto SL, Garoia F, Tinti F & Arculeo M (2009) Microsatellite analysis of red mullet *Mullus barbatus* (Perciformes, Mullidae) reveals the isolation of the Adriatic Basin in the Mediterranean Sea. *ICES Journal of Marine Science*, **66**, 1883-1891. doi:10.1093/icesjms/fsp160

Mamoozadeh NR, McDowell JR, Rooker JR & Graves JE (2017) Genetic evaluation of population structure in white marlin (*Kajikia albida*): The importance of statistical power. *ICES Journal of Marine Science*, **75**, 892-902. doi:10.1093/icesjms/fsx047

Mariani S, Hutchinson WF, Hatfield EMC, Ruzzante DE, Simmonds EJ, Dahlgren TG, André C, Brigham J, Torstensen E & Carvalho GR (2005) North Sea herring population structure revealed by microsatellite analysis. *Marine Ecology Progress Series*, **303**, 245-257. doi:10.3354/meps303245

Marko PB, Rogers-Bennett L & Dennis AB (2007) mtDNA population structure and gene flow in lingcod (*Ophiodon elongatus*): Limited connectivity despite long-lived pelagic larvae. *Marine Biology*, **150**, 1301-1311. doi:10.1007/s00227-006-0395-0

Matala AP, Gray AK, Gharrett AJ & Love MS (2004b) Microsatellite variation indicates population genetic structure of bocaccio. *North American Journal of Fisheries Management*, **24**, 1189-1202. doi:10.1577/M03-061.1

Matala AP, Gray AK, Heifetz J & Gharrett AJ (2004a) Population structure of Alaskan shortraker rockfish, *Sebastes borealis*, inferred from microsatellite variation. In *Genetics of Subpolar Fish and Invertebrates*, 201-210. Springer, Dordrecht.

Matias AMA, Anticamara JA & Quilang JP (2013) High gene flow in reef fishes and its implications for ad-hoc no-take marine reserves. *Mitochondrial DNA*, **24**, 584-595. doi:10.3109/19401736.2013.772147

Matschiner M, Hanel R & Salzburger W (2009) Gene flow by larval dispersal in the Antarctic notothenioid fish *Gobionotothen gibberifrons*. *Molecular Ecology*, **18**, 2574-2587. doi:10.1111/j.1365-294X.2009.04220.x

Matić-Skoko S, Šegvić-Bubić T, Mandić I, Izquierdo-Gomez D, Arneri E, Carbonara P, Grati F, Ikica Z, Kolitari J, Milone N, Sartor P, Scarcella G, Tokaç A & Tzanatos E (2018) Evidence of subtle genetic structure in the sympatric species *Mullus barbatus* and *Mullus surmuletus* (Linnaeus, 1758) in the Mediterranean Sea. *Scientific Reports*, **8**, 676.

McCauley L, Goecker C, Parker P, Rudolph T, Goetz F & Gerlach G (2004) Characterization and isolation of DNA microsatellite primers in the spiny dogfish (*Squalus acanthias*). *Molecular Ecology Notes*, **4**, 494-496. doi:10.1111/j.1471-8286.2004.00724.x

McClelland G, Melendy J, Osborne J, Reid D & Douglas S (2005) Use of parasite and genetic markers in delineating populations of winter flounder from the central and south-west Scotian Shelf and north-east Gulf of Maine. *Journal of Fish Biology*, **66**, 1082-1100. doi:10.1111/j.0022-1112.2005.00659.x

McCraney WT, Goldsmith G, Jacobs DK & Kinziger AP (2010) Rampant drift in artificially fragmented populations of the endangered tidewater goby (*Eucyclogobius newberryi*). *Molecular Ecology*, **19**, 3315-3327. doi:10.1111/j.1365-294X.2010.04755.x

McCusker MR & Bentzen P (2010) Historical influences dominate the population genetic structure of a sedentary marine fish, Atlantic wolffish (*Anarhichas lupus*), across the North Atlantic Ocean. *Molecular Ecology*, **19**, 4228-4241. doi:10.1111/j.1365-294X.2010.04806.x

McDowell JR, Carlsson JEL & Graves JE (2007) Genetic analysis of blue marlin (*Makaira nigricans*) stock structure in the Atlantic Ocean. *Gulf and Caribbean Research*, **19**, 75-82. doi:10.18785/gcr.1902.09

McGowan C & Reith ME (1999) Polymorphic microsatellite markers for Atlantic halibut, *Hippoglossus hippoglossus*. *Molecular Ecology*, **8**, 1761-1763. doi:10.1046/j.1365-294x.1999.00723-5.x

McPherson AA, O'Reilly PT, McParland TL, Jones MW & Bentzen P (2001a) Isolation of nine novel tetranucleotide microsatellites in Atlantic herring (*Clupea harengus*). *Molecular Ecology Notes*, **1**, 31-32. doi:10.1046/j.1471-8287.2000.00012.x

McPherson AA, Stephenson RL, O'Reilly PT, Jones MW & Taggart CT (2001b) Genetic diversity of coastal Northwest Atlantic herring populations: Implications for management. *Journal of Fish Biology*, **59**, 356-370. doi:10.1111/j.1095-8649.2001.tb01396.x

McWilliam RA, Minchinton TE & Ayre DJ (2015) Characterization of 13 polymorphic microsatellite markers for *Trachinops caudimaculatus* (McCoy, 1890) developed using 454-sequencing. *Conservation Genetics Resources*, **7**, 539-541. doi:10.1007/s12686-014-0418-y

Menezes MR, Noguchi D, Nakajima M & Taniguchi N (2008) Microsatellite development and survey of genetic variation in skipjack tuna *Katsuwonus pelamis*. *Journal of Fish Biology*, **73**, 463-473. doi:10.1111/j.1095-8649.2008.01912.x

Menezes MR, Kumar G & Kunal SP (2012) Population genetic structure of skipjack tuna *Katsuwonus pelamis* from the Indian coast using sequence analysis of the mitochondrial DNA D-loop region. *Journal of Fish Biology*, **80**, 2198-2212. doi:10.1111/j.1095-8649.2012.03270.x

Miao G-D, Shao C-W, Liao X-L, Ma H-Y, Tian Y-S & Chen S-L (2009) Development of polymorphic microsatellite markers from barfin flounder (*Verasper moseri*) and their cross-species amplification. *Conservation Genetics*, **10**, 701-703. doi:10.1007/s10592-008-9619-9

Milana V, Ciampoli M & Sola L (2014) mtDNA sequences of *Sphyraena viridensis* (Perciformes: Sphyraenidae) from Italy: Insights into historical events and the phylogeny of the genus. *Biological Journal of the Linnean Society*, **113**, 635-641. doi:10.1111/bij.12353

Miller JA, Banks MA, Gomez-Uchida D & Shanks AL (2005) A comparison of population structure in black rockfish (*Sebastes melanops*) as determined with otolith microchemistry and microsatellite DNA. *Canadian Journal of Fisheries and Aquatic Sciences*, **62**, 2189-2198. doi:10.1139/f05-133

Miller-Sims V, Atema J, Kingsford MJ & Gerlach G (2004) Characterization and isolation of DNA microsatellite primers in the cardinalfish (*Apogon doederleini*). *Molecular Ecology Notes*, **4**, 336-338. doi:10.1111/j.1471-8286.2004.00689.x

Miller-Sims V, Delaney M, Atema J, Kingsford M & Gerlach G (2005a) DNA microsatellites in *Acnthochromis polyacanthus*. *Molecular Ecology Notes*, **5**, 841-843. doi:10.1111/j.1471-8286.2005.01081.x

Miller-Sims V, Delaney M, Atema J, Kingsford MJ & Gerlach G (2005b) DNA microsatellites in the neon damselfish (*Pomacentrus coelestis*). *Molecular Ecology Notes*, **5**, 424-426. doi:10.1111/j.1471-8286.2005.00950.x

Miller-Sims VC, Gerlach G, Kingsford MJ & Atema J (2008) Dispersal in the spiny damselfish, *Acanthochromis polyacanthus*, a coral reef fish species without a larval pelagic stage. *Molecular Ecology*, **17**, 5036-5048. doi:10.1111/j.1365-294X.2008.03986.x

Miralles L, Juanes F & Garcia-Vazquez E (2014) Interoceanic sex-biased migration in bluefish. *Transactions of the American Fisheries Society*, **143**, 1308-1315. doi:10.1080/00028487.2014.935480

Misawa R, Narimatsu Y, Endo H & Kai Y (2019) Population structure of the ocellate spot skate (*Okamejei kenojei*) inferred from variations in mitochondrial DNA (mtDNA) sequences and from morphological characters of regional populations. *Fishery Bulletin*, **117**, 24+.

Mitchell DM (2006) *Biocomplexity and metapopulation dynamics of Pacific herring (*Clupea pallasii*) in Puget Sound, Washington*, University of Washington.

Mobley KB, Small CM, Jue NK & Jones AG (2010) Population structure of the dusky pipefish (*Syngnathus floridae*) from the Atlantic and Gulf of Mexico, as revealed by mitochondrial DNA and microsatellite analyses. *Journal of Biogeography*, **37**, 1363-1377. doi:10.1111/j.1365-2699.2010.02288.x

Montanari SR, van Herwerden L, Pratchett MS, Hobbs JA & Fugedi A (2012) Reef fish hybridization: Lessons learnt from butterflyfishes (genus *Chaetodon*). *Ecology and Evolution*, **2**, 310-328. doi:10.1002/ece3.83

Montes I, Iriondo M, Manzano C, Arrizabalaga H, Jiménez E, Pardo MÁ, Goñi N, Davies CA & Estonba A (2012) Worldwide genetic structure of albacore *Thunnus alalunga* revealed by microsatellite DNA markers. *Marine Ecology Progress Series*, **471**, 183-191. doi:10.3354/meps09991

Morishima K, Yamamoto H, Sawada Y, Miyashita S & Kato K (2009) Developing 23 new polymorphic microsatellite markers and simulating parentage assignment in the Pacific bluefin tuna, *Thunnus orientalis*. *Molecular Ecology Resources*, **9**, 790-792. doi:10.1111/j.1755-0998.2008.02144.x

Mukai T, Nakamura S & Nishida M (2009) Genetic population structure of a reef goby, *Bathygobius cocosensis*, in the northwestern Pacific. *Ichthyological Research*, **56**, 380. doi:10.1007/s10228-009-0111-4

Munguía-Vega A, Marinone SG, Paz-García DA, Giron-Nava A, Plomozo-Lugo T, Gonzalez-Cuellar O, Weaver AH, García-Rodriguez FJ, Reyes-Bonilla H (2018) Anisotropic larval connectivity and metapopulation structure driven by directional oceanic currents in a marine fish targeted by small-scale fisheries. *Marine Biology*, **165**, 16. doi:10.1007/s00227-017-3267-x

Muths D, Grewe P, Jean C & Bourjea J (2009) Genetic population structure of the swordfish (*Xiphias gladius*) in the southwest Indian Ocean: Sex-biased differentiation, congruency between markers and its incidence in a way of stock assessment. *Fisheries Research*, **97**, 263-269. doi:10.1016/j.fishres.2009.03.004

Muths D & Bourjea J (2011) Characterization of thirteen new polymorphic microsatellite markers from the honeycomb grouper *Epinephelus merra*. *Conservation Genetics Resources*, **3**, 629-631. doi:10.1007/s12686-011-9420-9

Muths D, Tessier E, Guows G, Craig M, Mwale M, Mwaluma J, Mwandya A & Bourjea J (2011) Restricted dispersal of the reef fish *Myripristis berndti* at the scale of the SW Indian Ocean. *Marine Ecology Progress Series*, **443**, 167-180. doi:10.3354/meps09394

Muths D, Rastorgueff P, Selva M & Chevaldonné P (2015) Local scale connectivity in the cave-dwelling brooding fish *Apogon imberbis*. *Journal of Sea Research*, **95**, 70-74. doi:10.1016/j.seares.2014.10.009

Mzingirwa FA, Mkare TK, Nyingi DW & Njiru (2019) Genetic diversity and spatial population structure of a deepwater snapper, *Pristipomoides filamentosus* in the south-west Indian Ocean. *Molecular Biology Reports*, **46**, 5079-5088. doi:10.1007/s11033-019-04962-w

Naciri M, Lemaire C, Borsa P & Bonhomme F (1999) Genetic study of the Atlantic/Mediterranean transition in sea bass (*Dicentrarchus labrax*). *Journal of Heredity*, **90**, 591-596. doi:10.1093/jhered/90.6.591

Nakajima K, Kitada S, Habara Y, Sano S, Yokoyama E, Sugaya T, Iwamoto A, Kishino H & Hamasaki K (2014) Genetic effects of marine stock enhancement: A case study based on the highly piscivorous Japanese Spanish mackerel. *Canadian Journal of Fisheries and Aquatic Sciences*, **71**, 301-314. doi:10.1139/cjfas-2013-0418

Nance HA, Klimley P, Galvá-Magaña F, Martínez-Ortíz J & Marko PB (2011) Demographic processes underlying subtle patterns of population structure in the scalloped hammerhead shark, *Sphyrna lewini*. *PLoS ONE*, **6**, e21459. doi:10.1371/journal.pone.0021459

Neethling M, Matthee CA, Bowie RCK & von der Heyden S (2008) Evidence for panmixia despite barriers to gene flow in the southern African endemic, *Caffrogobius caffer* (Teleosti: Gobiidae). *BMC Evolutionary Biology*, **8**, 325. doi:10.1186/1471-2148-8-325

Nielsen EE, Hansen MM, Rozzante DE, Meldrup D & Grønkjær P (2003) Evidence of a hybrid-zone in Atlantic cod (*Gadus morhua*) in the Baltic and the Danish Belt Sea revealed by individual admixture analysis. *Molecular Ecology*, **12**, 1497-1508. doi:10.1046/j.1365-294X.2003.01819.x

Nielsen EE, Neilsen PH, Meldrup D & Hansen MM (2004) Genetic population structure of turbot (*Scophthalmus maximus* L.) supports the presence of multiple hybrid zones for marine fishes in the transition zone between the Baltic Sea and the North Sea. *Molecular Ecology*, **13**, 585-595. doi:10.1046/j.1365-294X.2004.02097.x

Nielsen JL, Graziano SL & Seitz AC (2010) Fine-scale population genetic structure in Alaskan Pacific halibut (*Hippoglossus stenolepis*). *Conservation Genetics*, **11**, 999-1012. doi:10.1007/s10592-009-9943-8

Nirichio M, Gaviria JI, Siccha-Ramirez ZR, Oliveira C, Foresti F, Milana V & Rossi AR (2019) Chromosomal polymorphism and molecular variability in the pearly razorfish *Xyrichtys novacula* (Labriformes, Labridae): taxonomic and biogeographic implications. *Genetica*, **147**, 47-56. doi:10.1007/s10709-019-00051-9

Noguchi S, Itoi S, Takai N, Noda T, Myojin T, Yoshihara K & Sugita H (2012) Population genetic structure of *Scombrops boops* (Percoid, Scombropidae) around the Japanese archipelago inferred from the cytochrome *b* gene sequence in mitochondrial DNA. *Mitochondrial DNA*, **23**, 223-229. doi:10.3109/19401736.2012.668897

Nohara K, Takeuchi H, Tsuzaki T, Suzuki N, Tominaga O & Seikai T (2009a) Genetic variability and stock structure of red tilefish *Branchiostegus japonicus* inferred from mtDNA sequence analysis. *Fisheries Science,* **76**, 75. doi:10.1007/s12562-009-0188-8

Nohara K, Kokita T, Tomnaga O & Seikai T (2009b) Isolation and characterization of 11 polymorphic microsatellite loci in the white girdled goby (*Pterogobius zonoleucus*) and cross-species amplification in the serpentine goby (*P. elapoides*). *Molecular Ecology Resources*, **9**, 610-612. doi:10.1111/j.1471-8286.2009.00228_9_2.x

Nuñez JJ, González MT & Pérez-Losado M (2010) Testing species boundaries between Atlantic and Pacific lineages of the Patagonian rockfish *Sebastes oculatus* (Teleostei: Scorpaenidae) through mitochondrial DNA sequences. *Revista de Biología Marina y Oceanografía*, **45**, 565-573.

O'Connell M, Dillon MC, Wright JM (1998a) Development of primers for polymorphic microsatellite loci in the Pacific herring (*Clupea harengus pallasi*). *Molecular Ecology,* **7**, 358-360.

O'Connell M, Dillon MC, Wright JM, Bentzen P, Merkouris S & Seeb J (1998b) Genetic structuring among Alaskan Pacific herring populations identified using microsatellite variation. *Journal of Fish Biology*, **53**, 150-163. doi:10.1111/j.1095-8649.1998.tb00117.x

O'Donnell JL, Beldade R, Mills SC, Williams HE & Bernardi G (2017) Life history, larval dispersal, and connectivity in coral reef fish among the scattered Islands of the Mozambique Channel. *Coral Reefs*, **36**, 223-232. doi:10.1007/s00338-016-1495-z

O'Donnell TP, Reichert MJM & Darden TL (2019) Genetic population structure of white grunt in the southeastern United States. *North American Journal of Fisheries Management*, **39**, 725-737. doi:10.1002/nafm.10306

O'Leary DB, Coughlan J, Dillane E, McCarthy TV & Cross TF (2007) Microsatellite variation in cod *Gadus morhua* throughout its geographic range. *Journal of Fish Biology*, **70**, 310-335. doi:10.1111/j.1095-8649.2007.01451.x

O'Reilly PT, McPherson AA, Kenchington E, Taggart C, Jones MW & Bentzen P (2002) Isolation and characterization of tetranucleotide microsatellites from Atlantic haddock (*Melanogrammus aeglefinus*). *Marine Biotechnology*, **4**, 418-422. doi:10.1007/s10126-002-0010-4

O'Reilly PT, Canino MF, Bailey KM & Bentzen P (2004) Inverse relationship between FST and microsatellite polymorphism in the marine fish, walleye pollock (*Theragra chalcogramma*): Implications for resolving weak population structure. *Molecular Ecology*, **13**, 1799-1814. doi:10.1111/j.1365-294X.2004.02214.x

Olsen JB, Lewis CJ, Kretschmer EJ, Wilson SL & Seeb JE (2002) Characterization of 14 tetranucleotide microsatellite loci derived from Pacific herring. *Molecular Ecology*, **2**, 101-103.

Ortega-Villaizán Romo M, Nakajima M & Taniguchi N (2003) Isolation and characterization of microsatellite DNA markers in the rare species barfin flounder (*Verasper moseri*) and its closely related species spotted halibut (*V. variegatus*). *Molecular Ecology Notes*, **3**, 629-631. doi:10.1046/j.1471-8286.2003.00536.x

Ortega-Villaizán Romo MDM, Aritaki M, Suzuki S, Ikeda M, Asahida T & Taniguchi N (2006a) Genetic population evaluation of two closely related flatfish species, the rare barfin flounder and spotted halibut, along the Japanese coast. *Fisheries Science*, **72**, 556-567. doi:10.111/j.1444-2906.2006.01184.x

Ortega-Villaizán Romo MDM, Aritaki M & Taniguchi N (2006b) Pedigree analysis of recaptured fish in the stock enhancement program of spotted halibut *Verasper variegatus*. *Fisheries Science*, **72**, 48-52. doi:10.1111/j.1444-2906.2006.01115.x

Otwoma LM, Reuter H, Timm J & Meyer A (2018a) Genetic connectivity in a herbivorous coral reef fish (*Acanthurus leucosternon* Bennet, 1833) in the Eastern African region. *Hydrobiologia*, **806**, 237-250. doi:10.1007/s10750-017-3363-4

Otwoma LM, Diemel V, Reuter H, Kochzius M & Meyer A (2018b) Genetic population structure of the convict surgeonfish *Acanthurus triostegus*: A phylogeographic reassessment across its range. *Journal of Fish Biology*, **93**, 597-608. doi:10.1111/jfb.13686

Otwoma LM, Diemel V, Reuter H, Kochzius M & Meyer A (2018b) Genetic population structure of the convict surgeonfish *Acanthurus triostegus*: A phylogeographic reassessment across its range. *Journal of Fish Biology*, **93**, 597-608. doi:10.1111/jfb.13686

Otwoma LM & Reuter H (2019) Do differences in mating behaviour lead to differences in connectivity patterns of reef fishes? Insights from two sympatric surgeonfish species in the Indian Ocean. *Marine Environmental Research*, **151**, 104760. doi:10.1016/j.marenvres.2019.104760

Ouazzani KC, Benazzou T, Charouki N, Bonhomme F & Chlaida M (2017) Genetic differentiation of European anchovy (*Engraulis encrasicolus* along the Moroccan coast reveals a phylogeographic break around the 25th parallel North. *Marine Biology Research*, **13**, 342-350. doi:10.1080/17451000.2016.1257811

Oury N, Duchatelet L, Mallefet J & Magalon H (2019) Isolation and characterization of 29 and 19 microsatellite loci from two deep-sea luminous lanternsharks, *Etmopterus spinax* and *Etmopterus molleri* (Squaliformes, Etmopteridae). *Molecular Biology Reports*, **46**, 1357-1362. doi:10.1007/s11033-018-04578-6

Ovenden JR, Morgan JAT, Street R, Tobin A, Simpfendorfer C, Macbeth W & Welch D (2011) Negligible evidence for regional genetic population structure for two shark species *Rhizoprionodon acutus* (Rüppell, 1837) and *Sphyrna lewini* (Griffith & Smith, 1834) with contrasting biology. *Marine Biology*, **158**, 1497-1509. doi:10.1007/s00227-011-1666-y

Pakaki V, Magoulas A & Kasapidis P (2009) New polymorphic microsatellite loci for population studies in the European anchovy, *Engraulis encrasicolus* (L.). *Molecular Ecology Resources*, **9**, 1406-1409. doi:10.1111/j.1755-0998.2009.02681.x

Palof KJ, Heifetz J & Gharrett AJ (2011) Geographic structure in Alaskan Pacific ocean perch (*Sebastes alutus*) indicates limited lifetime dispersal. *Marine Biology*, **158**, 779-792. doi:10.1007/s00227-010-1606-2

Pálsson S, Källman T, Paulsen J & Árnason E (2010) An assessment of mitochondrial variation in Arctic gadoids. *Polar Biology*, **32**, 471-479. doi:10.1007/s00300-008-0542-9

Pampoulie C, Ruzzante DE, Chosson V, Jörundsdóttir TD, Taylor L, Thorsteinsson V, Daníelsdóttir AK & Marteinsdóttir G (2006) The genetic structure of Atlantic cod (*Gadus morhua*) around Iceland: Insights from microsatellites, the *Pan* I locus, and tagging experiments. *Canadian Journal of Fisheries and Aquatic Sciences*, **63**, 2660-2675. doi:10.1139/f06-150

Pampoulie C & Daníelsdóttir AK (2008) Resolving species identification problems in the genus *Sebastes* using nuclear genetic markers. *Fisheries Research*, **93**, 54-63. doi:10.1016/j.fishres.2008.02.007

Pampoulie C, Gíslason D & Daníelsdóttir AK (2009) A "seascape genetic" snapshot of *Sebastes marinus* calls for further investigation across the North Atlantic. *ICES Journal of Marine Science*, **66**, 2219-2222. doi:10.1093/icesjms/fsp199

Pampoulie C, Skirnisdóttir S, Daníelsdóttir AK & Gunnarsson Á (2012) Genetic structure of the Atlantic wolffish (*Anarhichas lupus* L.) at Icelandic fishing grounds: Another evidence of panmixia in Iceland? *ICES Journal of Marine Science*, **69**, 508-515. doi:10.1093/icesjms/fss017

Pampoulie C, Skirnisdottir S, Olasfdottir G, Helyar SJ, Thorsteinsson V, Jónsson SP, Fréchet A, Durif CMF, Sherman S, Lampart-Kałużniacka M, Hedeholm R, Ólafsson H, Daníelsdóttir AK & Kasper JM (2014) Genetic structure of the lumpfish *Cyclopterus lumpus* across the North Atlantic. *ICES Journal of Marine Science*, **71**, 2390-2397. doi:10.1093/icesjms/fsu071

Pampoulie C, Slotte A, Óskarsson GJ, Helyar SJ, Jónsson Á, Ólasfdóttir G, Skírnisdóttir S, Libungan LA, Jacobsen JA, Joensen H, Nielsen HH, Sigurõsson SK & Daníelsdóttir AK (2015) Stock structure of Atlantic herring *Clupea harengus* in the Norwegian Sea and adjacent waters. *Marine Ecology Progress Series*, **522**, 219-230. doi:10.3354/meps11114

Panithanarak T, Karuwancharoen R, Na-Nakorn U & Nguyen TTT (2010) Population genetics of the spotted seahorse (*Hippocampus kuda*) in Thai waters: Implications for conservation. *Zoological Studies*, **49**, 564-576.

Papetti C, Susana E, La Mesa M, Kock K, Patarnello T & Zane L (2007) Microsatellite analysis reveals genetic differentiation between year classes in the icefish *Chaenocephalus aceratus* at South Shetlands and Elephant Island. *Polar Biology*, **30**, 1605. doi:10.1007/s00300-007-0325

Papetti C, Marino IAM, Agostini C, Bisol PM, Patarnello T &Zane L (2011) Characterization of novel microsatellite markers in the Antarctic silverfish *Pleuragramma antarcticum* and cross species amplification in other Notothenioidei. *Conservation Genetics Resources*, **3**, 259-262. doi:10.1007/s12686-010-9336-9

Papetti C, Pujolar JM, Mezzavilla M, La Mesa M, Rock J, Zane L & Patarnello T (2012) Population genetic structure and gene flow patterns between populations of the Antarctic icefish *Chionodraco rastrospinosus*. *Journal of Biogeography*, **39**, 1361-1372. doi:10.1111/j.1365-2699.2011.02682.x

Papetti C, Di Franco A, Zane L, Guidetti P, De Simone V, Spizzotin M, Zorica B, Keč VČ & Mazzoldi C (2013) Single population and common natal origin for Adriatic *Scomber scombrus* stocks: Evidence from an integrated approach. *ICES Journal of Marine Science*, **70**, 387-398. doi:10.1093/icesjms/fss201

Pardini AT, Jones CS, Scholl MC & Noble LR (2000) Isolation and characterization of dinucleotide microsatellite loci in the great white shark, *Carcharodon carcharias*. *Molecular Ecology*, **9**, 1176-1178. doi:10.1046/j.1365-294x.2000.00954-4.x

Pardo GB, Casas L, Fortes GG, Bouza C, Martínez P, Clark MS & Sánchez L (2005) New microsatellite markers in turbot (*Scophthalmus maximus*) derived from an enriched genomic library and sequence databases. *Molecular Ecology Notes*, **5**, 62-64. doi:10.1111/j.1471-8286.2004.00834.x

Pardo BG, Lopez A, Martínez P & Bouza C (2007) Novel microsatellite loci in the threatened European long-snouted seahorse (*Hippocampus guttulatus*) for genetic diversity and parentage analysis. *Conservation Genetics*, **8**, 1243-1245. doi:10.1007/s10592-006-9241-7

Paterson CN, Chabot CL, Robertson JM, Cota-Nieto JJ, Erisman B & Allen LG (2015) The genetic diversity and population structure of barred sand bass, *Paralabrax nebulifer*: A historically important fisheries species off southern and Baja California. *California Cooperative Oceanic Fisheries Investigations Reports*, **56**, 97-109

Paz-García DA, Munguía-Vega A, Plomozo-Lugo T & Weaver AH (2017) Characterization of 32 microsatellite loci for the Pacific red snapper, *Lutjanus peru*, through next generation sequencing. *Molecular Biology Reports*, **44**, 251-256. doi:10.1007/s11033-017-4105-4

Pazmiño DA, Maes GE, Simpfendorfer CA, Salinas-de-León P & van Herwerden L (2017) Genome-wide SNPs reveal low effective population size within confined management units of the highly vagile Galapagos shark (*Carcharhinus galapagensis*). *Conservation Genetics*, **18**, 1151-1163. doi:10.1007/s10592-017-0967-1

Pedrosa-Gerasmio IR, Agmata AB & Santos MD (2015) Genetic diversity, population genetic structure, and demographic history of *Auxis thazard* (Perciformes), *Selar crumenophthalmus* (Perciformes), *Rastrelliger kanagurta* (Perciformes) and *Sardinella lemuru* (Clupeiformes) in Sulu-Celebes Sea inferred by mitochondrial DNA sequences. *Fisheries Research*, **162**, 64-74. doi:10.1016/j.fishres.2014.10.006

Peng S, Shi Z, Hou J, Wang W, Zhao F & Zhang H (2009) Genetic diversity of silver pomfret (*Pampus argenteus*) populations from the China Sea based on mitochondrial DNA control region sequences. *Biochemical Systematics and Ecology*, **37**, 626-632. doi:10.1016/j.bse.2009.09.003

Pereira AN, Márquez A, Marin M & Marin Y (2009) Genetic evidence of two stocks of the whitemouth croaker *Micropogonias furnieri* in the Río de la Plata and oceanic front in Uruguay. *Journal of Fish Biology*, **75**, 321-331. doi:10.1111/j.1095-8649.2009.02321.x

Perez-Enriquez R & Taniguchi N (1999) Genetic structure of red sea bream (*Pagrus major*) population off Japan and the Southwest Pacific, using microsatellite DNA markers. *Fisheries Science*, **65**, 23-30. doi:10.23331/fishsci.65.23

Phinchongsakuldit J, Chaipakdee P, Collins jF, Jaroensutasinee M & Brookfield JFY (2013) Population genetics of cobia (*Rachycentron canadum*) in the Gulf of Thailand and Andaman Sea: Fisheries management implications. *Aquaculture International*, **21**, 197-217. doi:10.1007/s10499-012-9545-1

Piñeros VJ, Gutiérrez-Rodríguez C & Lance SL (2015b) Development and characterization of 29 microsatellite markers for the sergeant major damselfish (*Abudefduf saxatilis*) using paired-end Illumina shotgun sequencing. *Conservation Genetics Resources*, **7**, 103-105. doi:10.1007/s12686-014-0303-8

Piñeros VJ, Rios-Cardenas O, Gutiérrez-Rodríguez C & Mendoza-Cuenca L (2015a) Morphological differentiation in the damselfish *Abudefduf saxatilis* along the Mexican Atlantic coast is associated with environmental factors and high connectivity. *Evolutionary Biology*, **42**, 235-249. doi:10.1007/s11692-015-9314-y

Piñeros VJ & Gutiérrez-Rodríguez C (2017) Population genetic structure and connectivity in the widespread coral-reef fish *Abudefduf saxatilis*: The role of historic and contemporary factors. *Coral Reefs*, **36**, 877-890. doi:10.1007/s00338-017-1579-4

Pini J, Planes S, Rochel E, Lecchini D & Fauvelot C (2011) Genetic diversity loss associated to high mortality and environmental stress during the recruitment stage of a coral reef fish. *Coral Reefs*, **30**, 399-404. doi:10.1007/s00338-011-0718-6

Pinsky ML, Montes Jr. HR & Palumbi SR (2010) Using isolation by distance and effective density to estimate dispersal scales in anemonefish. *Evolution*, **64**, 2688-2700. doi:10.1111/j.1558-5646.2010.01003.

Pirog A, Ravigné V, Fontaine MC, Rieux A, Gilabert A, Cliff G, Clua E, Daly R, Heithaus MR, Kiszka JJ, Matich P, Nevill JEG, Smoothey AF, Temple AJ, Berggren P, Jaquemet S & Magalon H (2019) Population structure, connectivity, and demographic history of an apex marine predator, the bull shark *Carcharhinus leucas*. *Ecology and Evolution*, **9**, 12980-13000. doi:10.1002/ece3.5597

Pita A, Pérez M, Cerviño S & Presa P (2011) What can gene flow and recruitment dynamics tell us about connectivity between European hake stocks in the Eastern North Atlantic? *Continental Shelf Research*, **31**, 376-387. doi:10.10.1016/j.csr.2010.09.010

Pita A, Leal A, Santafé-Muñoz S, Piñeiro C & Presa P (2016) Genetic inference of demographic connectivity in the Atlantic European hake metapopulation (*Merluccius merluccius*) over a spatio-temporal framework. *Fisheries Research*, **179**, 291-301. doi:10.1016/j.fishres.2016.03.017

Plank SM, Lowe CG, Feldheim KA, Wilson Jr. RR & Brusslan JA (2010) Population genetic structure of the round stingray *Urobatis helleri* (Elasmobranchii: Rajiformes) in southern California and the Gulf of California. *Journal of Fish Biology*, **77**, 329-340. doi:10.1111/j.1095-8649.2010.02677

Ponce M, Infante C, Crespo A, Zuasti E, Pérez L, Funes V, Catanese G, Cárdenas S & Manchado M (2006) Characterization of microsatellite loci for the redbanded seabream, *Pagrus auriga* (Teleostei, Sparidae). *Molecular Ecology Notes*, **6**, 527-529. doi:10.1111/j.1471-8286.2006.01307.x

Poortvliet M, Longo GC, Selkoe K, Barber PH, White C, Caselle JE, Perez-Matus A, Gaines SD & Bernardi G (2013) Phylogeography of the California sheephead, *Semicossyphus pulcher*: The role of deep reefs as stepping stones and pathways to antitropicality. *Ecology and Evolution*, **3**, 4558-4571. doi:10.1002/ece3.840

Porta D, Porta JM, Porta J, Andree K & Duncan N (2010) Isolation and characterization of microsatellite loci from *Argyrosomus regius* (Asso, 1801), unpublished (http://www.ncbi.nlm.nih.gov)

Portnoy DS, Hollenbeck CM, Bethea DM, Frazier BS, Gelsleichter J & Gold JR (2016) Population structure, gene flow and historical demography of a small coastal shark (*Carcharhinus isodon*) in US waters of the Western Atlantic Ocean. *ICES Journal of Marine Science*, **73**, 2322-2332. doi:10.1093/icesjms/fsw098

Poulsen NA, Nielsen EE, Schierup MH, Loeschcke V & Grønkjær P (2006) Long-term stability and effective population size in North Sea and Baltic Sea cod (*Gadus morhua*). *Molecular Ecology*, **15**, 321-331. doi:10.1111/j.1365-294X.2005.02777.x

Priest MA, Halford AR & McIlwain JL (2012) Evidence of stable genetic structure across a remote island archipelago through self-recruitment in a widely dispersed coral reef fish. *Ecology and Evolution*, **2** 3195-3213. doi:10.1002/ece3.260

Puebla O, Bermingham E & Guichard F (2008) Population genetic analyses of *Hypoplectrus* coral reef fishes provide evidence that local processes are operating during the early stages of marine adaptive radiations. *Molecular Ecology*, **17**, 1405-1415. doi:10.1111/j.1365-294X.2007.03654.x

Puebla O, Bermingham E & Guichard F (2009) Estimating dispersal from genetic isolation by distance in a coral reef fish (*Hypoplectrus puella*). *Ecology*, **90**, 3087-3098. doi:10.1890/08-0859.1

Pujolar JM, Schiavina M, Di Franco A, Melià P, Guidetti P, Gatto M, De Leo GA & Zane L (2013) Understanding the effectiveness of marine protected areas using genetic connectivity patterns and Lagrangian simulations. *Diversity and Distributions*, **19**, 1531-1542. doi:10.1111/ddi.12114

Pumitinsee P, Senanan W, Na-Nakorn U, Kamonrat W & Koedprang W (2009) Temporal genetic heterogeneity of juvenile orange-spotted grouper (*Epinephelus coioides*, Pisces: Serranidae). *Aquaculture Research*, **40**, 1111-1122. doi:10.1111/j.1365-2109.2009.02206.x

Purcell JFH, Cowen RK, Hughes CR & Williams DA (2006) Weak genetic structure indicates strong dispersal limits: A tale of two coral reef fish. *Proceedings of the Royal Society B: Biological Sciences*, **273**, 1483-1490. doi:10.1098/rspb.2006.3470

Purcell JFH, Cowen RK, Hughes CR & Williams DA (2009) Population structure in a common Caribbean coral-reef fish: Implications for larval dispersal and early life-history traits. *Journal of Fish Biology*, **74**, 403-417. doi:10.1111/j.1095-8649.2008.02078.x

Pusack TJ, Christie MR, Johnson DW, Stallings CD & Hixon MA (2014) Spatial and temporal patterns of larval dispersal in a coral-reef fish metapopulation: Evidence of variable reproductive success. *Molecular Ecology*, **23**, 3396-3408. doi:10.1111/mec.12824

Qiu F & Miyamoto MM (2011) Use of nuclear DNA data to estimate genetic diversity and population size in Pacific bluefin and yellowfin tuna (*Thunnus orientalis* and *T. albacares*). *Copeia*, **2**, 264-269. doi:10.1643/CI-10-112

Rabone M, Lavery SD, Little A & Clements KD (2015) Discordance between nuclear and mitochondrial DNA analyses of population structure in closely related triplefin fishes (*Forsterygion lapillum* and *F. capito*, F. Tripterygiidae) supports speciation with gene flow. *Marine Biology*, **162**, 1611-1624. doi:10.1007/s00227-015-2697-6

Radhakrishnan DP, Nedumpally V, Kathirvelpandian A, Saidmuhammed BV & Gopalakrishnan A (2018) Population structure of Spanish mackerel *Scomberomorus commerson* (Lacepede 1800) in the Northern Indian Ocean determined using microsatellite markers. *Aquatic Living Resources*, **31**, 22. doi:10.1051/alr/2018011

Ramírez MA, Patricia-Acevedo P, Planas S, Carlin JL, Funk SM & McMillan WO (2006) New microsatellite resources for groupers (Serranidae). *Molecular Ecology Notes*, **6**, 813-817. doi:10.1111/j.1471-8286.2006.01354.x

Ravago-Gotanco RG & Juinio-Meñez MA (2010) Phylogeography of the mottled spinefoot *Siganus fuscescens*: Pleistocene divergence and limited genetic connectivity across the Philippine archipelago. *Molecular Ecology*, **19**, 4520-4534. doi:10.1111/j.1365-294X.2010.04803.x

Ravago-Gotanco R, Lumibao CY & Pante MJR (2010) Isolation and characterization of thirteen microsatellite markers for the rabbitfish, *Siganus fuscescens*. *Conservation Genetics Resources*, **2** 225-227. doi:10.1007/s12686-009-9148-y

Ravago-Gotanco R, de la Cruz TL, Pante MJ & Borsa P (2018) Cryptic genetic diversity in the mottled rabbitfish *Siganus fuscescens* with mitochondrial introgression at a contact zone in the South China Sea. *PLoS ONE*, **13**, e0193220. doi:10.1371/journal.pone.019322

Reid K, Hoareau TB, Graves JE, Potts WM, dos Santos SMR, Klopper AW & Bloomer P (2016) Secondary contact and asymmetrical gene flow in a cosmopolitan marine fish across the Benguela upwelling zone. *Heredity*, **117**, 307-315.

Reilly A & Ward RD (1999) Microsatellite loci to determine population structure of the Patagonian toothfish *Dissostichus eleginoides*. *Molecular Ecology*, **8**, 1753-1768.

Ren G, Liu Q, Gao T & Yanagimoto T (2013) Population demography and genetic structure of the fat greenling (*Hexagrammos otakii*) inferred from mtDNA control region sequence analyses. *Biochemical Systematics and Ecology*, **47**, 156-163. doi:10.1016/j.bse.2012.09.026

Ren GJ, Hu JJ, Gao TX & Han ZQ (2015) Population structure and genetic diversity of *Ammodytes personatus* in the Northwestern Pacific revealed by microsatellites markers. *Biochemical Systematics and Ecology*, **61**, 303-311. doi:10.1016/j.bse.2015.06.031

Renshaw MA, Patton JC, Rexroad III CE & Gold JR (2006) PCR primers for trinucleotide and tetranucleotide microsatellites in greater amberjack *Seriola dumerili*. *Molecular Ecology Notes*, **6**, 1162-1164. doi:10.1111/j.1471-8286.2006.01474.x

Renshaw MA, Karlsson S & Gold JR (2007) Isolation and characterization of microsatellites in lane snapper (*Lutjanus synagris*), mutton snapper, (*Lutjanus analis*), and yellowtail snapper (*Ocyurus chrysurus*). *Molecular Ecology Notes*, **7**, 1084-1087. doi:10.1111/j.1471-8286.2007.01785.x

Renshaw MA, Douglas KC, Rexroad III CE, Jobity AMC & Gold JR (2009b) Isolation and characterization of microsatellite markers in the Serra Spanish mackerel, *Scomberomorus brasiliensis*. *Molecular Ecology Resources*, **9**, 835-838. doi:10.1111/j.1755-0998.2008.02285.x

Renshaw MA, Gawriluk TR & Gold JR (2009a) Characterization of red drum microsatellite markers in spotted seatrout. *North American Journal of Aquaculture*, **71**, 374-379. doi:10.1577/A08-070.1

Renshaw MA, Portnoy DS & Gold JR (2010) PCR primers for nuclear-encoded microsatellites of the groupers *Cephalopholis fulva* (coney) and *Epinephelus guttatus* (red hind). *Conservation Genetics*, **11**, 1197-1202. doi:10.1007/s10592-009-9918-9

Reza MS, Kinoshita S, Furukawa S, Mochizuki T & Watabe S (2011) Microsatellite and mitochondrial DNA analyses reveal no genetic difference between two pufferfish species torafugu *Takifugu rubripes* and karasu *T. chinensis*. *Fisheries Science*, **77**, 59-67. doi:10.1007/s12562-010-0310-y

Rhodes L, Lewis I & Chapman W (2003) Genetic structure of camouflage grouper, *Epinephelus polyphekadion* (Pisces: Serranidae), in the western central Pacific. *Marine Biology*, **142**, 771-776. doi:10.1007/s00227-002-1002-7

Ribout C, Bech N, Briand MJ, Guyonnet D, Letourneur Y, Brischoux F & Bonnet X (2018) A lack of spatial genetic structure of *Gymnothorax chilospilus* (moray eel) suggests peculiar population functioning. *Biological Journal of the Linnean Society*, **125**, 142-151. doi:10.1093/biolinnean/bly107

Riccioni G, Landi M, Ferrara G, Milano I, Cariani A, Zane L, Sella M, Barbujani G & Tinti F (2010) Spatio-temporal population structuring and genetic diversity retention in depleted Atlantic bluefin tuna of the Mediterranean Sea. *Proceedings of the National Academy of Sciences*, **107**, 2102-2107. doi:10.1073/pnas.0908281107

Ring A, Knutsen H, Fiani D, Hoelzel AR & André C (2009) Development of 10 microsatellite loci in the ling (*Molva molva*). *Molecular Ecology Resources*, **9**, 1401-1403. doi:10.1111/j.1755-0998.2009.02677.x

Ritchie H, Cousins NJ, Cregeen SJ & Piertney SB (2013) Population genetic structure of the abyssal grenadier (*Coryphaenoides armatus*) around the mid-Atlantic ridge. *Deep Sea Research Part II: Topical Studies in Oceanography*, **98**, 431-437. doi:10.1016/j.dsr2.2013.06.014

Roberts DG & Ayre DJ (2010) Panmictic population structure in the migratory marine sparid *Acanthopagrus australis* despite its close association with estuaries. *Marine Ecology Progress Series*, **412**, 223-230. doi:10.3354/meps08676

Robinson N, Skinner A, Sethuraman L, McPartlan H, Murray N, Knuckey I, Smith DC, Hindell J & Talman S (2008) Genetic stock structure of blue-eye trevalla (*Hyperoglyphe antarctica*) and warehous (*Seriolella brama* and *Seriolella punctata*) in south-eastern Australian waters. *Marine and Freshwater Research*, **59**, 502-514. doi:10.1071/MF07175

Rocha LA (2004) Mitochondrial DNA and color pattern variation in three Western Atlantic *Halichoeres* (Labridae), with the revalidation of two species. *Copeia*, **4**, 770-782. doi:10.1643/CG-04-106

Rocha LA, Rocha CR, Robertson DR & Bowen BW (2008) Comparative phylogeography of Atlantic reef fishes indicates both origin and accumulation of diversity in the Caribbean. *BMC Evolutionary Biology*, **8**, 157. doi:10.1186/1461-2148-8-157

Rocha-Olivares A & Vetter RD (1999) Effects of oceanographic circulation on the gene flow, genetic structure, and phylogeography of the rosethorn rockfish (*Sebastes helvomaculatus*). *Canadian Journal of Fisheries and Aquatic Sciences*, **56**, 803-813. doi:10.1.509.409

Rocha-Olivares A & Sandoval-Castillo JR (2003) Mitochondrial diversity and genetic structure in allopatric populations of the Pacific red snapper *Lutjanus peru*. *Ciencias Marinas*, **29**, 197-205.

Rogers AD, Morley S, Fitzcharles E, Jarvis K & Belcher M (2006) Genetic structure of Patagonian toothfish (*Dissostichus eleginoides*) populations on the Patagonian Shelf and Atlantic and western Indian Ocean sectors of the Southern Ocean. *Marine Biology*, **149**, 915-92. doi:10.1007/s00227-006-0256-x

Roques S, Duchesne P & Bernatchez L (1999) Potential of microsatellites for individual assignment: The North Atlantic redfish (genus *Sebastes*) species complex as a case study. *Molecular Ecology*, 8, 1703-1717. doi:10.1046/j.1365-294x.1999.00759.x

Roques S, Sévigny J & Bernatchez L (2001) Evidence for broadscale introgressive hybridization between two redfish (genus *Sebastes*) in the north-west Atlantic: A rare marine example. *Molecular Ecology*, **10**, 149-165. doi:10.1046/j.1365-294X.2001.01195.x

Roques S, Galarza JA, MacPherson, Turner GF & Rico C (2007) Isolation and characterization of nine polymorphic microsatellite markers in the two-banded sea bream (*Diplodus vulgaris*) and cross-species amplification in the white sea bream (*Diplodus sargus*) and the saddled bream (*Oblada melanura*). *Molecular Ecology Notes*, **7**, 661-663. doi:10.1111/j.1471-8286.2006.01667.x

Roy D, Paterson IG, Hurlbut TR & Ruzzantel DE (2010) Development and design of five multi-locus microsatellite PCR panels for population genetic surveys of white hake (*Urophycis tenuis*) in the Northwest Atlantic. *Conservation Genetics Resources*, **2**, 45-49. doi:10.1007/s12686-009-9140-6

Roy EM, Quattro JM & Greig TW (2012) Genetic management of black sea bass: Influence of biogeographic barriers on population structure. *Marine and Coastal Fisheries: Dynamics, Management, and Ecosystem Science*, **4**, 391-402. doi:10.1080/19425120.2012.675983

Ruggeri P, Splendiani A, Bonanomi S, Arneri E, Cingolani N, Santojanni A, Belardinelli A, Giovannotti M & Barucchi VC (2012) Temporal genetic variation as revealed by a microsatellite analysis of European sardine (*Sardina pilchardus*) archived samples. *Canadian Journal of Fisheries and Aquatic Sciences*. **69**, 1698-1709. doi:10.1139/f2012-092

Ruggeri P, Splendiani A, Bonanomi S, Arneri E, Cingolani N, Santojanni A, Colella S, Donato F, Giovannotti M & Barucchi VC (2013) Searching for a stock structure in *Sardina pilchardus* from the Adriatic and Ionian seas using a microsatellite DNA-based approach. *Scientia Marina*, **77**, 565-574.

Ruggeri P, Splendiani A, Di Muri C, Fioravanti T, Santojanni A, Leonori I, De Felice A, Biagiotti I, Carpi P, Arneri E, Cerioni PN, Giovannotti M & Barucchi VC (2016b) Coupling demographic and genetic variability from archived collections of European anchovy (*Engraulis encrasicolus*). *PLoS ONE*, **11**, e0151507. doi:10.1371/journal.pone.015150

Ruggeri P, Splendiani A, Giovannotti M, Fioravanti T, Occhipinti G, Cerioni PN, Barucchi VC (2016a) The role of life-history traits, selective pressure and hydrographic boundaries in shaping the genetic structure of the transparent goby, *Aphia minuta*. *Marine Ecology*, **37**, 518-531. doi:10.1111/maec.12266

Ruzzante DE, Taggart CT, Cook D (1996a) Spatial and temporal variation in the genetic composition of a larval cod (*Gadus morhua*) aggregation: Cohort contribution and genetic stability. *Canadian Journal of Fisheries and Aquatic Sciences*, **53**, 2695-2705. doi:10.1139/f96-235

Ruzzante DE, Taggart CT, Cook D & Goddaard SV (1996) Genetic differentiation between inshore and offshore Atlantic cod (*Gadus morhua*) off Newfoundland: Microsatellite DNA variation and antifreeze level. *Canadian Journal of Fisheries and Aquatic Sciences*, **53**, 634-645. doi:10.1139/f95-228

Ruzzante DE, Taggart CT, Cook D & Goddaard SV (1997) Genetic differentiation between inshore and offshore Atlantic cod (*Gadus morhua*) off Newfoundland: A test and evidence of temporal stability. *Canadian Journal of Fisheries and Aquatic Sciences*, **54**, 27000-2708. doi:10.1139/f97-170

Ruzzante DE, Taggart CT & Cook D (1998) A nuclear DNA basis for shelf- and bank-scale population structure in northwest Atlantic cod (*Gadus morhua*): Labrador to Georges Bank. *Molecular Ecology*, **7**, 1663-1680. doi:10.1046/j.1365-294x.1998.00497.x

Ruzzante DE, Wroblewski JS, Taggart CT, Smedbol RK, Cook D & Goddaard SV (2000) Bay-scale population structure in coastal Atlantic cod in Labrador and Newfoundland, Canada. *Journal of Fish Biology*, **56**, 431-447. doi:10.1111/j.1095-8649.2000.tb02116.x

Ruzzante DE, Taggart CT, Doyle RW & Cook D (2001) Stability in the historical pattern of genetic structure of Newfoundland cod (*Gadus morhua*) despite the catastrophic decline in population size from 1964 to 1994. *Conservation Genetics*, **2**, 257-269.

Saarman NP, Louie KD & Hamilton H (2010) Genetic differentiation across eastern Pacific oceanographic barriers in the threatened seahorse *Hippocampus ingens*. *Conservation Genetics*, **11**, 1989-2000. doi:10.1007/s10592-010-0092-x

Saenz-Agudelo P, Jones GP, Thorrold SR & Planes S (2012) Patterns and persistence of larval retention and connectivity in a marine fish metapopulation. *Molecular Ecology*, **21**, 4695-4705. doi:10.1111/j.1365-294X.2012.05726.x

Sahyoun R, Guidetti P, Di Franco A & Planes S (2016) Patterns of fish connectivity between a marine protected area and surrounding fished areas. *PLoS ONE*, **11**, e0167441. doi:10.1371/journal.pone.0167441

Saillant E, Mousseau TA & Gold JR (2003) Genetic variation and relatedness of juvenile red snapper sampled from shrimp trawls in the Northern Gulf of Mexico. *Transactions of the American Fisheries Society*, **132**, 1229-1235. doi:10.1577/T03-006

Saillant E, Bradfield SC & Gold JR (2006) Genetic impacts of shrimp trawling on red snapper (*Lutjanus campechanus*) in the northern Gulf of Mexico. *ICES Journal of Marine Science*, **63**, 705-713. doi:10.1016/j.icesjms.2005.12.005

Saillant E & Gold JR (2006) Population structure and variance effective size of red snapper (*Lutjanus campechanus*) in the northern Gulf of Mexico. *Fishery Bulletin*, **104**, 136-148.

Saillant E, Bradfield SC & Gold JR (2010) Genetic variation and spatial autocorrelation among young-of-the-year red snapper (*Lutjanus campechanus*) in the northern Gulf of Mexico. *ICES Journal of Marine Science*, **67**, 1240-1250. doi:10.1093/icesjms/fsq011

Sala-Bozano M, Ketmaier V & Mariani S (2009a) Contrasting signals from multiple markers illuminate population connectivity in a marine fish. *Molecular Ecology*, **18**, 4811-4826. doi:10.1111/j.1365-294X.2009.04404.x

Sala-Bozano M, Tsalavouta M & Mariani S (2009b) Isolation and characterisation of new polymorphic microsatellite markers for the striped sea bream (*Lithognathus mormyrus*). *Conservation Genetics*, **10**, 1507. doi:10.1007/s10592-008-9770-3

Salas E, Molina-Ureña H, Walter RP & Heath DD (2010) Local and regional genetic connectivity in a Caribbean coral reef fish. *Marine Biology*, **157**, 437-445. doi:10.1007/s00227-009-1330-y

Saito T, Washio S, Dairiki K, Shimojo M, Itoi S & Sugita H (2008) High gene flow in *Girella punctata* (Perciformes, Kyphosidae) among the Japanese Islands inferred from partial sequence of the control region in mitochondrial DNA. *Journal of Fish Biology*, **73**, 1937-1945. doi:10.1111/j.1095-8649.2008.01997.x

Santa Brígada EL, Cunha DB, Regos PS, Sampaio I, Schneider H & Vallinoto M (2007) Population analysis of *Scomberomorus cavalla* (Cuvier, 1829) (Perciformes, Scombridae) from the Northern and Northeastern coast of Brazil. *Brazilian Journal of Biology*, **67**, 919-924.

Santos S, Hrbek T, Farias IP, Schneider H & Sampaio I (2006) Population genetic structuring of the king weakfish, *Macrodon ancylodon* (Sciaenidae), in Atlantic coastal waters of South America: Deep genetic divergence without morphological change. *Molecular Ecology*, **15**, 4361-4373. doi:10.1111/j.1365-294X.2006.03108.x

Santos MD, Lopez GV & Barut NC (2010) A pilot study on the genetic variation of eastern little tuna (*Euthynnus affinis*) in Southeast Asia. *Philippine Journal of Science*, **139**, 43-50.

Sato M, Kurokochi H, Tan E, Asakawa S, Hondra K, Bolisay KO, Nakamura Y, Lian C, Fortes MD & Nakaoka M (2014) Fifteen novel microsatellite markers for two *Amphiprion* species (*Amphiprion frenatus* and *Amphiprion perideraion*) and cross-species amplification. *Conservation Genetics Resources*, **6**, 685-688. doi:10.1007/s12686-014-0182-z

Sato M, Hondra K, Uy WH, Baslot DI, Genoiva TG, Nakamura Y, Bernardo LPC, Kurokochi H, Pantallano ADS, Lian C, Nadaoka K & Nakoaka M (2017) Marine protected area restricts demographic connectivity: Dissimilarity in a marine environment can function as a biological barrier. *Ecology and Evolution*, **7**, 7859-7871. doi:10.1002/ece3.3318

Sato M, Kitanishi S, Ishii M, Hamaguchi M, Kikuchi K & Hori M (2018) Genetic structure and demographic connectivity of marbled flounder (*Pseudopleuronectes yokohamae*) populations of Tokyo Bay. *Journal of Sea Research*, **142**, 79-90. doi:10.1016/j.seares.2018.09.019

Sbrocco EJ & Barber PH (2011) Ten polymorphic microsatellite loci for the Atlantic silverside, *Menidia menidia*. *Conservation Genetics Resources*, **3**, 585-587. doi:10.1007/s12686-011-9410-y

Schmidt C (2005) *Molecular genetic studies on species and population structure of North Atlantic redfish (Genus* Sebastes*; Cuvier 1829)*, University of Hamburg.

Schunter C, Carreras-Carbonell J, MacPherson E, Tintoré J, Vidal-Vijande E, Pascual A, Guidetti P & Pascual M (2011b) Matching genetics with oceanography: Directional gene flow in a Mediterranean fish species. *Molecular Ecology*, **20**, 5167-5181. doi:10.1111/j.1365-294X.2011.05355.x

Schunter C, Carreras-Carbonell J, Planes S, Sala E, Ballesteros E, Zabala M, Harmelin J, Harmelin-Vivien M, Macpherson E & Pascual M (2011a) Genetic connectivity patterns in an endangered species: The dusky grouper (*Epinephelus marginatus*). *Journal of Experimental Marine Biology and Ecology*. **401**, 126-133. doi:10.1016/j.jembe.2011.01.021

Scoles DR, Collette BB & Graves JE (1998) Global phylogeography of mackerels of the genus *Scomber*. *Fishery Bulletin*, **96**, 823-842.

Sebastian W, Sukumaran S, Zacharia PU & Gopalakrishnan (2017) Genetic population structure of Indian oil sardine, *Sardinella longiceps* assessed using microsatellite markers. *Conservation Genetics*, **18**, 951-964. doi:10.1007/s10592-017-0946-6

Šegvić-Bubić T, Lepen I, Trumbić Ž, Ljubković J, Sutlović D, Matić-Skoko S, Grubišić L, Glamuzina B & Mladineo I (2011) Population genetic structure of reared and wild gilthead sea bream (*Sparus aurata*) in the Adriatic Sea inferred with microsatellite loci. *Aquaculture*, **318**, 309-315. doi:10.1016/j.aquaculture.2011.06.007

Šegvić-Bubić T, Marrone F, Grubišić L, Izquierdo-Gomez D, Katavić I, Arculeo M & Brutto SL (2016) Two seas, two lineages: How genetic diversity is structured in Atlantic and Mediterranean greater amberjack *Seriola dumerili* Risso, 1810 (Perciformes, Carangidae). *Fisheries Research*, **179**, 271-279. doi:10.1016/j.fishres.2016.03.018

Sekino M & Hara M (2001) Application of microsatellite markers to population genetics studies of Japanese flounder *Paralichthys olivaceus*. *Marine Biotechnology*, **3**, 572-589. doi:10.1007/s10126-001-0064-8

Sekino M, Saitoh K, Shimizu D, Wada T, Kamiyama K, Gambe S, Chen S & Aritaki M (2011) Genetic structure in species with shallow evolutionary lineages: a case study of the rare flatfish *Verasper variegatus*. *Conservation Genetics*, **12**, 139-159. doi:10.1007/s10592-010-0128-2

Selkoe KA, Gaines SD, Caselle JE & Warner R (2006) Current shifts and kin aggregation explain genetic patchiness in fish recruits. *Ecology*, **87**, 3082-3094. doi:10.18900/0012-9658(2006)87[3082:CSAKAE]2.0.CO;2

Sellas AB, Bassos-Hull K, Hueter RE & Feldheim KA (2011) Isolation and characterization of polymorphic microsatellite markers from the spotted eagle ray (*Aetobatus narinari*). *Conservation Genetics Resources*, **3**, 609-611. doi:10.1007/s12686-011-9415-6

Sellas AB, Bassos-Hull K, Pérez-Jiménez JC, Angulo-Valdés JA, Bernal MA & Hueter RE (2015) Population structure and seasonal migration of the spotted eagle ray, *Aetobatus narinari*. *Journal of Heredity*, **106**, 266-275. doi:10.1093/jhered/esv011

Semenova AV, Stroganov AN, Afanasiev KI & Rubtsova GA (2015) Population structure and variability of Pacific herring (*Clupea pallasii*) in the White Sea, Barents and Kara Seas revealed by microsatellite DNA analyses. *Polar Biology*, **38**, 951-965. doi:10.1007/s00300-015-1653-8

Semenova AV, Stroganov AN, Afanasiev KI, Rubtsova GA, Zhukova KA & Smirnov AA (2018) Microsatellite variability of Pacific herring *Clupea pallasii* Valenciennes, 1847 from the Sea of Okhotsk and Bering Sea. *Russian Journal of Genetics*, **54**, 335-345.

Sepúlveda FA & González MT (2017) Spatio-temporal patterns of genetic variations in populations of yellowtail kingfish *Seriola lalandi* from the south-eastern Pacific Ocean and potential implications for its fishery management. *Journal of Fish Biology*, **90**, 249-264. doi:10.1111/jfb.13179

Seyoum S, Denison SH & Tringali MD (2007) Isolation and characterization of 13 polymorphic microsatellite loci for the Florida pompano, *Trachinotus carolinus*. *Molecular Ecology Notes*, **7**, 141-143. doi:10.1111/j.1471-8286.2006.01556.x

Seyoum S, McBride RS, Puchutulegul C, Dutka-Gianelli J, Alvarez AC & Panzer K (2017) Genetic population structure of sheepshead, *Archosargus probatocephalus* (Sparidae), a coastal marine fish off the southeastern United States: multiple population clusters based on species-specific microsatellite markers. *Bulletin of Marine Science*, **93**, 691-713. doi:10.5343/bms.2016.1069

Sha Z, Xing S, Shao C, Tian Y, Liao X & Chen S (2009) Isolation and characterization of 12 polymorphic microsatellite markers from ladyfish (*Elops saurus Linnaeus*). *Conservation Genetics*, **10**, 1799. doi:10.1007/s10592-009-9819-y

Shaw PW, Turan C, Wright JM, O'Connell M & Carvalho GR (1999) Microsatellite DNA analysis of population structure in Atlantic herring (*Clupea harengus*), with direct comparison to allozyme and mtDNA RFLP analyses. *Heredity*, **83**, 490-499.

Shaw PW, Arkhipkin AI & Al-Khairulla H (2004) Genetic structuring of Patagonian toothfish populations in the Southwest Atlantic Ocean: The effect of the Antarctic PolarFront and deep-water troughs as barriers to genetic exchange. *Molecular Ecology*, **13**, 3293-3303. doi:10.1111/j.1365-294X.2004.02327.x

Sherman KD, Paris JR, King RA, Moore KA, Dahlgren CP, Knowles LC, Stump K, Tyler CR & Stevens JR (2017) RAD-seq analysis and i*n situ* monitoring of Nassau grouper reveal fine-scale population structure and origins of aggregating fish. *Frontiers in Marine Science*, **4**, 393. doi:10.3389/fmars.2020.00157

Shigenobu Y, Hayashizaki K-I, Asahida T, Ida H & Saitoh K (2007) Stock structure of Japanese flounder inferred from morphological and genetic analyses. *Fisheries Science*, **73**, 1104-1112. doi:10.1111/j.1444-2906.2007.01442.x

Shishidou H, Kitada S, Sakamoto T & Hamasaki K (2008) Genetic variability of wild and hatchery-released red sea bream in Kagoshima Bay, Japan, evaluated by using microsatellite DNA analysis. *Nippon Suisan Gakkaishi*, **74**, 183-188.

Shubina EA, Ponomareva EV & Glubokov AI (2009) Population genetic structure of walleye pollock *Theragra chalcogramma* (Gadidae, Pisces) from the Bering Sea and Sea of Okhotsk. *Molecular Biology*, **43**, 855. doi:10.1134/S0026893309050262

Shui B, Han Z, Gao T, Miao Z & Yanagimoto T (2009) Mitochondrial DNA variation in the East China Sea and Yellow Sea populations of Japanese Spanish mackerel *Scomberomorus niphonius*. *Fisheries Science*, **75**, 593-600. doi:10.1007/s12562-009-0083-3

Shulzitski K (2005)*A genetic assessment of population connectivity in mutton snapper, Lutjanus analis*, University of Carolina, Wilmington.

Siegle MR, Taylor EB, Miller KM, Withler RE & Yamanaka KL (2013) Subtle population genetic structure in yelloweye rockfish (*Sebastes ruberrimus*) is consistent with a major oceanographic division in British Columbia, Canada. *PLoS ONE*, **8**, e71083. doi:10.1371/journal.pone.0071083

Silva G, Horne JB & Castilho R (2014) Anchovies go north and west without losing diversity: Post-glacial range expansions in a small pelagic fish. *Journal of Biogeography*, **41**, 1171-1182. doi:10.1111/jbi.12275

Silva G, Cunha RL, Ramos A & Castilho R (2017) Wandering behaviour prevents inter and intra oceanic speciation in a coastal pelagic fish. *Scientific Reports*, **7**, 2893.

Silva D, Martins K, Oliveira J, da Silva R, Sampaio I, Schneider H & Gomes G (2018) Genetic differentiation in populations of lane snapper (*Lutjanus synagris* – Lutjanidae) from Western Atlantic as revealed by multilocus analysis. *Fisheries Research*, **198**, 138-149. doi: 10.1016/j.fishres.2017.10.005

Sims CA, Riginos C, Blomberg SP, Huelsken T, Drew J & Grutter AS (2014) Cleaning up the biogeography of *Labroides dimidiatus* using phylogenetics and morphometrics. *Coral Reefs*, **33**, 223-233. doi:10.1007/s00338-013-1093-2

Skarstein TH, Westgaard J-I & Fevolden S-E (2007) Comparing microsatellite variation in north-east Atlantic cod (*Gadus morhua* L.) to genetic structuring as revealed by the pantophysin (*Pan* I) locus. *Journal of Fish Biology*, **70**, 271-290. doi:10.1111/j.1095-8649.2007.01456.x

Small MP, Loxterman JL, Frye AE, Von Bargen JF, Bowman C & Young SF (2005) Temporal and spatial genetic structure among some Pacific herring populations in Puget Sound and the Southern Strait of Georgia. *Transactions of the American Fisheries Society*, **134**, 1329-1341. doi:10.1577/T05-050.1

Smith P & McVeagh M (2000) Allozyme and microsatellite DNA markers of toothfish population structure in the Southern Ocean. *Journal of Fish Biology*, **57**, 72-83. doi:10.1111/j.1095-8649.2000.tb02245.x

Song N, Ma G, Zhang X, Gao T & Sun D (2014) Genetic structure and historical demography of *Collichthys lucidus* inferred from mtDNA sequence analysis. *Environmental Biology of Fishes*, **97**, 69-77. doi:10.1007/s10641-013-0124-8

Song N, Liu M, Yanagimoto T, Sakurai Y, Han Z & Gao T (2016) Restricted gene flow for *Gadus macrocephalus* from Yellow Sea based on microsatellite markers: Geogrpahic block of Tsushima current. *International Journal of Molecular Sciences*, **17**, 467. doi:10.3390/ijms17040467

Sorenson L, McDowell JR & Graves JE (2011) Isolation and characterization of microsatellite markers for blue marlin, *Makaira nigricans*. *Conservation Genetics Resources*, **3**, 721-723. doi:10.1007/s12686-011-9441-4

Sotka EE, Hempelmann JA & Biermann CH (2005) Genetic evidence of postglacial population expansion in Puget Sound rockfish (*Sebastes emphaeus*). *Marine Biotechnology*, **7**, 223-230. doi:10.1007/s10126-004-0437-x

Spies IB, Lowe S, Hong Y & Canino MF (2005) Development and characterization of seven novel di-, tri-, and tetranucleotide microsatellite markers in Atka mackerel (*Pleurogrammus monopterygius*). *Molecular Ecology Notes*, **5**, 469-471. doi:10.1111/j.1471-8286.2005.00947.x

Spies I (2012) Landscape genetics reveals population subdivision in Bering Sea and Aleutian Islands Pacific cod. *Transactions of the American Fisheries Society*, **141**, 1557-1573. doi:10.1080/00028487.2012.711265

Stefanni S, Castilho R, Sala-Bozano M, Robalo JI, Francisco SM, Santos RS, Marques N, Brito A, Almada VC & Mariani S (2015) Establishment of a coastal fish in the Azores: Recent colonisation or a sudden expansion of an ancient relict population? *Heredity*, **115**, 527-537.

Stefánsson MÖ, Reinert J, Sigurðsson Þ, Kristinsson K, Nedreaas K & Pampoulie C (2009a) Depth as a potential driver of genetic structure of *Sebastes mentella* across the North Atlantic Ocean. *ICES Journal of Marine Science*, **66**, 680-690. doi:10.1093/icesjms/fsp059

Stefánsson MÖ, Sigurdsson T, Pampoulie C, Daníelsdóttir AK, Thorgilsson B, Ragnardsdóttir A, Gíslason DG, Coughlan J, Cross TF & Bernatchez L (2009b) Pleistocene genetic legacy suggests incipient species of *Sebastes mentella* in the Irminger Sea. *Heredity*. **102**, 514-524.

Steinberg R, van der Meer M, Walker E, Berumen ML, Hobbs JA & van Herwerden L (2016) Genetic connectivity and self-replenishment of inshore and offshore populations of the endemic anemonefish, *Amphiprion latezonatus*. **35**, 959-970. doi:10.1007/s00338-016-1420-5

Stepien CA (1999) Phylogeographical structure of the Dover sole *Microstomus pacificus*: The larval retention hypothesis and genetic divergence along the deep continental slope of the northeastern Pacific Ocean. *Molecular Ecology*, **8**, 923-939. doi:10.1046/1365-294x.1999.00643.

Stockley B, Menezes G, Pinho MR & Rogers AD (2005) Genetic population structure in the black-spot sea bream (*Pagellus bogaraveo* Brünnich, 1768) from the NE Atlantic. *Marine Biology*, **146**, 793-804. doi:10.1007/d00227-004-1479-3

Stroganov AN, Orlov AM, Buryakova ME & Afanas'ev KI (2009) On genetic differentiation of the Pacific cod *Gadus macrocephalus* tilesius, 1810 (Gadiformes: Gadidae). *Russian Journal of Marine Biology*, **35**, 490-493. doi:10.1134/S1063074009060066

Sun P & Tang BJ (2018) Low mtDNA variation and shallow population structure of the Chinese pomfret *Pampus chinensis* along the China coast. *Journal of Fish Biology*, **92**, 214-228. doi:10.1111/jfb.13515

Sun D, Ge Y & Cheng Q (2019) Genetic diversity of eight wild populations of *Pampus argenteus* along the coast of China inferred from fifteen polymorphic microsatellite markers. *Brazilian Journal of Oceanography*, **67**, e19251. doi:10.1590/S1679-87592019025106711

Susana E, Papetti C, Barbisan F, Bortolotto E, Buccoli S, Patarnello T & Zane L (2007) Isolation and characterization of eight microsatellite loci in the icefish *Chaenocephalus aceratus* (Perciformes, Notothenioidei, Channichthyidae). *Molecular Ecology Notes*, **7**, 791-793. doi:10.1111/j.1471-8286.2007.01703.x

Taillebois L, Barton DP, Crook DA, Saunders T, Taylor J, Hearnden M, Saunders RJ, Newman SJ, Travers MJ, Welch DJ, Greig A, Dudgeon C, Maher S & Ovenden JR (2017) Strong population structure deduced from genetics, otolith chemistry and parasite abundances explain vulnerability to localized fishery collapse in a large Sciaenid fish, *Protonibea diacanthus*. *Evolutionary Applications*, **10**, 978-993. doi:10.1111/eva.12499

Takagi M, Okamura T, Chow S & Taniguchi N (1999b) PCR primers for microsatellite loci in tuna species of the genus *Thunnus* and its application for population genetic study. *Fisheries Science*, **65**, 571-576. doi:10.2331/fishsci.65.571

Takagi M, Yoshida K & Taniguchi N (1999a) Isolation of microsatellite loci from Japanese flounder *Paralichthys olivaceus* and detection of PCR fragments with simple non-RI methods. *Fisheries Science*, **65**, 486-487. doi:10.2331/fishsci.65.486

Takagi M ,Okamura T, Chow S & Taniguchi N (2001) Preliminary study of albacore (*Thunnus alalunga*) stock differentiation inferred from microsatellite DNA analysis. *Fishery Bulletin-National Oceanic and Atmospheric Administration*, **99.4**, 697-701.

Tang CY, Tzeng CH, Chen CS & Chiu TS (2009) Microsatellite DNA markers for population-genetic studies of blue mackerel (*Scomber australasicus*) and cross-specific amplification in *S. japonicus*. *Molecular Ecology Resources*, **9**, 824-827. doi:10.1111/j.1755-0998.2008.02278.x

Tanner SE, Pérez M, Presa P, Thorrold SR & Cabral HN (2014) Integrating microsatellite DNA markers and otolith geochemistry to assess Population structure of European hake (*Merluccius merluccius*). *Estuarine, Coastal and Shelf Science*, **142**, 68-75. doi:10.1016/j.ecss.2014.03.010

Teacher AGF, André C, Jonsson PR & Merilä J (2013) Oceanographic connectivity and environmental correlates of genetic structuring in Atlantic herring in the Baltic Sea. *Evolutionary Applications*, **6**, 549-567. doi:10.1111/eva.12042

Teske PR, Hamilton H, Palsbell PJ, Choo CK, Gabr H, Lourie SA, Santos M, Sreepada A, Cherry MI & Matthee CA (2005) Molecular evidence for long-distance colonization in an Indo-Pacific seahorse lineage. *Marine Ecology Progress Series*, **286**, 249-260. doi:10.3354/meps286249

Teske PR, Cowley PD, Forget FRG & Beheregaray LB (2009) Microsatellite markers for the roman, *Chrysoblephus laticeps* (Teleostei: Sparidae), an overexploited seabream from South Africa. *Molecular Ecology Resources*, **9**, 1162-1164. doi:10.1111/j.1755-0998.2009.02595.x

Teske PR, Forget FRG, Cowley PD, von der Heyden S & Beheregaray LB (2010) Connectivity between marine reserves and exploited areas in the philopatric reef fish *Chrysoblephus laticeps* (Teleostei: Sparidae). *Marine Biology*, **157**, 2092-2042. doi:10.1007/s00227-010-1471-z

Thacker CE, Thompson AR, Roje DM & Shaw EY (2008) New expansions in old clades: Population genetics and phylogeny of *Gnatholepis* species (Teleostei; Gobioidei) in the Pacific. *Marine Biology*, **153**, 275-285. doi:10.1007/s00227-007-0814-x

Thornburn J, Jones R, Neat F, Pinto C, Bendall V, Hetherington S, Bailey M, Leslie N & Jones C (2018) Spatial versus temporal structure: Implications of inter-haul variation and relatedness in the North-east Atlantic spurdog *Squalus acanthias*. *Aquatic Conservation: Marine and Freshwater Ecosystems*, **28**, 1167-1180. doi:10.1002/aqc.2922

Tian Y-S, Miao G-D, Shao C-W, Liao X-L & Chen S-L (2009) Isolation and characterization of polymorphic microsatellite loci from a repeat-enriched genomic library of stone flounder (*Kareius bicoloratus*) and cross-species amplification. *Conservation Genetics*, **10**, 1041-1043. doi:10.1007/s10592-008-9683-1

Timm J, Planes S & Kochzius M (2012) High similarity of genetic population structure in the false clown anemonefish (*Amphiprion ocellaris*) found in microsatellite and mitochondrial control region analysis. *Conservation Genetics*, **13**, 693-706. doi:10.1007/s10592-012-0318-1

Timm J, Kochzius M, Madduppa HH, Neuhaus AI & Dohna T (2017) Small scale genetic population structure of coral reef organisms in Spermonde Archipelago, Indonesia. *Frontiers in Marine Science*, **4**, 294. doi:10.3389/fmars.2017.00294

Tohkairin A, Kai Y, Ueda Y, Hamatsu T, Ito M & Nakabo T (2016) Genetic population structure *Crystallichthys matsushimae* (Cottoidei: Liparidae) with comments on color variation. *Ichthyological Research*, **63**, 370-381. doi:10.1007/s10228-015-0507-2

Tripp-Valdez MA, García de León FJ, Ortega-García S, Lluch-Cota D, López-Martínez J & Cruz P (2010) Population genetic structure of dolphinfish (*Corpyphaena hippurus*) in the Gulf of California, using microsatellite loci. *Fisheries Research*, **105**, 172-177. doi:10.1016/j.fishres.2010.03.023

Tripp-Valdez MA, García-de-León FJ, Espinosa-Pérez H & Ruiz-Campos G (2012) Population structure of sablefish *Anoplopoma fimbria* using genetic variability and geometric morphometric analysis. *Journal of Applied Ichthyology*, **28**, 516-523. doi:10.1111/j.1439-0426.2012.01942.x

Tseng MC, Jean CT, Tsai WL & Chen NC (2009) Distinguishing between two sympatric *Acanthopagrus* species from Dapeng Bay, Taiwan, using morphometric and genetic characters. *Journal of Fish Biology*, **74**, 357-376. doi:10.1111/j.1095-8649.2008.02049.x

Tu Z, Liu M, Wang Y, Xu S, Song N, Gao T, Han Z (2016) The low mitochondrial diversities in lizardfish *Saurida elongate*: recent population expansion and selection. *Biochemical Systematics and Ecology*, **68**, 44-50. doi:10.1016/j.bse.2016.06.011

Turan C (2015) Microsatellite DNA reveals genetically different populations of Atlantic bonito *Sarda sarda* in the Mediterranean basin. *Biochemical Systematics and Ecology*, **63**, 174-182. doi:10.1016/j.bse.2015.10.007

Turner TF, Richardson LR & Gold JR (1998) Polymorphic microsatellite DNA markers in red drum (*Sciaenops ocellatus*). *Molecular Ecology*, **7**, 1771-1773.

Tysklind N, Taylor MI, Lyons BP, McCarthy ID & Carvalho GR (2009) Development of 30 microsatellite markers for dab (*Limanda limanda* L.): A key UK marine biomonitoring species. *Molecular Ecology Resources*, **9**, 951-955. doi:10.1111/j.1755-0998.2008.02513.x

Tysklind N, Taylor MI, Lyons BP, Goodsir F, McCarthy ID & Carvalho GR (2013) Population genetics provides new insights into biomarker prevalence in dab (*Limanda limanda* L.): A key marine biomonitoring species. *Evolutionary Applications*, **6**, 891-909. doi:10.1111/eva.12074

Tzeng T (2007) Population structure and historical demography of the spotted mackerel (*Scomber australasicus*) off Taiwan inferred from mitochondrial control region sequencing. *Zoological Studies*, **46**, 656-663.

Tzeng T, Huang H, Wang D & Yeh S (2007) Genetic diversity and population expansion of the common mackerel (*Scomber japonicus*) off Taiwan. *Journal of the Fisheries Society of Taiwan*, **34**, 237-245.

Ueno K, Watanabe M, Ahmad-Syazni K, Koike M, Ohara K & Umino T (2013) Eleven novel microsatellite loci for Japanese whiting (*Sillago japonica*) and cross amplification in the endangered small-scale sillago (*Sillago parvisquamis*). *Conservation Genetics Resources*, **5**, 659-662. doi:10.1007/s12686-013-9876-x

Umino T, Kajihara T, Shiozaki H, Ohkawa T, Jeong D & Ohara K (2009) Wild stock structure of *Girella punctata* in Japan revealed shallow genetic differentiation but subtle substructure in subsidiary distributions. *Fisheries Science*, **75**, 909-919. doi:10.1007/s12562-009-0118-

Umino T, Ueno K, Mihara T, Koike M, Watanabe M, Ahmad-Syazni K, Ishitani M & Ohara K (2013) Isolation of eleven polymorphic microsatellite loci for the endangered *Sillago parvisquamis* and cross-species amplification with *Sillago japonica*. *Conservation Genetics Resources*, **5**, 771-773. doi:10.1007/s12686-013-9904-x

Underwood JN (2009) Characterisation of ten polymorphic microsatellite loci in a coral reef fish (*Chromis margaritifer*). *Conservation Genetics Resources*, **1**, 491. doi:10.1007/s12686-009-9114.8

Underwood JN (2010) Development and optimisation of ten novel microsatellite loci I nthree multiplex PCR panels for the Wolfie cardinalfish (*Cheilodipterus artus*). *Conservation Genetics Resources*, **2**, 261-264. doi:10.1007/s12686-010-9182-9

Underwood JN, Travers MJ & Gilmour JP (2012) Subtle genetic structure reveals restricted connectivity among populations of a coral reef fish inhabiting remote atolls. *Ecology and Evolution*, **2**, 666-679. doi:10.1002/ece3.80

Valentin AE, Penin X, Chanut J, Power D & Sévigny J (2014) Combining microsatellites and geometric morphometrics for the study of redfish, (*Sebastes*, spp.) population structure in the Northwest Atlantic. *Fisheries Research*, **154**, 102-119. doi:10.1016/j.fishres.2012.02.008

Valenzuela-Quiñonez F, Garza JC, De-Anda-Montañez JA & García-de-León FJ (2014) Inferring past demographic changes in a critically endangered marine fish after fishery collapse. *ICES Journal of Marine Science*, **71**, 1619-1628. doi:10.1093/icesjms/fsu058

Valenzuela-Quiñonez F, De-Anda-Montañez JA, Gilbert-Horvath E, Garza JC & García-De León FJ (2016) Panmixia in a critically endangered fish: The totoaba (*totoaba macdonaldi*) in the Gulf of California. *Journal of Heredity*, **107**, 496-503. doi:10.1093/jhered/esw046

Van de Putte AP, Van Houdt JKJ, Maes GE, Janko K, Koubbi P, Rock J & Volckaert FAM (2009) Species identification in the trematomid family using nuclear genetic markers. *Polar Biology*, **32**, 1731. doi:10.1007/s00300-009-0672-8

Van de Putte AP, Janko K, Kasparova E, Maes GE, Rock J, Koubbi P, Volckaert FAM, Choleva L, Fraser KPP, Smykla J, Van Houdt JKJ & Marshall C (2012b) Comparative phylogeography of three trematomid fishes reveals contrasting genetic structure patterns in benthic and pelagic species. *Marine Genomics*, **8**, 23-34. doi:10.1016/j.margen.2012.05.002

Van de Putte AP, Van Houdt JKJ, Maes GE, Hellemans B, Collins MA & Vockaert FAM (2012a) High genetic diversity and connectivity in a common mesopelagic fish of the Southern Ocean: The myctophid *Electrona antarctica*. *Deep Sea Research Part II: Topical Studies in Oceanography*, **59-60**, 199-207. doi:10.1016/j.dsr2.2011.05.011

van der Meer MH, Hobbs JA, Jones GP & van Herwerden L (2012) Genetic connectivity among and self-replenishment within island populations of a restricted range subtropical reef fish. *PLoS ONE*, **7**, e49660. doi:10.1371/journal.pone.0049660

van der Meer MH, Horne JB, Gardner MG, Hobbs JA, Pratchett M & van Herwerden L (2013a) Limited contemporary gene flow and high self-replenishment drives peripheral isolation in an endemic coral reef fish. *Ecology and Evolution*, **3**, 1653-1666. doi:10.1002/ece3.584

van der Meer MH, Gardner MG, Berumen ML, Hobbs J-PA & van Herwerden L (2013) Identification of seventeen microsatellite loci for conservation genetic studies of the endemic wrasse *Coris bulbifrons*. *Conservation Genetics Resources*, **5**, 363-366. doi:10.1007/s12686-012-9804-5

van der Meer MH, Berumen ML, Hobbs J-PA van Herwerden L (2015) Population connectivity and the effectiveness of marine protected areas to protect vulnerable, exploited and endemic coral reef fishes at an endemic hotspot. *Coral Reefs*, **34**, 393-402. Doi:10.1007/s00338-014-1242-2

Van Herwerden L, Benzie J & Davies C (2003) Microsatellite variation and population genetic structure of the red throat emperor on the Great Barrier Reef. *Journal of Fish Biology*, **62**, 987-999. doi:10.1046/j.1095-8649.2003.00075.x

Van Herwerden L, McIlwain J, Al-Oufi H, Al-Amry W & Reyes A (2006) Development and application of microsatellite markers for *Scomberomorus commerson* (Perciformes; Teleostei) to a population genetic study of Arabian Peninsula stocks. *Fisheries Research,* **79**, 256-266. doi:10.1016/j.fishres.2006.04.004

Van Herwerden L, Choat JH, Newman SJ, Leray M & Hillersøy (2009) Complex patterns of population structure and recruitment of *Plectropomus leopardus* (Pisces: Epinephehlidae) in the Indo-West Pacific: implications for fisheries management. *Marine Biology*, **156**, 1595-1607. doi:10.1007/s00227-009-1195-0

Van Houdt JKJ, Hellemans B, Van de Putte A, Koubbi P & Volckaert FAM (2006) Isolation and multiplex analysis of six polymorphic microsatellites in the Antarctic notothenioid fish, *Trematomus newnesi*. *Molecular Ecology Notes*, **6**, 157-159. doi:10.1111/j.1471-8286.2005.01174.x

Varela AI, Ritchie PA & Smith PJ (2012) Low levels of global genetic differentiation and population expansion in the deep-sea teleost *Hoplostethus atlanticus* revealed by mitochondrial DNA sequences. *Marine Biology*, **159**, 1049-1060. doi:10.1007/s00227-012-1885-x

Varela AI, Ritchie PA & Smith PJ (2013) Global genetic population structure in the commercially exploited deep-sea teleost orange roughy (*Hoplostethus atlanticus*) based on microsatellite DNA analyses. *Fisheries Research*, **140**, 83-90. doi:10.1016/j.fishres.2012.12.011

Vargas-Caro C, Bustamante C, Bennett MB & Ovenden JR (2017) Towards sustainable fishery management for skates in South America: The genetic population structure of *Zearaja chilensis* and *Dipturus trachyderma* (Chondrichthyes, Rajiformes) in the south-east Pacific Ocean. *PLoS ONE*, **12**, e0172255. doi:10.1371/journal.pone.0172255

Veilleux HD, van Herwerden L, Evans RD, Travers MJ & Newman SJ (2011) Strong genetic subdivision generates high genetic variability among eastern and western Australian populations of *Lutjanus carponotatus* (Richardson). *Fisheries Research*, **108**, 74-80. doi:10.1016/j.fishres.2010.11.026

Vella A (2009) Historical landing statistics, size, sex and microsatellite analyses of bluefin tuna (*Thunnus thynnus thynnus*) in the Central Southern Mediterranean Sea. *Collective Volumes of Scientific Papers ICCAT*, **63**, 161-173. doi:10.1.582.4001

Vella A, Vella N, Karakulak FS, Oray I, Garcia-Tiscar S & de Stephanis R (2016) Population genetics of Atlantic bluefin tuna, *Thunnus thynnus*, (Linnaeus, 1758), in the Mediterranean: Implications for its conservation management. *Journal of Applied Ichthyology*, **32**, 523-531. doi:10.1111/jai.13035

Vella A, Vella N & Schembri S (2017) A molecular approach towards taxonomic identification of elasmobranch species from Maltese fisheries landings. *Marine Genomics*, **36**, 17-23. doi:10.1016/j.margen.2017.08.008

Vignaud TM, Maynard JA, Leblois R, Meekan MG, Vázquez-Juárez R, Ramírez-Marcias D, Pierce SJ, Berumen ML, Beeravolu C, Baksay S & Planes S (2014) Genetic structure of populations of whale sharks among ocean basins and evidence for their historic rise and recent decline. *Molecular Ecology*, **23**, 2590-2601. doi:10.1111/mec.12754

Vilas R, Vandamme SG, Vera M, Bouza C, Maes GE, Volckaert FAM & Martínez P (2015) A genome scan for candidate genes involved in the adaptation of turbot (*Scophthalmus maximus*). *Marine Genomics*, **23**, 77-86. doi:10.1016/j.margen.2015.04.011

Viñas J, Gordoa A, Fernández-Cebrián R, Pla C, Vahdet Ü & Araguas RM (2011) Facts and uncertainties about the genetic population structure of Atlantic bluefin tuna (*Thunnus thynnus*) in the Mediterranean. Implications for fishery management. *Reviews in Fish Biology and Fisheries*, **21**, 527-541. doi:10.1007/s11160-010-9174-6

Viñas J, Sanz N, Peñarrubia L, Araguas R, García-Marín J, Roldán M & Pla C (2014) Genetic population structure of European anchovy in the Mediterranean Sea and the northeast Atlantic Ocean using sequence analysis of the mitochondrial DNA control region. *ICES Journal of Marine Science*, **71**, 391-397. doi:10.1093/icesjms/fst132

Viret A, Tsaparis D, Tsigenopoulos CS, Berrebi P, Sabatini A, Arculeo M, Fassatoui C, Magoulas A, Marengo M, Morales-Nin B, Caill-Milly N & Durieux EDH (2018) Absence of spatial genetic structure in common dentex (*Dentex dentex* Linnaeus, 1758) in the Mediterranean Sea as evidenced by nuclear and mitochondrial molecular markers. *PLoS ONE*, **13**, e0203866. doi:10.1371/journal.pone.0203866

Vis ML, Carr SM, Bowering WR & Davidson WS (1997) Greenland halibut (*Reinhardtius hippoglossoides*) in the North Atlantic are genetically homogeneous. *Canadian Journal of Fisheries and Aquatic Sciences*, **54**, 1813-1821. doi:10.1139/f97-088

Visram S, Yang M, Pillay RM, Said S, Henriksson O, Gahn M & Chen CA (2010) Genetic connectivity and historical demography of the blue barred parrotfish (*Scarus ghobban*) in the western Indian Ocean. *Marine Biology*, **157**, 1475-1487. doi:10.1007/s00227-010-1422-8

von der Heyden S, Lipinski MR & Matthee CA (2007) Mitochondrial DNA analyses of the Cape hakes reveal an expanding, panmictic population for *Merluccius capensis* and population structuring for mature fish in *Merluccius paradoxus*. *Molecular Phylogenetics and Evolution*, **42**, 517-527. doi:10.1016/j.ympev.2006.08.004

Waldrop E, Hobbs, JA, Randall JE, DiBattista JD, Rocha LA, Kosaki RK, Berumen ML & Bowen BW (2016) Phylogeography, population structure and evolution of coral-eating butterflyfishes (Family Chaetodontidae, genus *Chaetodon*, subgenus *Corallochaetodon*). *Journal of Biogeography*, **43**, 1116-1129. doi:10.1111/jbi.12680

Wang L, Meng Z, Fan B, Sang Q, Luo Y, Zhang Y, Liu X & Lin H (2010a) Molecular Ecology Resources Primer Development Consortium. Permanent genetic resources added to molecular ecology resources database, 1 August 2009-30 September 2009. *Molecular Ecology Resources*, **10**, 232-236. doi:10.1111/j.1755-0998.2009.02796.x

Wang L, Fan B, Zhuang Z, Meng Z, Zhang Y, Liu X & Lin H (2010b) Molecular Ecology Resources Primer Development Consortium. Permanent genetic resources added to molecular ecology resources database, 1 December 2009-31 January 2010. *Molecular Ecology Resources*, **10**, 576-579. doi:10.1111/j.1755-0998.2010.02851.x

Wang RX, Xu TJ, Sun YN & He GY (2010c) Polymorphic microsatellite loci from two enriched genomic libraries for the genetic analysis of the miiuy croaker, *Miichthys miiuy* (Sciaenidae). *Genetics and Molecular Research*, **9**, 931-934. doi:10.4238/vol9-2gmr806

Wang L, Shi X, Su Y, Meng Z & Lin H (2012) Loss of genetic diversity in the cultured stocks of the large yellow croaker, *Larimichthys crocea*, revealed by microsatellites. *International Journal of Molecular Sciences*, **13**, 5584-5597. doi:10.3390/ijms13055584

Wang L, Liu S, Zhuang Z, Guo L, Meng Z & Lin H (2013) Population genetic studies revealed local adaptation in a high gene-flow marine fish, the small yellow croaker (*Larimichthys polyactis*). *PLoS ONE*, **8**, e83493. doi:10.1371/journal.pone.0083493

Wang L, Liu S, Zhuang Z, Lin H & Meng Z (2015) Mixed-stock analysis of small yellow croaker *Larimichthys polyactis* providing implications for stock conservation and management. *Fisheries Research*, **161**, 86-92. doi:10.1016/j.fishres.2014.06.006

Wang Q, Wang X, Xie Z, Li Y, Xiao L, Peng C, Zhang H, Li S, Zhang Y & Lin H (2016) Microsatellite analysis of the genetic relationships between wild and cultivated giant grouper in the South China Sea. *Journal of Genetics*, **95**, 369-376. doi:10.1007/s12041-016-0647-9

Was A, Gosling E, McCrann K & Mork J (2008) Evidence for population structuring of blue whiting (*Micromesistius poutassou*) in the northeastern Atlantic. *ICES Journal of Marine Science*, **65**, 216-225. doi:10.1093/icesjms/fsm187

Was A, Gosling E & Hoarau G (2010) Microsatellite analysis of plaice (*Pleuronectes platessa* L.) in the NE Atlantic: Weak genetic structuring in a milieu of high gene flow. *Marine Biology*, **157**, 447-462. doi:10.1007/s00227-009-1331-x

Watts PC, Nash RDM, George SG & Kemp SJ (1999) Isolation and characterization of microsatellite loci in the European plaice, *Pleuronectes platessa* L. (Teleostei: Pleuronectidae). *Molecular Ecology*, **8**, 2151-2152. doi:10.1046/j.1365-294Ax.1999.00802-6.x

Watts PC, Nash RDM & Kemp SJ (2001) Polymorphic microsatellite loci in the European plaice, *Pleuronectes platessa*, and their utility in flounder, lemon sole and Dover sole. *Journal of the Marine Biological Association of the United Kingdom*, **81**, 367-368.

Watts PC, Nash RDM & Kemp SJ (2004) Genetic structure of juvenile plaice *Pleuronectes platessa* on nursery grounds within the Irish Sea. *Journal of Sea Research*, **51**, 191-197. doi:10.1016/j.seares.2003.09.003

Watts PC, Kay ZM, Wolfenden D, Fox CJ, Geffen AJ, Kemp SJ & Nash RDM (2010) Temporal patterns of spatial genetic structure and effective population size in European plaice (*Pleuronectes platessa*) along the west coast of Scotland and in the Irish Sea. *ICES Journal of Marine Science*, **67**, 607-616. doi:10.1093/icesjms/fsp274

Weltz K, Lyle JM, Semmens JM & Ovenden JR (2018) Population genetics of the endangered Maugean skate (*Zearaja maugeana*) in Macquarie Harbour, Tasmania. *Conservation Genetics*, **19**, 1505-1512. doi:10.1007/s10592-018-1117-0

Wennerström L, Laikre L, Ryman N, Utter FM, Ghani NIA, André C, DeFaveri J, Johansson D, Kautsky L, Merilä N, Pereyra R, Sandström A, Teacher AGF, Wenne R, Vasemägi A, Zbawicka M, Johannesson K & Primmer CR (2013) Genetic biodiversity in the Baltic Sea: Species-specific patterns challenge management. *Biodiversity and Conservation*, **22**, 3045-3065. doi:10.1007/s10531-013-0570-9

Wennevik V, Jørstad KE, Dahle G & Fevolden S (2008) Mixed stock analysis and the power of different classes of molecular markers in discriminating coastal and oceanic Atlantic cod (*Gadus morhua* L.) on the Lofoten spawning grounds, Northern Norway. *Hydrobiologica*, **606**, 7-25.

Westerman ME, Buonaccorsi VP, Stannard JA, Galver L, Taylor C, Lynn EA, Kimbrell CA & Vetter RD (2005) Cloning and characterization of novel microsatellite DNA markers for the grass rockfish, *Sebastes rastrelliger*, and cross-species amplification in 10 related *Sebastes* spp. *Molecular Ecology Notes*, **5**, 74-76. doi:10.1111/j.1471-8286.2004.00837.x

White TA, Stamford J & Hoelzel AR (2009) Nine new microsatellite loci for the orange roughy (*Hoplostethus atlanticus*). *Conservation Genetics*, **10**, 601-603. doi:10.1007/s10592-008-9587-0

White TA, Fotherby HA & Hoelzel AR (2011b) Comparative assessment of population genetics and demographic history of two congeneric deep sea fish species living at different depths. *Marine Ecology Progress Series*, **434**, 155-164. doi:10.3354/meps09207

White TA, Fotherby HA, Stephens PA & Hoelzel AR (2011a) Genetic panmixia and demographic dependence across the North Atlantic in the deep-sea fish, blue hake (*Antimora rostrata*). *Heredity*, **106**, 690-699.

Whitney NM, Robbins WD, Schultz JK, Bowen BW & Holland KN (2012) Oceanic dispersal in a sedentary reef shark (*Triaenodon obesus*): Genetic evidence for extensive connectivity without a pelagic larval stage. *Journal of Biogeography*, **39**, 1144-1156. doi:10.1111/j.1365-2699.2011.02660.x

Wildes SL, Vollenweider JJ, Nguyen HT & Guyon JR (2011) Genetic variation between outer-coastal and fjord populations of Pacific herring (*Clupea pallasii*) in the eastern Gulf of Alaska. *Fishery Bulletin*, **109**, 382-393.

Williams DA, Purcell J, Hughes CR & Cowen RK (2003) Polymorphic microsatellite loci for population studies of the bicolor damselfish, *Stegastes partitus* (Pomacentridae). *Molecular Ecology Notes*, **3**, 547-549. doi:10.1046/j.1471-8286.2003.00506.x

Williams DA, Purcell J, Cowen RK & Hughes CR (2004a) Microsatellite multiplexes for high-throughput genotyping of French grunts (*Haemulon flavolineatum*, Pisces: Haemulidae) and their utility in other grunt species. *Molecular Ecology Notes*, **4**, 46-48. doi:10.1046/j.1471-8286.2003.00568.x

Williams DA, Purcell J, Cowen RK & Hughes CR (2004b) Characterization of microsatellite multiplexes for population genetic studies of bluehead wrasse (*Thalassoma bifasciatum*, Pisces: Labridae). *Molecular Ecology Notes*, **4** 525-527. doi:10.1111/j.1471-8286.2004.00713.x

Wilson AB (2006) Genetic signature of recent glaciation on populations of a nearshore marine fish species (*Syngnathus leptorhynchs*). *Molecular Ecology*, **15**, 1857-1871. doi:10.1111/j.1365-294X.2006.02911.x

Wilson RE, Sage GK, Wedemeyer K, Sonsthagen SA, Menning DM, Gravley MC, Sexson MG, Nelson RJ & Talbot SL (2019) Micro-geographic population genetic structure within Arctic cod (*Boreogadus saida*) in Beaufort Sea of Alaska. *ICES Journal of Marine Science*, **76**, 1713-1721. doi:10.1093/icesjms/fsz041

Winters KL, van Herwerden L, Choat JH & Robertson DR (2010) Phylogeography of the Indo-Pacific parrotfish *Scarus Psittacus*: Isolation generates distinctive peripheral populations in two oceans. *Marine Biology*, **157**, 1679-1691. doi:10.1007/s00227-010-1442-4

Withler RE, King JR, Marliave JB, Beaith B, Li S, Supernault KJ & Miller KM (2004) Polygamous mating and high levels of genetic variation in lingcod, *Ophiodon elongatus* of the Strait of Georgia, British Columbia. In: Genetics of Subpolar Fish and Invertebrates. *Developments in Environmental Biology of Fishes*, vol 23. Springer, Dordrecht. doi:10.1007/978-94-007-0983-6_28

Woodall LC, Koldewey JH & Shaw PW (2011) Historical and contemporary population genetic connectivity of the European short-snouted seahorse *Hippocampus hippocampus* and implications for management. *Journal of Fish Biology*, **78**, 1738-1756. doi:10.1111/j.1095-8649.2011.02974.x

Woodall LC, Koldewey HJ, Boehm JT & Shaw PW (2015) Past and present drivers of population structure in a small coastal fish, the European long snouted seahorse *Hippocampus guttulatus*. *Conservation Genetics*, **16**, 1139-1153. doi:10.1007/s10592-015-0728-y

Wu Z, Xu Q, Zhu J, Dai X & Xu L (2014) Genetic population structure of the bigeye tuna *Thunnus obesus* in the central Pacific Ocean based on mtDNA Cyt*b* sequences. *Fisheries Science*, **80**, 415-426. doi:10.1007/s12562-014-0712-3

Xiao Y, Zhang Y, Gao T, Yanagimoto T, Yabe M & Sakurai Y (2009) Genetic diversity in the mDNA control region and population structure in the small yellow croaker *Larimichthys polyactis*. *Environmental Biology of Fishes*, **85**, 303-314. doi:10.1007/s10641-009-9497-0

Xiao Y, Gao T, Zhang Y & Yanagimoto T (2010) Demographic history and population structure of blackfin flounder (*Glyptocephalus stelleri*) in Japan revealed by mitochondrial control region sequences. *Biochemical Genetics*, **48**, 402-417. doi:10.1007/s10528-009-9321-8

Xiao Y, Zhang Y, Yanagimoto T, Li J, Xiao Z, Gao T, Xu S & Ma D (2011) Population genetic structure of the point-head flounder, *Cleisthenes herzensteini*, in the Nothwestern Pacific. *Genetica*, **139**, 187-198. doi:10.1007/s10709-010-9536-y

Xiao Y, Song N, Li J, Xiao Z & Gao T (2015) Significant population genetic structure detected in the small yellow croaker *Larimichthys polyactis* inferred from mitochondrial control region. *Mitochondrial DNA*, **26**, 409-419. doi:10.3109/19401736.2013.843076

Xiao Y, Li J, Ren G, Ma D, Wang Y, Xiao Z & Xu S (2016) Pronounced population genetic differentiation in the rock bream *Oplegnathus fasciatus* inferred from mitochondrial DNA sequences. *Mitochondrial DNA Part A*, **27**, 2045-2052. doi:10.3109/19401736.2014.982553

Xing S, Shao C, Liao X, Tian Y & Chen S (2009a) Isolation and characterization of polymorphic microsatellite loci from a dinucleotide-enriched genomic library of spotted maigre (*Nibea albiflora*). *Conservation Genetics*, **10**, 789-791. doi:10.1007/s10592-008-9663-

Xing S, Xu G, Liao X, Yang G & Chen S (2009b) Twelve polymorphic microsatellite loci from a dinucleotide-enriched genomic library of Japanese Spanish mackerel (*Scomberomorus niphonius*). *Conservation Genetics*, **10**, 1167-1169. doi:10.1007/s10592-008-9735-6

Xu G-B, Tian Y-S, Liao X-L & Chen S-L (2009) Isolation and characterization of polymorphic microsatellite loci from bluefin leatherjacket (*Navodon septentrionalis* Gunther, 1877). *Conservation Genetics*, **10**, 1181-1184. doi:10.1007/s10592-008-9739-2

Xu D, Li S, Lou B, Zhang Y, Zhan W & Shi H (2012) Genetic diversity in two Japanese flounder populations from China seas inferred using microsatellite markers and COI sequences. *Chinese Journal of Oceanology and Limnology*, **30**, 604-610. doi:10.1007/s00343-012-1197-5

Xu H, Zhang Y, Xu D, Lou B, Guo Y, Sun X & Guo B (2014) Genetic population structure of miiuy croaker (*Miichthys miiuy*) in the Yellow and East China Seas base on mitochondrial COI sequences. *Biochemical Systematics and Ecology*, **54**, 240-246. doi:10.1016/j.bse.2014.01.013

Xu D, Lou B, Zhou W, Chen R, Zhan W & Liu F (2017) Genetic diversity and population differentiation in the yellow drum *Nibea albiflora* along the coast of the China Sea. *Marine Biology Research*, **13**, 456-462. doi:10.1080/17451000.2016.1274033

Yagishita N & Kobayashi T (2008) Isolation and characterization of nine microsatellite loci from the chub mackerel, *Scomber japonicus* (Perciformes, Scombridae). *Molecular Ecology Resources*, **8**, 302-304.

Yamanaka KL & Lacko LC (2001) *Inshore rockfish (*Sebastes ruberrimus, S. maliger, S. caurinus, S. melanops, S. nigrocinctus, *and* S. nebulosus*): stock assessment for the west coast of Canada and recommendations for management.* Canadian Science Advisory Secretariat, 2001.

Yang S, Wang L, Zhang Y, Liu XC, Lin HR & Meng ZN (2011) Development and characterization of 32 microsatellite loci in the giant grouper *Epinephelus lanceolatus* (Serranidae). *Genetics and Molecular Research*, **10**, 4006-4011. doi:10.4238/2011.December.12.3

Yin L, Zhang H, Yanagimoto T & Gao T (2012) Isolation and characterization of nine polymorphic microsatellite markers of the marbled rockfish *Sebastiscus marmoratus* (Scorpaeniformes, Scorpaenidae). *Russian Journal of Genetics*, **48**, 1264-1266.

Yokoyama E, Sakamoto T, Sugaya T & Kitada S (2006) Six polymorphic microsatellite loci in the Japanese Spanish mackerel, *Scomberomorus niphonius*. *Molecular Ecology Notes*, **6**, 323-324. doi:10.1111/j.1471-8286.2005.01217.x

Yoshida K, Nakagawa M & Wada S (2005) Multiplex PCR system applied for analysing microsatellite loci of Schlegel's black rockfish, *Sebastes schlegeli*. *Molecular Ecology Notes*, **5**, 416-418. doi:10.1111/j.1471-8286.2005.00945.x

Young EF, Belchier M, Hauser L, Horsburgh GJ, Meredith MP, Murphy EJ, Pascoal S, Rock J, Tysklind N & Carvalho GR (2015) Oceanography and life history predict contrasting genetic population structure in two Antarctic fish species. *Evolutionary Applications*, **8**, 486-505. doi:10.1111/eva.12259

Yu Z, Kong X, Guo T, Jiang Y, Zhuang Z & Jin X (2005) Mitochondrial DNA sequence variation of Japanese anchovy *Engraulis japonicus* from the Yellow Sea and East China Sea. *Fisheries Science*, **71**, 299. doi:10.1111/j.1444-2906.2005.00964.x

Zane L, Marcato S, Bargelloni L, Bortolotto E, Papetti C, Simonato M, Varotto V & Patarnello T (2006) Demographic history and population structure of the Antarctic silverfish *Pleuragramma antarcticum*. *Molecular Ecology*, **15**, 4499-4511. doi:10.1111/j.1365-294X.2006.03105.x

Zatcoff MS, Ball AO & Sedberry GR (2004) Population genetic analysis of red grouper, *Epinephelus morio*, and scamp, *Mycteroperca phenax*, from the southeastern U.S. Atlantic and Gulf of Mexico. *Marine Biology*, **144**, 769-777. doi:10.1007/s00227-003-1236-z

Zhang Y, Pham NK, Zhang H, Lin J & Lin Q (2014a) Genetic variations in two seahorse species (*Hippocampus mohnikei* and *Hippocampus Trimaculatus*): Evidence for middle Pleistocene population expansion. *PLoS ONE*, **9**, e105494. doi:10.1371/journal.pone.0105494

Zhang H, Yanagimoto T, Zhang X, Song N & Gao T (2014) Lack of population genetic differentiation of a marine ovoviviparous fish *Sebastes schlegelii* in Northwestern Pacific. *Mitochondrial DNA Part A*, **27**, 1748-1754. doi:10.3109/19401736.2014.963797

Zhao L, Shao C, Liao X, Ma H, Zhu X & Chen S (2009a) Twelve novel polymorphic microsatellite loci for the yellow grouper (*Epinephelus awoara*) and cross-species amplifications. *Conservation Genetics*, **10**, 743-745. doi:10.1007/s10592-008-9635-9

Zhao L, Shao C, Liao X & Chen S (2009b) Isolation and characterization of polymorphic microsatellite loci from a dinucleotide-enriched genomic library of seven-band grouper (*Epinephelus septemfasciatus*) and cross-species amplification. *Conservation Genetics*, **10**, 627-629. doi:10.1007/s10592-008-9593-2

Zhao R, Liu WC, Liu M, Zhang SY & Wang RX (2009) Molecular Ecology Resources Primer Development Consortium. Permanent genetic resources added to molecular ecology resources database, 1 May 2009-31 July 2009. *Molecular Ecology Resources*, **9**, 1460-1466. doi:10.1111/j.1755-0998.2009.02759.x

Zheng W, Zou L & Han Z (2015) Genetic analysis of the populations of Japanese anchovy *Engraulis japonicus* from the Yellow Sea and East China Sea based on mitochondrial cytochrome *b* sequence. *Biochemical Systematics and Ecology*, **58**, 169-177. doi:10.1016/j.bse.2014.12.007
